# The Hematopoietic TALE-Code Shows Normal Activity of IRX1 in Myeloid Progenitors and Reveals Ectopic Expression of IRX3 and IRX5 in Acute Myeloid Leukemia

**DOI:** 10.3390/ijms23063192

**Published:** 2022-03-16

**Authors:** Stefan Nagel, Claudia Pommerenke, Corinna Meyer, Roderick A. F. MacLeod

**Affiliations:** Department of Human and Animal Cell Lines, Leibniz-Institute DSMZ, German Collection of Microorganisms and Cell Cultures, 38124 Braunschweig, Germany; cpo14@dsmz.de (C.P.); cme@dsmz.de (C.M.); rafmacleod@gmail.com (R.A.F.M.)

**Keywords:** homeodomain, HOX-code, NKL-code, PBX1, TALE-code, TBX-code

## Abstract

Homeobox genes encode transcription factors that control basic developmental decisions. Knowledge of their hematopoietic activities casts light on normal and malignant immune cell development. Recently, we constructed the so-called lymphoid TALE-code that codifies expression patterns of all active TALE class homeobox genes in early hematopoiesis and lymphopoiesis. Here, we present the corresponding myeloid TALE-code to extend this gene signature, covering the entire hematopoietic system. The collective data showed expression patterns for eleven TALE homeobox genes and highlighted the exclusive expression of IRX1 in megakaryocyte-erythroid progenitors (MEPs), implicating this TALE class member in a specific myeloid differentiation process. Analysis of public profiling data from acute myeloid leukemia (AML) patients revealed aberrant activity of IRX1 in addition to IRX3 and IRX5, indicating an oncogenic role for these TALE homeobox genes when deregulated. Screening of RNA-seq data from 100 leukemia/lymphoma cell lines showed overexpression of IRX1, IRX3, and IRX5 in megakaryoblastic and myelomonocytic AML cell lines, chosen as suitable models for studying the regulation and function of these homeo-oncogenes. Genomic copy number analysis of IRX-positive cell lines demonstrated chromosomal amplification of the neighboring IRX3 and IRX5 genes at position 16q12 in MEGAL, underlying their overexpression in this cell line model. Comparative gene expression analysis of these cell lines revealed candidate upstream factors and target genes, namely the co-expression of GATA1 and GATA2 together with IRX1, and of BMP2 and HOXA10 with IRX3/IRX5. Subsequent knockdown and stimulation experiments in AML cell lines confirmed their activating impact in the corresponding IRX gene expression. Furthermore, we demonstrated that IRX1 activated KLF1 and TAL1, while IRX3 inhibited GATA1, GATA2, and FST. Accordingly, we propose that these regulatory relationships may represent major physiological and oncogenic activities of IRX factors in normal and malignant myeloid differentiation, respectively. Finally, the established myeloid TALE-code is a useful tool for evaluating TALE homeobox gene activities in AML.

## 1. Introduction

Stem and progenitor cells expand and subsequently pass through several developmental stages to differentiate into mature cells and tissues. In the hematopoietic system, corresponding processes generate all types of blood and immune cells, starting with hematopoietic stem cells and their derived progenitors, which establish the lymphoid and myeloid lineage [1]. The first produces lymphocytes such as B- and T-cells, while the latter generates inter alia granulocytes, monocytes, megakaryocytes, and erythrocytes. The myeloid system contains several progenitors that differ in their developmental potential. The common myeloid progenitor (CMP) is able to produce all types of myeloid cells, while the megakaryocyte-erythroid progenitors (MEPs) are developmentally restricted, generating either megakaryocytes or erythrocytes.

Hematopoietic differentiation processes are typically regulated at the transcriptional level [2,3,4]. Therefore, the master genes controlling these operations mostly encode transcription factors (TFs). Homeobox genes encode developmental TFs, controlling basic decisions in cell and tissue differentiation. They contain a homeodomain at the protein level, which consists of three helices. This domain interacts with DNA, cofactors, and chromatin, thus representing a platform for gene-regulating activities [5]. According to sequence similarities of their conserved homeobox, these genes fall into eleven classes and several subclasses [6]. ANTP represents the largest class containing 39 clustered HOX genes and the 48-member-strong NKL homeobox gene subclass. The class of TALE homeobox genes comprises specific homeodomain factors that share a three-amino-acid residue loop extension (abbreviated as TALE) between helix 1 and helix 2. The human genome encodes 20 TALE homeodomain proteins including six IRX-factors and the well-known PBX1 and MEIS1 members, which are able to cooperate with HOX factors [7,8,9].

The human IRX genes are genomically arranged in two clusters, consisting of IRX1, IRX2, and IRX4 (located at chromosomal position 5p15) and of IRX3, IRX5, and IRX6 (16q12). This clustering is evolutionarily conserved and may be related to conjoint transcriptional regulation [10]. IRX genes are embryonically expressed and tasked with regulating the development of particular tissues and organs [11]. IRX1, for example, regulates the development of the limbs, gut, kidney, and lung and plays an oncogenic role in several types of cancer [12]. Aberrant expression of IRX1 and IRX3 has been reported in myeloid leukemia, supporting general commitments in carcinogenesis [13,14].

Reflecting their physiological functions in development, deregulated homeobox genes drive specific hematopoietic malignancies and are frequently targeted by chromosomal aberrations [15,16,17]. To evaluate the activities of major subgroups of homeobox genes in lymphoid and myeloid malignancies, we generated the “NKL-code” [18]. This gene signature describes physiological activities of NKL homeobox genes during hematopoiesis and assists the identification of deregulated NKL homeobox gene expression in patients. Accordingly, we also described the lymphoid TALE-code and applied that gene signature to identify the aberrant activity of TALE homeobox gene PBX1 in Hodgkin lymphoma [19]. Here, we extended the established lymphoid TALE-code to the entire hematopoietic system and included the myeloid lineage.

Acute myeloid leukemia (AML) is the most frequent hematopoietic malignancy in adults. The tumor cells derive from particular myeloid progenitors. Several subtypes of AML may be distinguished according to originating cells and stages, phenotypes, chromosomal aberrations, and gene mutations that differ in prognosis and outcome [20]. Knowledge of normal and abnormal activities of basic developmental regulators should serve to refine diagnostic procedures and thus help identify novel therapeutic targets. Accordingly, we describe here the physiological expression of TALE homeobox genes during myelopoiesis and investigate the aberrant activities of IRX homeobox genes in AML subsets.

## 2. Results

### 2.1. Establishment of the Myeloid TALE-Code

**Figure 1:**
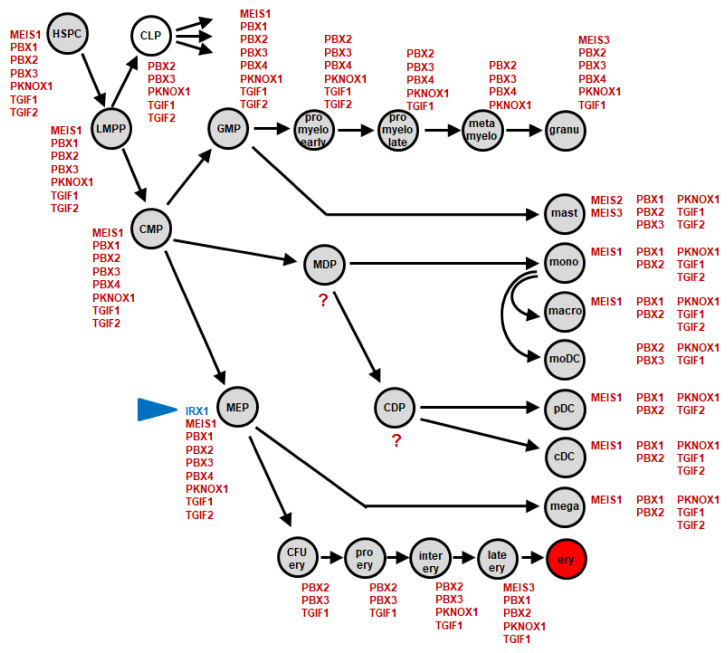
Myeloid TALE-code. This diagram summarizes the screening results for expression analyses of TALE homeobox genes (red) in early hematopoiesis and myelopoiesis. We have termed this expression pattern myeloid TALE-code. Expression of IRX1 is highlighted in blue (arrowhead). Abbreviations: cDC, conventional dendritic cell; CDP, common dendritic progenitor; CFU ery, erythroid colony forming unit; CLP, common lymphoid progenitor; CMP, common myeloid progenitor; ery, erythrocyte; GMP, granulo-myeloid progenitor; granu, granulocyte; HSPC, hematopoietic stem and progenitor cell; inter ery, intermediate stage erythroblast; late ery, pyknotio-stage erythroblast; LMPP, lymphomyelo-primed progenitor; macro, macrophage; mast, mast cell; MDP, monocyte dendritic cell progenitor; mega, megakaryocyte; MEP, megakaryocytic-erythroid progenitor; metamyelo, metamyelocyte; moDC, monocyte-derived dendritic cell; mono, monocyte; pDC, plasmacytoid dendritic cell; pro ery, pro-erythroblast; pro myelo early/late, early/late promyelocyte.

Recently, we delineated the lymphoid TALE-code for early hematopoiesis and lymphopoiesis [19]. Here, we extended this gene signature to include the myeloid system according to our reported approach generating the myeloid NKL-code [21]. By exploiting public gene expression profiling and RNA-seq datasets, we generated a TALE homeobox gene expression pattern for myeloid progenitors and mature cells (Figure S1). The applied cutoffs to discriminate positive and negative expression levels were adopted from our previous studies [21]. The results are depicted in Figure 1, representing the myeloid TALE-code.

The established myeloid TALE-code comprises eleven genes: IRX1, MEIS1, MEIS2, MEIS3, PBX1, PBX2, PBX3, PBX4, PKNOX1, TGIF1, and TGFI2. We found TALE homeobox gene activities in all cell types analyzed. The numbers of expressed genes in single entities ranged from three (pro-erythroblast) to nine (MEP). TGIF genes were expressed in all but one entity (metamyelocyte). PBX1 was also active in most mature cell types, contrasting with the findings for this gene in lymphopoiesis [19]. Interestingly, IRX1 expression was only detected in MEPs, while the remaining IRX genes remained silent in the complete hematopoietic compartment [19]. This observation may indicate that IRX1 plays a major role in early differentiation processes of this particular progenitor, which generates both megakaryocytes and erythrocytes. In accordance with the TALE-code, RNA-seq gene expression data from the Human Protein Atlas showed repression of all six IRX genes in the myeloid entities represented, despite expression in cells and tissues of other compartments (Figure S2). In the following, we examined a potential oncogenic role for IRX genes in AML.

### 2.2. Aberrant Expression of IRX1, IRX3, and IRX5 in AML

To scrutinize the aberrant expression of IRX genes in AML patients, we screened three public expression profiling datasets. Dataset GSE19577 covers 42 AML patients with MLL-rearrangements, GSE15434 contains 251 patients with normal karyotypes, and GSE6891 contains 537 patients with unknown karyotypes and specific recurrent chromosomal translocations and/or gene mutations. Our results are shown in Figure S3. In all datasets, we detected aberrant expressions of IRX1, IRX3, and IRX5, while IRX2 and IRX4 showed only inconsistent or low expression levels. Of note, IRX6 was not represented by the applied arrays for these datasets. Thus, we detected the aberrant overexpression of IRX1 and ectopic activity of IRX3 and IRX5 in AML patient subsets.

IRX1 is located at chromosomal locus 5p15, while IRX3 and IRX5 are neighbors at 16q12, indicating possible coregulation. The analysis of co-expression for IRX1, IRX3, and IRX5 in AML patients revealed significant correlations for IRX3 and IRX5 using datasets GSE15434 and GSE6891 (Figure S4). Thus, the combined expression of IRX3 and IRX5 may be mediated by their genomic proximity, albeit lacking in patients with MLL-rearrangements. Taken together, TALE homeobox genes IRX1, IRX3, and IRX5 are aberrantly expressed in AML patient subsets, which differ in karyotypes and gene mutations.

**Figure 2:**
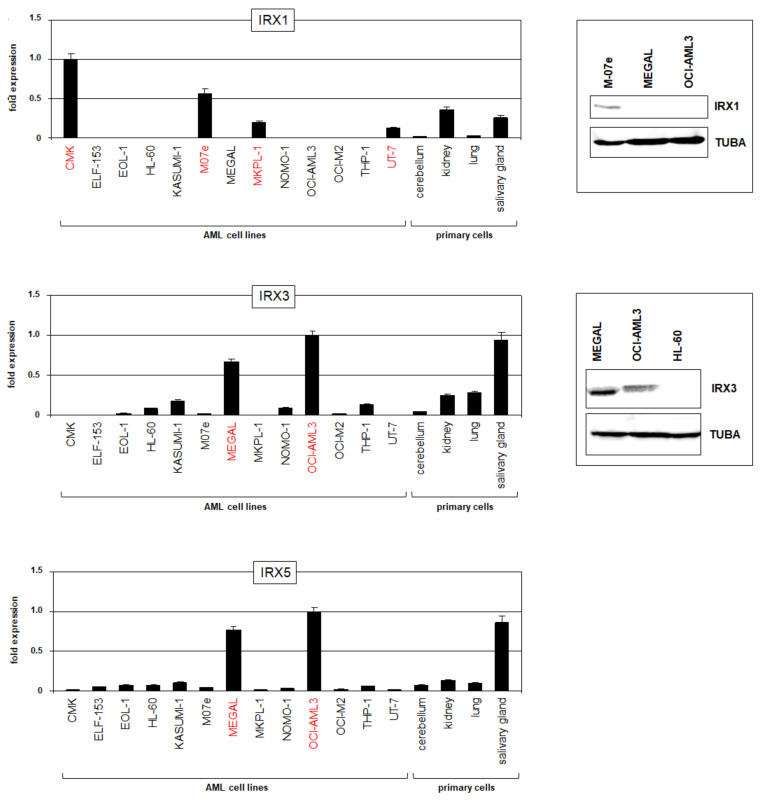
IRX gene activities in AML cell lines. Expression analyses of TALE homeobox genes IRX1, IRX3, and IRX5 in AML cell lines and primary controls as performed by RQ-PCR (**left**). Cell lines showing significant expression levels of the corresponding IRX genes are shown in red. P-values are indicated by asterisks. Western blot analyses (**right**) were performed for IRX1 and IRX3 in selected AML cell lines. TUBA served as loading control.

To find suitable models for functional studies, we screened IRX gene activities using our published RNA-seq dataset LL-100, which contains 34 myeloid and 66 lymphoid leukemia/lymphoma cell lines [22]. Significant RNA expression levels in myeloid cell lines were detected just for IRX1, IRX3, and IRX5 (Figure S5), corresponding to the AML patient data. Interestingly, the IRX1 expression was restricted to cell lines derived from megakaryoblastic AML, while expressions of both IRX3 and IRX5 were elevated in cell lines derived from myelomonocytic AML. However, megakaryoblastic cell line MEGAL expressed IRX3 and IRX5 but not IRX1, representing an exception to that rule. RQ-PCR analysis of selected AML cell lines confirmed the RNA-seq data, showing IRX1 expressions in CMK, M07e, MKPL1, and UT-7 and IRX3/IRX5 expressions in MEGAL and OCI-AML3 (Figure 2). An additional comparison with primary cells from the cerebellum, kidney, lung, and salivary gland showed similar or even higher RNA expression levels in the cell lines, demonstrating significant overexpression of these genes in AML (see also Figure S2). Finally, Western blot analysis of IRX1 and IRX3 confirmed the transcript data at the protein level (Figure 2), endorsing the analyzed cell lines as models for functional studies.

### 2.3. Chromosomal and Genomic Analyses of the Gene Loci for IRX1, IRX3, and IRX5 in AML

**Figure 3:**
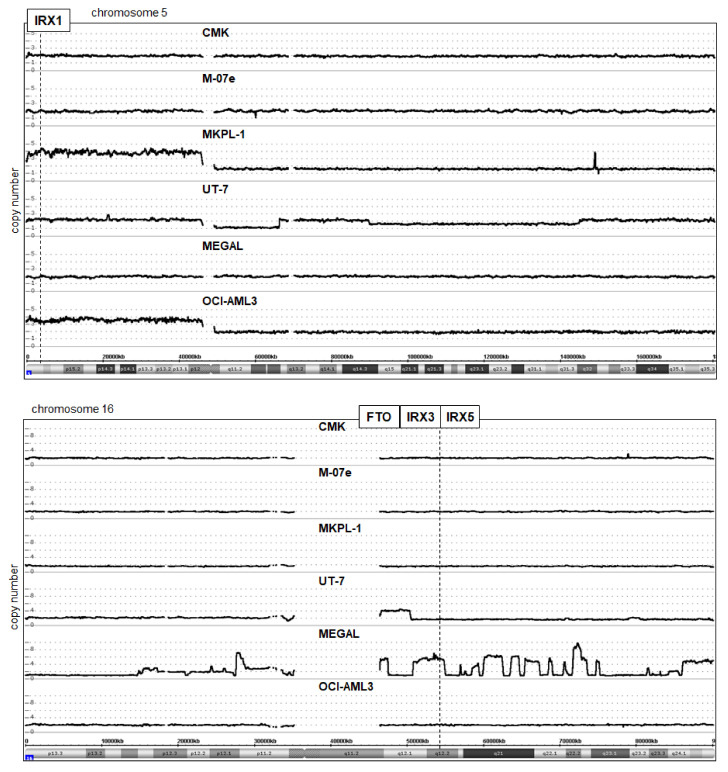
Genomic analysis of AML cell lines. Copy number states for chromosome 5 (**above**) and chromosome 16 (**below**) of IRX1-positive cell lines CMK, M-07e, MKPL-1, and UT-7, and of IRX3/IRX5-positive AML cell lines MEGAL and OCI-AML3 were determined by genomic profiling analysis. A copy number gain for IRX1 (located at 5p15) was detected in MKPL-1 and OCI-AML3. Amplification of FTO, IRX3, and IRX5 (16q12) was detected among multiple complex chromosome 16 rearrangements in MEGAL.

In hematopoietic malignancies, aberrantly activated oncogenes including homeobox genes are frequently targeted by chromosomal rearrangements [15,16]. To analyze if this deregulating mechanism underlies the activation of IRX genes in AML, we inspected the published karyotypes of the corresponding cell lines (www.DSMZ.de, accessed on 15 February 2022) and shortlisted i(5)(p10) in OCI-AML3 and MKPL-1 and del(16)(q13q23) in MEGAL, which may have activating impacts. In addition, we performed genomic profiling analysis of AML cell lines CMK, M-07e, MKPL-1, UT-7, MEGAL, and OCI-AML3. The data for chromosomes 5 and 16 are shown in Figure 3. IRX1 (5p15) is duplicated together with the whole short arm in OCI-AML3 and MKPL-1, consistent with the karyotype data showing the formation of isochromosomes for the short arms. However, OCI-AML3 is IRX1-negative, discounting the likely contribution of this aberration to its activation. In contrast, IRX3 and IRX5 are located at 16q12 and considerably amplified in MEGAL. The data showed several amplicons covering nearly the complete long arm of chromosome 16. Moreover, RNA-seq data for the transcribed enhancer locus FTO, which is located adjacent to IRX3 and regulates IRX3/IRX5 activity [23,24], showed enhanced expression restricted to MEGAL (Figure S5). Thus, genomic amplification of IRX3/IRX5 together with FTO may underlie their activation in megakaryoblastic AML cell line MEGAL. Despite these substantial rearrangements of chromosome 16 in MEGAL, RT-PCR excluded the generation of AML-specific fusion gene CBFb-MYH11 via inv(16)(p13q22), confirming the cytogenetic findings (Figure S6A).

### 2.4. GATA1 and GATA2 Activate IRX1 in AML Cell Lines

To identify potential regulators and target genes of aberrantly expressed IRX1, we compared LL-100 RNA-seq expression data from IRX1-positive (CMK, M07e, MKPL-1, and UT-7) with IRX3/IRX5-positive (MEGAL and OCI-AML3) AML cell lines. The results are shown in Table S1. Gene set annotation analysis of the top-1000 upregulated genes in IRX1-positve cell lines revealed the statistically significant GO-term erythrocyte differentiation (*p* = 0.0052) and the associated master genes GATA1, GATA2, KLF1, and TAL1, which were chosen for analysis in more detail.

**Figure 4:**
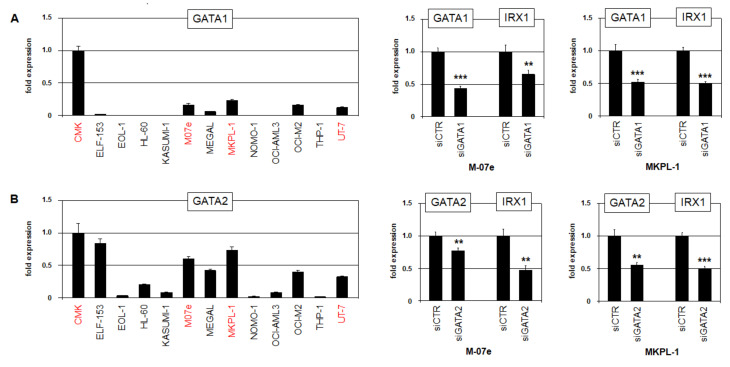
Transcriptional regulation of IRX1. Expression analysis of (**A**) GATA1 and (**B**) GATA2 by RQ-PCR in selected AML cell lines (**left**). Transcript levels are indicated in relation to CMK. Cell lines expressing significant levels of IRX1 are highlighted in red. SiRNA-mediated knockdown of (**A**) GATA1 and (**B**) GATA2 demonstrates activations of IRX1 and IRX5 in AML cell lines M-07e and MKPL-1 (**right**). *p*-values are indicated by asterisks (** *p* < 0.01, *** *p* < 0.001).

In developing imaginal discs of the fruit fly Drosophila melanogaster, GATA factor pannier regulates the IRX-cluster Iro-C [25], spotlighting this relationship for consideration in humans. RQ-PCR analysis of GATA1 and GATA2 confirmed the higher expression and thus correlating IRX1 levels in cell lines CMK, M07e, MKPL-1, and UT-7. In addition, the siRNA-mediated knockdown of GATA1 and GATA2 in M-07e and MKPL-1 cells resulted in the concomitant downregulation of IRX1 (Figure 4), demonstrating an activating impact of both factors.

Our genomic profiling data showed for cell line CMK a duplication of the short arm of chromosome X, which includes the GATA1 locus at Xp11 (Figure S7). CMK expressed the highest GATA1 RNA levels, indicating that this genomic aberration may contribute indirectly to IRX1 activation. An additional chromosomal aberration revealed by genomic profiling was an amplification at 19p13 in cell lines MKPL-1 and UT-7 (Figure S7). This amplicon includes the erythropoietin receptor encoding gene EPOR and may underlie its overexpression, as shown by RNA-seq data (Figure S5). EPOR and GATA1 are mutual activators and thus may also support IRX1 expression [26]. Finally, expression profiling data from various myelopoietic stages (dataset GSE42519) showed elevations of GATA1, GATA2, and EPOR in IRX1-positive MEPs, while earlier progenitors and later myeloid stages expressed decreased levels (Figure S8). Thus, master factors GATA1 and GATA2 are prominently co-expressed along with IRX1 in MEPs and megakaryoblastic AML cell lines, and activates IRX1 expression.

### 2.5. HOXA10 and BMP2-Signaling Activate IRX3 and IRX5 in AML

To identify potential regulators and target genes of aberrantly expressed IRX3 and IRX5, we then compared RNA-seq data from IRX3/IRX5-positive with IRX1-positive AML cell lines (Table S2). Statistically significant GO-terms identified by gene set annotation analysis of the top-1000 upregulated genes in IRX3/IRX5-positive cell lines included the negative regulation of myeloid cell differentiation (*p* = 0.0029), SMAD protein signal transduction (*p* = 0.05), and negative regulation of apoptotic processes (*p* = 0.00007). Among the top-1000 upregulated genes, we chose the following candidates for further studies: BCL2, BMP2, HOXA10, and NUP214.

Elevated NUP214 expression correlated with the presence of fusion gene SET-NUP214 in MEGAL, which reportedly activates HOXA genes in T-cell leukemia [27,28]. Accordingly, we confirmed the chromosomal deletion at 9q34 by genomic profiling and the generated fusion gene by RT-PCR in this cell line (Figure S6B).

**Figure 5:**
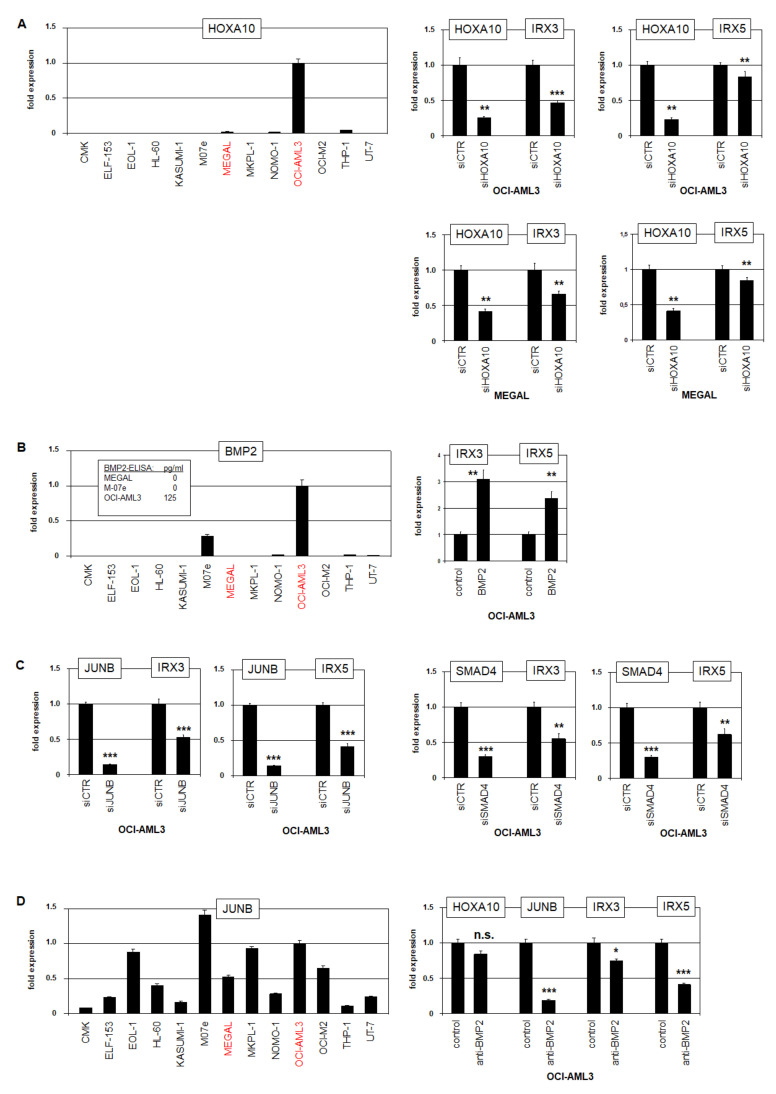
Transcriptional regulation of IRX3 and IRX5. (**A**) RQ-PCR analysis of HOXA10 in AML cell lines (**left**). Transcript levels are indicated in relation to OCI-AML3, and IRX3/IRX5-positive cell lines are indicated in red. RQ-PCR analysis of OCI-AML3 (**above**) and MEGAL (**below**) showed downregulation of IRX3 and IRX5 after siRNA-mediated knockdown of HOXA10 (**right**). (**B**) RQ-PCR analysis of BMP2 in AML cell lines (**left**). Transcript levels are indicated in relation to OCI-AML3. ELISA results for BMP2 protein levels in AML cell line supernatants are inserted. RQ-PCR analysis of OCI-AML3 treated with BMP2 resulted in the upregulation of IRX3 and IRX5 (**right**). (**C**) RQ-PCR analysis of OCI-AML3 after siRNA-mediated knockdown of JUNB (**left**) and SMAD4 (**right**) showed downregulation of IRX3 and IRX5. (**D**) RQ-PCR analysis of JUNB in AML cell lines (**left**). Transcript levels are indicated in relation to OCI-AML3. RQ-PCR analysis of OCI-AML3 treated with inhibitory BMP2-antibody resulted in downregulation of JUNB, IRX3, and IRX5, while HOXA10 was not significantly affected (**right**). *p*-values are indicated by asterisks (* *p* < 0.05, ** *p* < 0.01, *** *p* < 0.001, n.s. not significant).

In AMLs containing MLL-AF4 rearrangements, HOXA genes have been shown to correlate with the expression of IRX3 but not with IRX1 [13,14,29,30,31], suggesting that particular HOXA-members may support IRX3/IRX5 activation. RQ-PCR analysis of HOXA10 showed low and high RNA expression levels in IRX3/IRX5-positive cell lines MEGAL and OCI-AML3, respectively, while IRX1-positive cell lines CMK, M07e, MKPL-1, and UT-7 tested negative (Figure 5A). Furthermore, siRNA-mediated knockdown of HOXA10 in OCI-AML3 and MEGAL resulted in the concomitant downregulation of IRX3 and IRX5, demonstrating an activating input (Figure 5A). Expression profiling data covering multiple myelopoietic stages (dataset GSE42519) showed an elevated HOXA10 expression in early stages, which decreased after the MEP-stage (Figure S8). Thus, HOXA10 was highly expressed in myeloid progenitors including MEPs and activated the expression of IRX3/IRX5 in AML ectopically.

As BMP2-signaling has been shown to regulate IRX gene IRO3 in the organizer region during gastrulation in zebrafish [32], we decided to explore this relationship in humans. RQ-PCR analysis of BMP2 confirmed the conspicuous expression in OCI-AML3 (Figure 5B). Furthermore, quantification of the BMP2 protein in cell culture supernatants revealed significant levels in OCI-AML3, while M-07e and MEGAL tested negative. Additional treatment of OCI-AML3 with recombinant BMP2 upregulated both IRX3 and IRX5 (Figure 5B), demonstrating an activating input. The TFs JUNB and SMAD4 operate downstream in BMP2-signaling [33,34]. Accordingly, the siRNA-mediated knockdown of JUNB and SMAD4 in OCI-AML3 resulted in the concomitant downregulation of IRX3 and IRX5, showing an activating role of these factors (Figure 5C). However, RQ-PCR analysis of JUNB indicated no correlation with IRX3/IRX5 expression (Figure 5D). However, the suppression of BMP2-activity by the treatment of OCI-AML3 with an inhibitory antibody resulted in the concomitant downregulation of IRX3, IRX5, and JUNB, while HOXA10 showed no significant alteration (Figure 5D).

Taken together, we identified HOXA10 and BMP2-signaling via JUNB and SMAD4 as activating factors for IRX3 and IRX5 in AML. The correlation of SET-NUP214, HOXA, and BMP2-signaling with IRX3/IRX5 expression in MEGAL and OCI-AML3, respectively, indicated that diverse aberrant mechanisms may underlie the ectopic activation of these TALE homeobox genes.

### 2.6. IRX1 and IRX3 Differ in Target Gene Regulation

**Figure 6:**
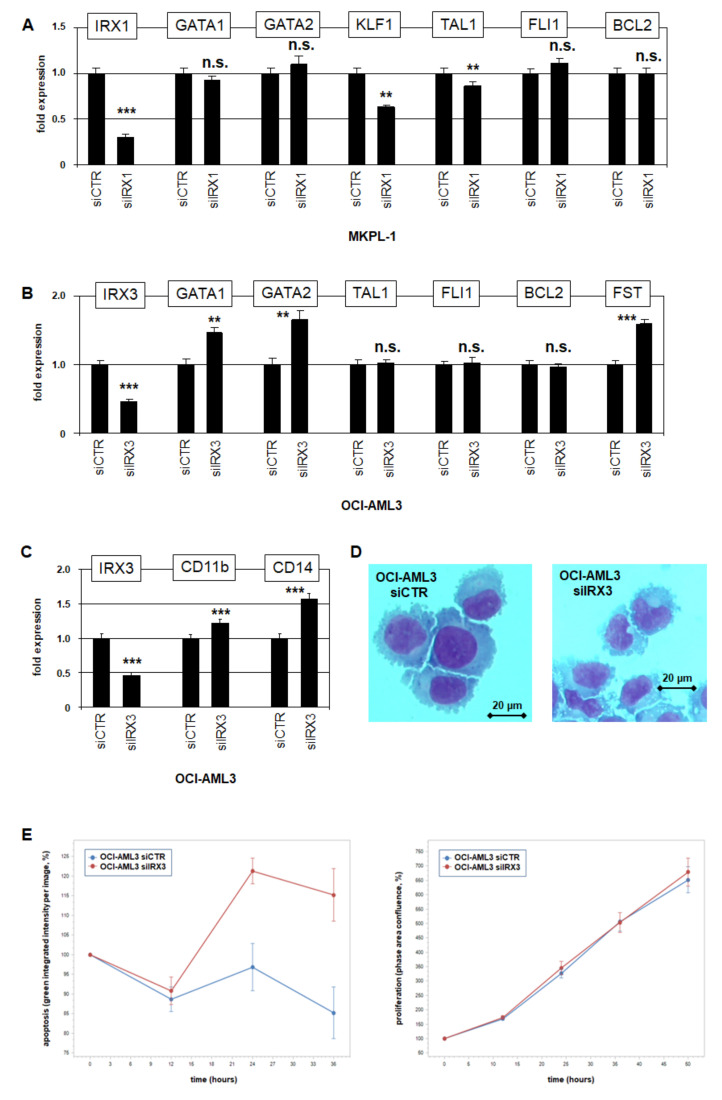
Target gene analyses for IRX1 and IRX3 in AML cell lines. (**A**) RQ-PCR analysis of MKPL-1 treated for siRNA-mediated knockdown of IRX1 resulted in downregulation of KLF1 and TAL1, while GATA1, GATA2, FLI1, and BCL2 remained unaffected. Thus, IRX1 activates KLF1 and TAL1. (**B**) RQ-PCR analysis of OCI-AML3 treated for siRNA-mediated knockdown of IRX3 resulted in upregulation of GATA1, GATA2, and FST, while TAL1, FLI1 and BCL2 remained unaffected. Thus, IRX3 inhibits GATA1, GATA2, and FST. (**C**) RQ-PCR analysis of OCI-AML3 treated for siRNA-mediated knockdown of IRX3 resulted in upregulation of myeloid differentiation marker CD11b/ITGAM and CD14. (**D**) SiRNA-mediated knockdown of IRX3 in OCI-AML3 cells induced morphological alterations of their nuclei, as shown by Giemsa–May–Grünwald staining. (**E**) Live-cell imaging analyses of OCI-AML3 treated for siRNA-mediated knockdown of IRX3 showed increased levels of apoptosis (**left**), while proliferation remained unaffected (**right**). Standard deviations are indicated as bars. *p*-values are indicated by asterisks (** *p* < 0.01, *** *p* < 0.001, n.s. not significant).

For target gene analysis of IRX1 in AML, we tested the erythropoietic differentiation genes identified in megakaryoblastic cell line MKPL-1. The SiRNA-mediated knockdown of IRX1 effected the significant downregulation of KLF1 and TAL1 while sparing GATA1 and GATA2 (Figure 6A). Thus, IRX1 activated the erythroid master TFs KLF1 and TAL1, which may disturb megakaryopoiesis if aberrantly activated. Interestingly, both genes are also prominently expressed in MEPs (Figure S8), suggesting that this regulatory relationship may play a physiological role in these progenitor cells.

To examine if the differentiation factors GATA1, GATA2, KLF1, and TAL1 are regulated by IRX3, we used myelomonocytic cell line OCI-AML3. The SiRNA-mediated knockdown of IRX3 resulted in the elevated expression of GATA1 and GATA2, while TAL1 remained unaffected and KLF1 silent (Figure 6B). Thus, IRX3 suppressed the expression of the myeloid differentiation factors GATA1 and GATA2, unlike erythroid factors TAL1 and KLF1. A potential impact of IRX3 in myeloid development was examined by the quantification of differentiation markers. The knockdown of IRX3 in OCI-AML3 resulted in the significant upregulation of CD11b/ITGAM and CD14 (Figure 6C). Furthermore, morphological inspection of these treated cells showed nuclear alterations indicative for myeloid differentiation (Figure 6D), collectively demonstrating an inhibitory role of IRX3 for these processes.

Functional live-cell imaging analysis of OCI-AML3 cells treated for IRX3-knockdown indicated that IRX3 inhibited apoptosis while sparing proliferation (Figure 6E). However, IRX1 and IRX3 knockdown experiments indicated that this anti-apoptotic effect is not mediated by the transcriptional activation of BCL2 (Figure 6A,B). Of note, BCL2 showed the conspicuous downregulation in MEPs (Figure S8), indicating potential sensitivity to apoptosis of these progenitors and conceivably of any derived malignant cells.

The observed suppressive activity of IRX3 prompted scanning downregulated genes in IRX3/IRX5-positive cell lines as well. This exercise revealed BMP2-inhibitor FST, which was downregulated in OCI-AML3 and MEGAL (Table S1, Figure S5). Accordingly, siRNA-mediated knockdown of IRX3 in OCI-AML3 boosted FST expression (Figure 6B), showing that IRX3 inhibited FST. Thus, IRX3 operated in AML as both gene activator and suppressor.

Recently, we reported that NKL homeodomain factors NKX2-3 and NKX2-4 deregulate megakaryocytic-erythroid differentiation factor FLI1 in AML, which shows conspicuous downregulation in MEPs (Figure S8) [35]. However, knockdown experiments for IRX1 and IRX3 excluded FLI1 deregulation (Figure 6A,B), demonstrating functional differences in downstream activities of NKL and TALE homeo-oncogenes in AML.

Taken together, IRX1 and IRX3 showed significant differences in target gene regulation: IRX1 activated KLF1 and TAL1, while IRX3 inhibited GATA1 and GATA2. However, all represent master TFs involved in megakaryocytic-erythroid differentiation. Furthermore, the IRX3-mediated suppression of FST may create an activating feedback loop as BMP2-signaling supported IRX3 expression.

## 3. Discussion

In this study, we established the myeloid TALE-code, representing a TALE homeobox gene expression pattern in normal myelopoiesis. Thus, we completed this signature for the entire hematopoietic system [19]. According to our previously generated NKL-code, we used the same approach to analyze public expression data for TALE homeobox genes in hematopoiesis [18]. These codes show (i) physiological activities of selected homeobox gene groups in hematopoietic entities, and (ii) a blueprint permitting the evaluation of homeobox gene expressions in corresponding malignancies of patients.

The myeloid TALE-code comprises eleven TALE homeobox genes (Figure 1). Interestingly, one of these, PBX1, was expressed in most progenitors and terminal differentiated myeloid cells and only silenced in granulopoiesis. This expression pattern contrasts with that reported in lymphopoiesis, in which all terminal-differentiated lymphocytes downregulate PBX1 [19]. In accordance with these physiological data, the aberrant maintenance of PBX1 activity in developing B-cells contributes to the generation of pre-B-cell leukemia or Hodgkin lymphoma [19,36,37,38]. In the myeloid lineage, the aberrant expression of PBX1 has been associated with severe congenital neutropenia. Its repression is mediated by GFI1, whose loss reactivates PBX1 in addition to MEIS1 and HOXA genes [39], providing a mechanistic explanation for the aberrant expression of PBX1 in granulopoiesis. Thus, TALE-code data serve to elucidate clinical findings in various hematopoietic malignancies.

In addition, our myeloid TALE-code data revealed a conspicuous expression pattern for IRX1, which was restricted to the MEP stage. This TALE homeobox gene represented the only IRX gene active in normal hematopoiesis. Furthermore, the aberrant expression of IRX genes in AML patients and cell lines was detected for IRX1, IRX3, and IRX5. The deregulated activity of IRX genes in AML has been reported in previous studies as well. Accordingly, the aberrant expression of IRX1 plays an oncogenic role in cases with particular MLL rearrangements, and IRX3 inhibits myelomonocytic differentiation, while IRX2 supports myeloid differentiation [13,14,40]. Thus, the published results together with our data indicate functional differences between IRX factors in AML.

The human genome encodes six IRX genes that are arranged in two clusters. Clustering of the homeobox genes is evolutionarily conserved and often connected with the coregulation of gene neighbors, as described for HOX, DLX, and IRX genes [10,41,42]. They share regulatory elements as demonstrated for IRX3 and IRX5, which are controlled by the transcribed enhancer locus FTO [23,24]. AML cell line MEGAL expressed IRX3 and IRX5 ectopically and showed an amplification of these genes together with FTO at 16q12. This chromosomal aberration was part of a chain of consecutive amplicons at 16q, signifying chromothripsis. This type of cataclysmic genomic rearrangement affects single chromosomes or chromosomal arms and has been described in myeloid and lymphoid malignancies [43,44]. Previously reported aberrations altering chromosome 16 in AML include inv(16)(p13q22) and del(16)(q11) [45,46,47]. However, our data excluded for MEGAL the presence of both del(16)(q11) and inv(16)(p13q22), which generates the fusion gene CBFb-MYH11.

**Figure 7:**
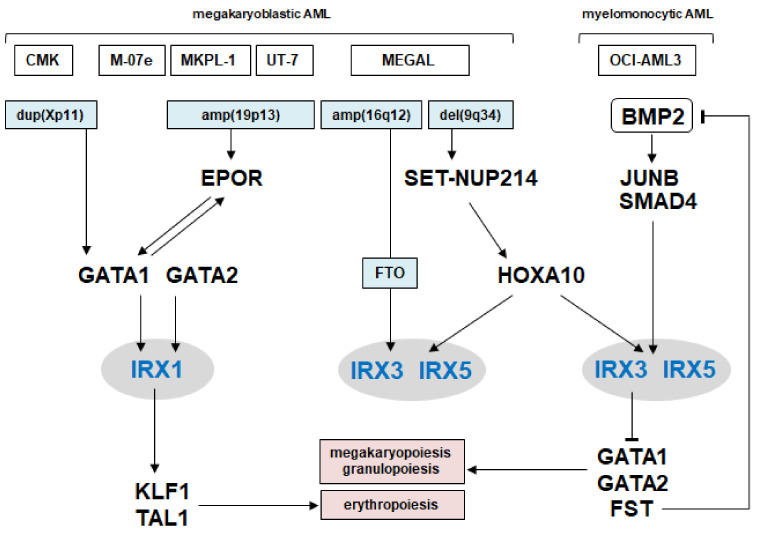
Gene regulatory network of IRX1 and IRX3/IRX5 in AML. The figure summarizes the results of this study. TALE homeobox genes IRX1, IRX3, and IRX5 are located centrally, chromosomal aberrations lie upstream, and developmental TFs and BMP2-signaling components both upstream and downstream. Thus, these IRX genes are part of a regulatory gene network, controlling myeloid differentiation.

Our additional findings concerning the regulation and function of IRX genes in AML are summarized in Figure 7. The data include chromosomal aberrations and involvement of the myeloid master factors GATA1, GATA2, HOXA10, KLF1, and TAL1, generating normal and aberrant gene networks, which control differentiation processes. We showed that GATA1 and GATA2 performed an activating role in IRX1 expression, which, in turn, activated KLF1 and TAL1. In contrast, HOXA10 activated IRX3 and IRX5, while IRX3 inhibited GATA1 and GATA2 expression.

GATA1 and GATA2 are fundamental regulators in myelopoiesis and interact with TAL1 in TF-complexes [48,49,50]. GATA1 drives granulopoiesis, mast cell differentiation, and megakaryopoiesis [51,52,53]. GATA2 plays basic roles in stem and progenitor cells, including erythroid progenitors, and becomes substituted by GATA1 during development [48]. The mutation or downregulation of GATA2 contributes to the generation of myeloid malignancies including AML [48,54,55]. Thus, the downregulation of GATA1 and GATA2 by IRX3 may represent a novel type of oncogenic alteration in subsets of myelomonocytic AML. Consistent with our data, IRX3 blocks myeloid differentiation [14].

HOXA genes including HOXA10 represent key developmental regulators in myelopoiesis, and their deregulation plays a role in acute leukemias containing MLL-rearrangements and SET-NUP214 fusions [27,56]. We showed that HOXA10 activated ectopic expressions of IRX3 and IRX5 in AML. This finding corresponds to reported correlations of IRX3 to elevated and of IRX1 to decreased HOXA levels in AML [14,29,30,31]. Furthermore, we showed that IRX3 and IRX5 were activated by BMP2-signaling via JUNB and SMAD4, thus representing an additional mechanism of IRX gene deregulation. FST inhibits the activity of BMP proteins and thus BMP-signaling [34,57]. BMP4 supports megakaryopoiesis and BMP2 erythropoiesis [58,59], demonstrating the developmental impact of this signaling pathway. In T-cell leukemia, the ectopic activity of CHRDL1 has been shown to inhibit BMP-signaling, which results in the aberrant expression of NKL homeobox gene MSX1 [60]. Thus, aberrant deregulation of the BMP pathway plays a common oncogenic role in leukemia.

KLF1 and TAL1 are master genes for erythroid development and suppressors of megakaryopoiesis [61,62]. Our data showed that both genes were activated by IRX1, which may, thus, represent a physiological relationship. On the other hand, the aberrant activation of KLF1 and TAL1 may illustrate an oncogenic function of IRX1 in megakaryoblastic AML. The CBFb-MYH11 fusion gene reportedly deregulates the activity of GATA2 and KLF1, and thus inhibits megakaryopoiesis and erythropoiesis [63]. Here, while excluding the presence of CBFb-MYH11 fusion, we detected the aberrant activity of IRX3/IRX5, an alternative mechanism to perturb myeloid differentiation via KLF1 deregulation.

FLI1 represents an additional myeloid master factor conspicuously downregulated in MEPs, which activates megakaryopoiesis and inhibits erythropoiesis [35,64]. Here, we excluded a regulatory impact of IRX factors in FLI1 expression. However, in our previous study of deregulated NKL homeobox genes, we showed that FLI1 is aberrantly activated by NKX2-3 in megakaryoblastic AML and aberrantly inhibited by NKX2-4 in erythroblastic AML [35]. These observations suggest that MEPs are developmentally susceptible to generate AML and that aberrantly activated homeodomain factors deregulate components of gene regulatory networks, controlling basic myeloid differentiation processes.

Finally, we showed that IRX3 inhibited apoptosis in AML. This effect was not performed by transcriptional activation of BCL2, although BCL2 is physiologically downregulated in MEPs. However, in lung cancer, IRX1 inhibits expression of the pro-apoptotic gene BAX [65], supporting a role of IRX factors in the (de)regulation of cell survival.

Taken together, our study revealed basic impacts of IRX factors in normal and aberrant myelopoiesis. IRX1 is a novel physiological player in hematopoiesis and part of a gene regulatory network controlling the differentiation of megakaryocytes, erythrocytes, and granulocytes. Aberrantly activated IRX1, IRX3, and IRX5 may disturb or deregulate developmental processes in myelopoiesis, driving the generation of AML subsets. Thus, our study contributes to understanding normal and abnormal processes in myelopoiesis.

## 4. Materials and Methods

### 4.1. Bioinformatic Analyses of Expression Profiling and RNA-Seq Data

Expression data for normal cell types were obtained from Gene Expression Omnibus (GEO, www.ncbi.nlm.nih.gov, accessed on 15 February 2022), using expression profiling datasets GSE42519, GSE22552, GSE109348, and GSE24759 [66,67,68,69], in addition to RNA-seq data from The Human Protein Atlas (www.proteinatlas.org, accessed on 15 February 2022) [70]. For screening of cell lines, we exploited RNA-sequencing data from 100 leukemia/lymphoma cell lines (termed LL-100), available at ArrayExpress (www.ebi.ac.uk/arrayexpress, accessed on 15 February 2022) via E-MTAB-7721 [22]. Gene expression profiling data from AML patients were examined using datasets GSE19577, GSE15434, and GSE6891 [71,72,73]. To parse biological functions of 1000 shortlisted genes, gene set annotation enrichment analysis was performed using DAVID bioinformatics resources (www.david.ncifcrf.gov, accessed on 15 February 2022) [74]. Analysis of gene co-expression in profiling datasets was performed by Spearman correlation using R-based tools.

### 4.2. Cell Lines and Treatments

Cell lines are held at the DSMZ (Braunschweig, Germany) and cultivated as described (www.DSMZ.de, accessed on 15 February 2022). All cell lines had been authenticated and tested negative for mycoplasma infection. Modification of gene expression levels was performed using gene-specific siRNA oligonucleotides with reference to AllStars negative Control siRNA (siCTR) obtained from Qiagen (Hilden, Germany). SiRNAs (80 pmol) were transfected into 1 × 10^6^ cells by electroporation using the EPI-2500 impulse generator (Fischer, Heidelberg, Germany) at 350 V for 10 ms. Electroporated cells were harvested after 20 h of cultivation. Cell treatments were performed for 20 h using 20 ng/mL of recombinant BMP2 (R & D Systems, Wiesbaden, Germany, #355-BM-010/CF) or 20 µg/mL of inhibitory monoclonal anti-BMP2 antibody (R & D Systems, #MAB3552).

For cytological analyses, cell lines were stained with Giemsa–May–Grünwald as follows: Cells were spun onto microscope slides and fixed for 5 min with methanol. Subsequently, they were stained for 3 min with May–Grünwald’s eosin-methylene blue modified solution (Merck, Darmstadt, Germany) diluted in Titrisol (Merck), and for 15 min with Giemsa’s azur eosin methylene blue solution (Merck). Images were captured with an Axion A1 microscope using Axiocam 208 color and software ZEN 3.3 blue edition (Zeiss, Göttingen, Germany). For functional testing, treated cells were analyzed with the IncuCyte S3 Live-Cell Analysis System (Essen Bioscience, Hertfordshire, UK). For detection of apoptotic cells, we additionally used the IncuCyte Caspase-3/7 Green Apoptosis Assay diluted at 1:2000 (Essen Bioscience). Live-cell imaging experiments were performed twice with fourfold parallel tests.

### 4.3. Polymerase-Chain-Reaction (PCR) Analyses

Total RNA was extracted from cultivated cell lines using TRIzol reagent (Invitrogen, Darmstadt, Germany). Primary human total RNA derived from the cerebellum, kidney, lung, and salivary gland was purchased from Biochain/BioCat (Heidelberg, Germany). cDNA was synthesized using 1 µg of RNA, random priming, and Superscript II (Invitrogen). Real-time quantitative (RQ)-PCR analysis was performed using the 7500 Real-time System and commercial buffer and primer sets (Applied Biosystems/Life Technologies, Darmstadt, Germany). For normalization of expression levels, we quantified the transcripts of TATA box binding protein (TBP). Quantitative analyses were performed as biological replicates and measured in triplicate. Standard deviations are presented in the figures as error bars. Statistical significance was assessed by Student’s *t*-test (two-tailed) and the calculated p-values indicated by asterisks (* *p* < 0.05, ** *p* < 0.01, *** *p* < 0.001, n.s. not significant).

For detection of CBFb-MYH11 and SET-NUP214 fusion transcripts, we performed reverse transcription (RT)-PCR, using the following oligonucleotides as reported previously: CBFb-for 5′-GGGCTGTCTGGAGTTTGATG-3′, and MYH11-rev 5′-CTTGAGCGCCTGCATGTT-3′, SET-for 5′-TGACGAAGAAGGGGATGAGGAT-3′, NUP214-rev 5′-ATCATTCACATCTTGGACAGCA-3′ [28,44]. As a control, we analyzed ETV6, using ETV6-for 5′-AGGCCAATTGACAGCAACAC-3′ and ETV6-rev 5′-TGCACATTATCCACGGATGG-3′. All oligonucleotides were purchased from Eurofins MWG (Ebersberg, Germany). PCR products were generated using taqpol (Qiagen) and thermocycler TGradient (Biometra, Göttingen, Germany), analyzed by gel electrophoresis, and documented with the Azure c200 Gel Imaging System (Azure Biosystems, Dublin, CA, USA).

### 4.4. Protein Analysis

Western blots were generated by the semi-dry method. Protein lysates from cell lines were prepared using SIGMAFast protease inhibitor cocktail (Sigma, Taufkirchen, Germany). Proteins were transferred onto nitrocellulose membranes (Bio-Rad, München, Germany) and blocked with 5% dry milk powder dissolved in phosphate-buffered-saline buffer (PBS). The following antibodies were used: alpha-Tubulin (Sigma, #T6199), IRX1 (Biozol, Eching, Germany, #DF3225), and IRX3 (Biozol, #MBS8223417). For loading control, blots were reversibly stained with Poinceau (Sigma) and the detection of alpha-Tubulin (TUBA) was performed thereafter. Secondary antibodies were linked to peroxidase for detection by Western-Lightning-ECL (Perkin Elmer, Waltham, MA, USA). Documentation was performed using the digital system ChemoStar Imager (INTAS, Göttingen, Germany).

The enzyme-linked immunosorbent assay (ELISA) was used to quantify BMP2 protein levels in the supernatant of cell cultures. In addition, 2 × 10^6^ cells were cultured in 2 mL of fresh medium in a 24-well plate. After 24 h, 1 of mL medium was harvested and frozen in aliquots. Quantification was performed using the Quantikine ELISA BMP-2 kit (R & D Systems, #DBP200), as described by the company. Two biological replicates were analyzed in triplicate.

### 4.5. Karyotyping and Genomic Profiling Analysis

Karyotyping was performed as described previously [75]. For genomic profiling, genomic DNA of AML cell lines was prepared by the Qiagen Gentra Puregene Kit (Qiagen). Labeling, hybridization, and scanning of Cytoscan HD arrays were performed by the Genome Analytics Facility located at the Helmholtz Centre for Infection Research (Braunschweig, Germany), using the manufacturer´s protocols (Affymetrix, High Wycombe, UK). Data were interpreted using the Chromosome Analysis Suite software version 3.1.0.15 (Affymetrix, High Wycombe, UK) and copy number alterations were determined accordingly.

## Data Availability

Data is contained within the article or Supplementary Materials.

## Supplementary Materials

The following supporting information can be downloaded at: https://www.mdpi.com/article/10.3390/ijms23063192/s1.
