# Supplementary material for: The Hematopoietic TALE-Code Shows Normal Activity of IRX1 in Myeloid Progenitors and Reveals Ectopic Expression of IRX3 and IRX5 in Acute Myeloid Leukemia
Source: Int J Mol Sci. 2022 Mar 16;23(6):3192. doi: 10.3390/ijms23063192 (PMC8952210; doi:10.3390/ijms23063192)
Supplement: Supplementary file 1 [file ijms-23-03192-s001.zip › Supplementary Figures.pdf]

Myelopoiesis dataset GSE42519 (cutoff: 5)

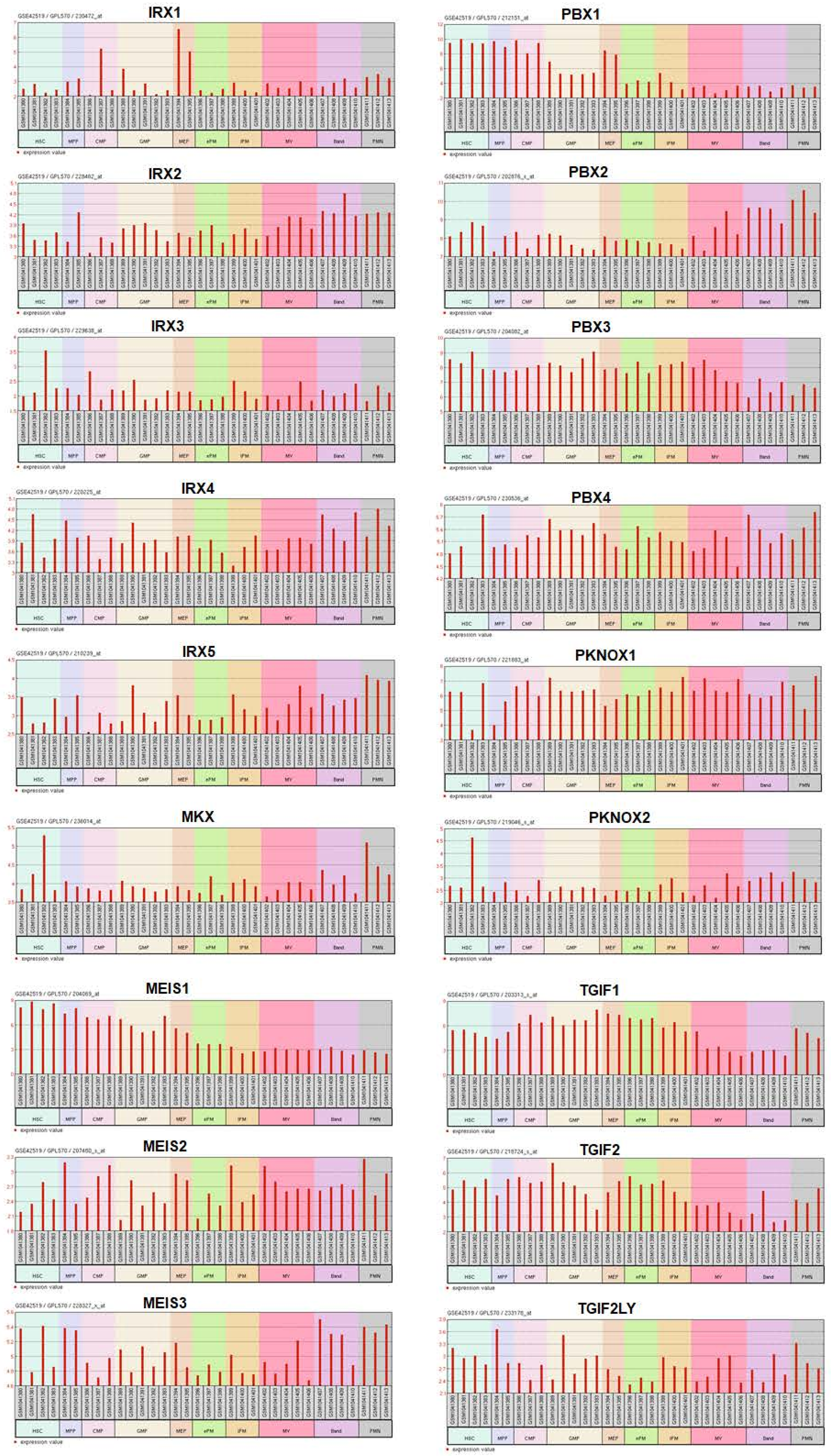

Abbreviations:
HSC: hematopoietic stem cell
MPP: myeloid primed progenitor
CMP: common myeloid progenitor
GMP: granulo-myeloid progenitor
MEP: megakaryocytic-erythroid progenitor
e PM: early promyelocyte
l PM: late promyelocyte
MY: myelocyte
Band: band cells
PMN: polymorphonuclear cells

Myelopoiesis dataset GSE22552 (cutoff: 7)

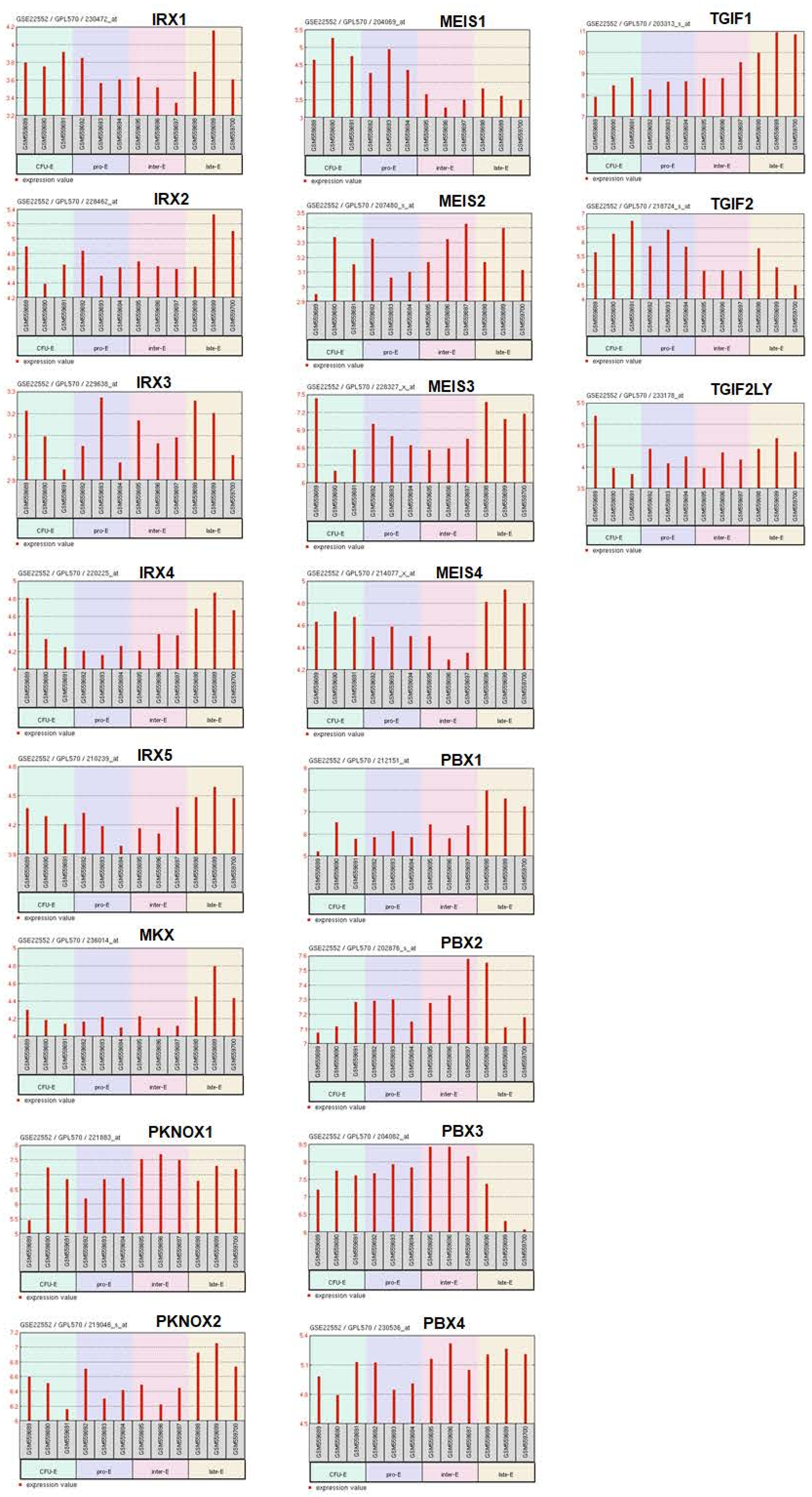

Abbreviations:
CFU-E: colony-forming unit erythroblasts
Pro-E: pro-erythroblast
inter-E: intermediate erythroblast
late-E: late erythroblast

Myelopoiesis dataset GSE109348 (cutoff: 7)

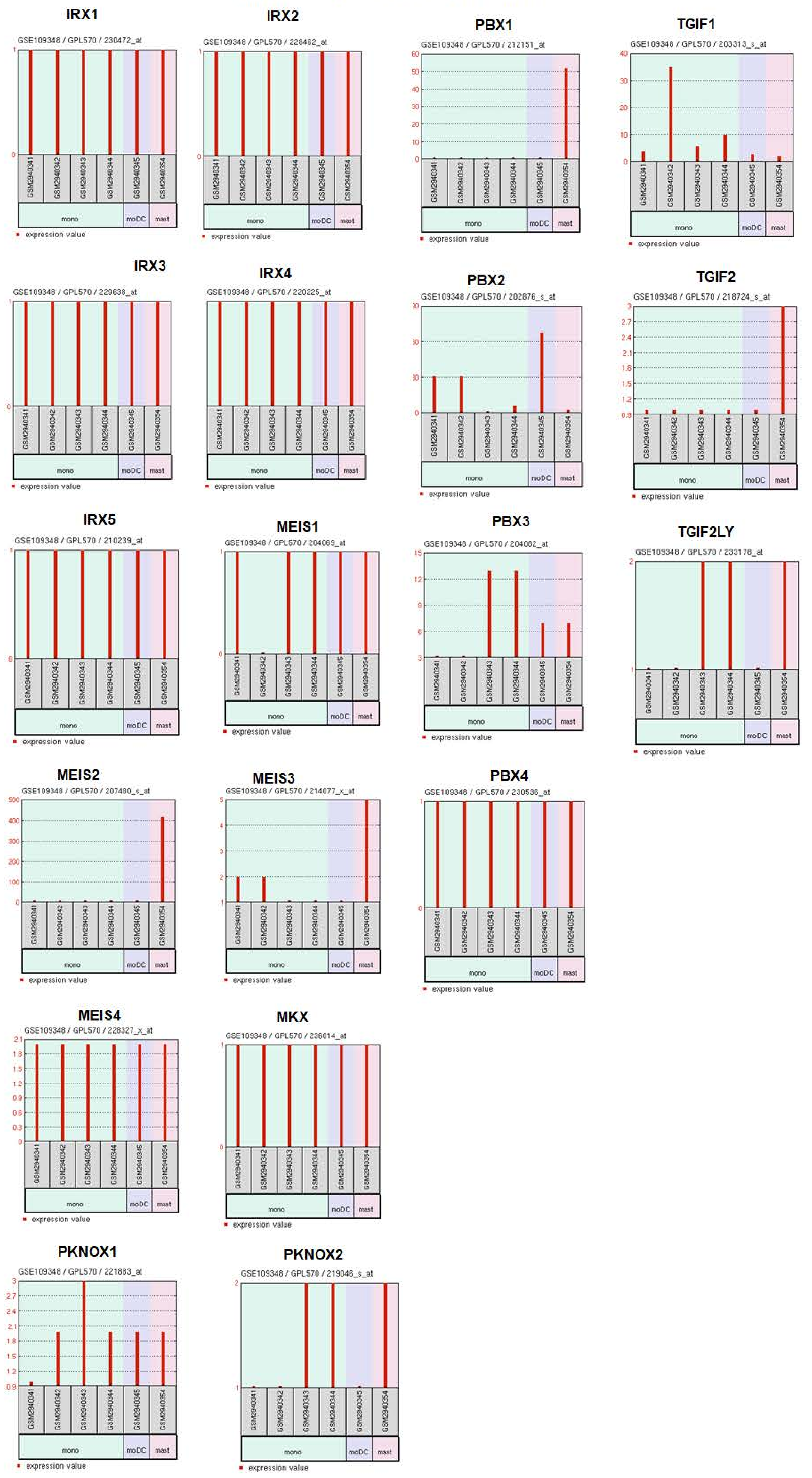

Abbreviations:
mono: monocyte
moDC: monocyte-derived dendritic cell
mast: mast cell

Myelopoiesis dataset GSE24759 (cutoff: 7)

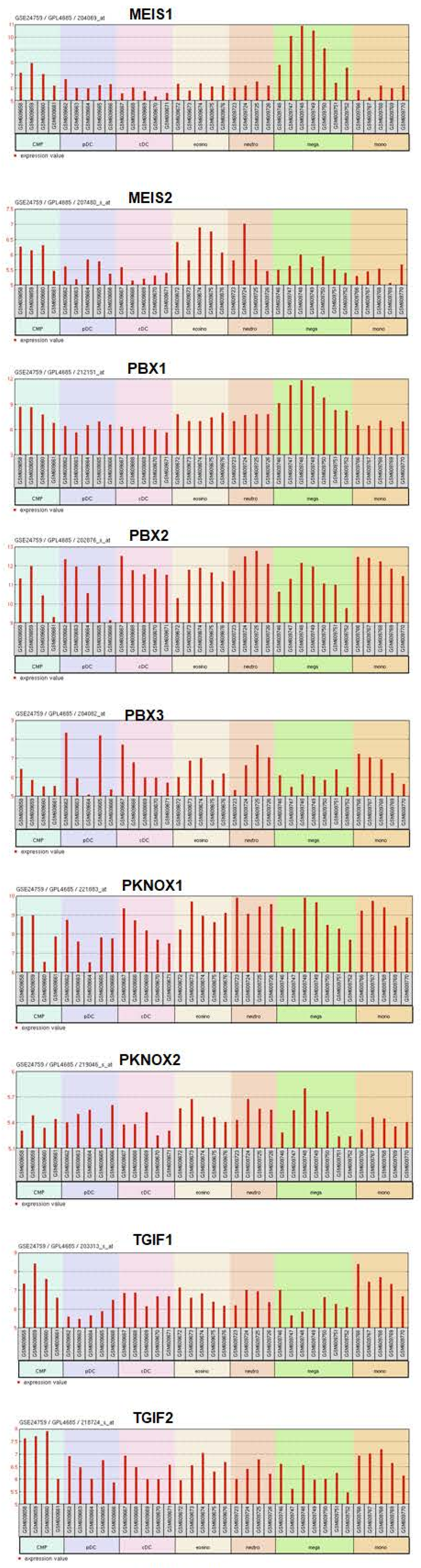

Abbreviations:
CMP: common myeloid progenitor
pDC: plasmocytoid dendritic cell
cDC: conventional dendritic cell
eosino: eosinophils
neutro: neutrophils
mega: megakaryocytes
mono: monocytes

The Human Proteome Atlas

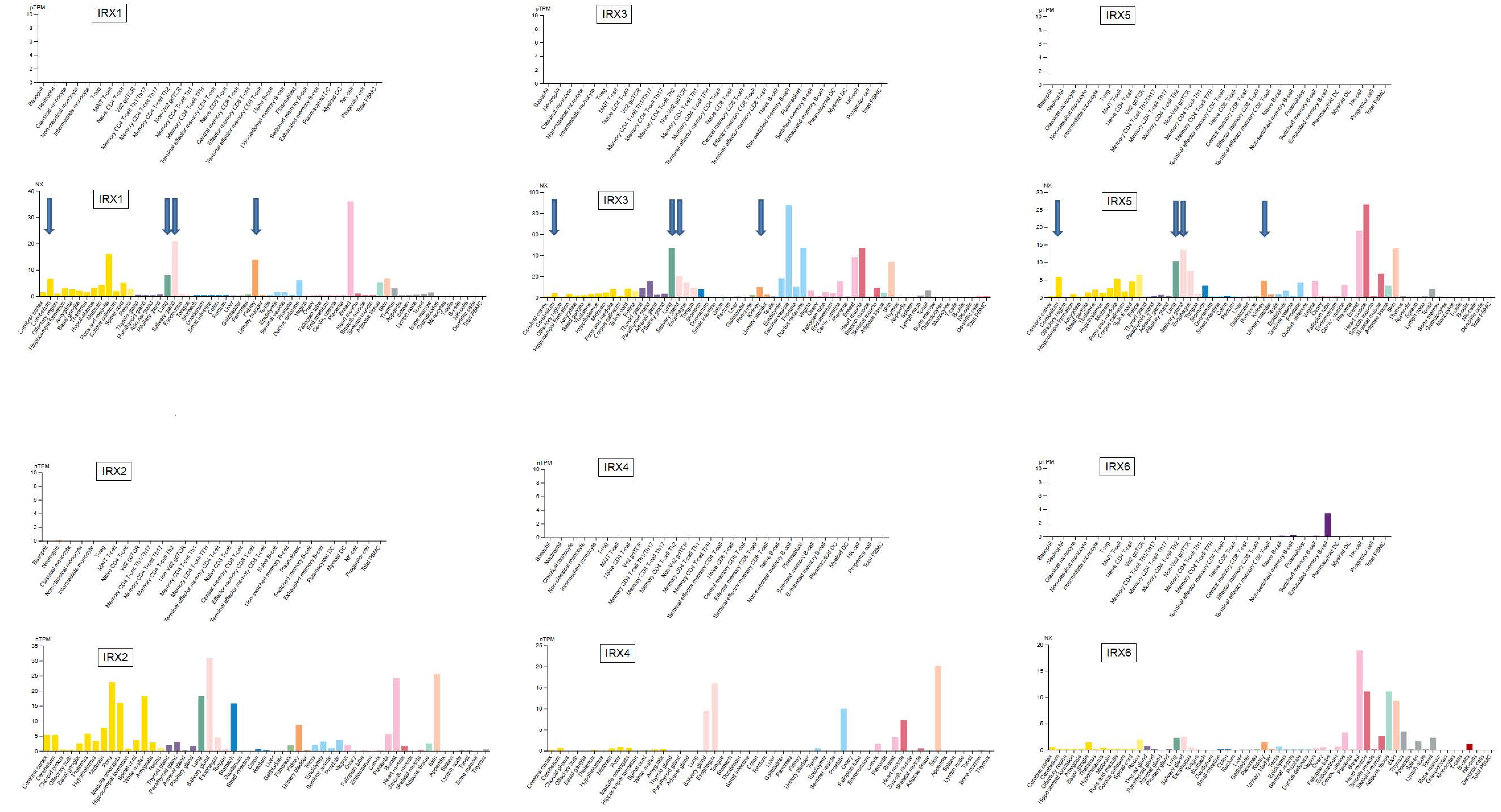

RNA-seq expression data for IRX1, IRX2, IRX3, IRX4, IRX5 and IRX6 were obtained from the Human Protein Atlas. For each gene, activities are shown in entities belonging to the hematopoietic (above) or alternative tissue compartment (below). Blue arrows indicate selected tissues which were analyzed in this study.

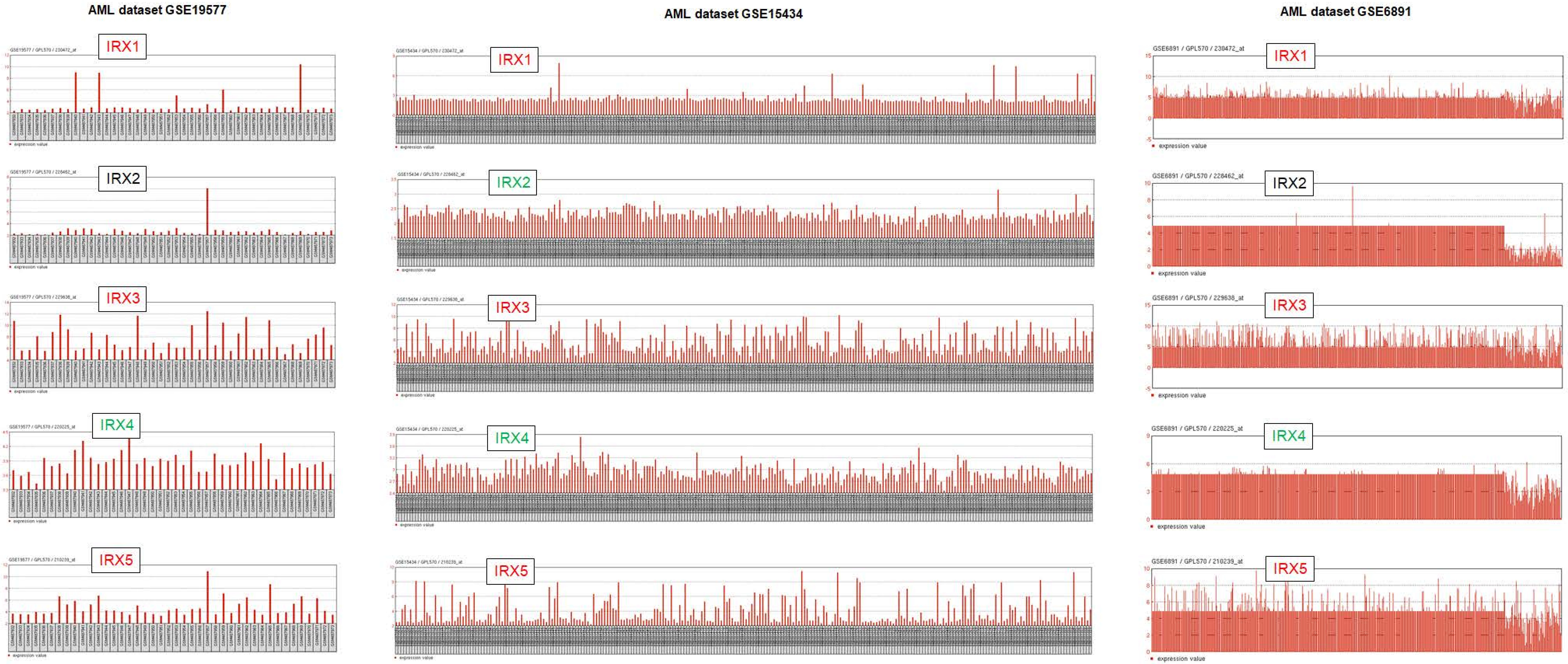
AML dataset GSE19577
AML dataset GSE15434
AML dataset GSE6891
IRX1
IRX2
IRX3
IRX4
IRX5
GSE6891 / GPL570 / 230472_at
GSE6891 / GPL570 / 228462_at
GSE6891 / GPL570 / 229638_at
GSE6891 / GPL570 / 220225_at
GSE6891 / GPL570 / 210239_at
expression value

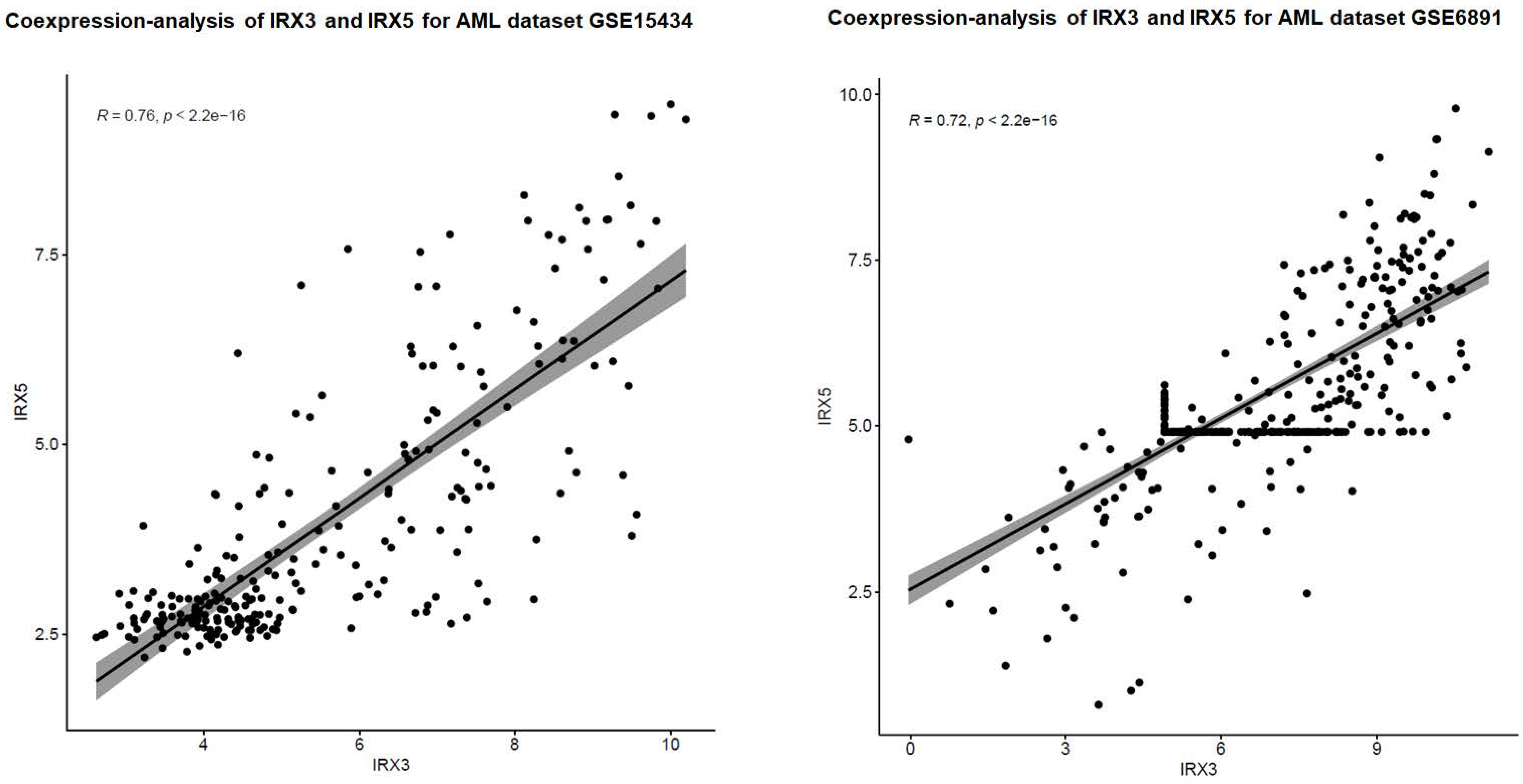

Coexpression-analysis of IRX3 and IRX5 for AML dataset GSE15434
R = 0.76, p < 2.2e−16
IRX5
IRX3
7.5
5.0
2.5
4
6
8
10
Coexpression-analysis of IRX3 and IRX5 for AML dataset GSE6891
R = 0.72, p < 2.2e−16
IRX5
IRX3
10.0
7.5
5.0
2.5
0
3
6
9

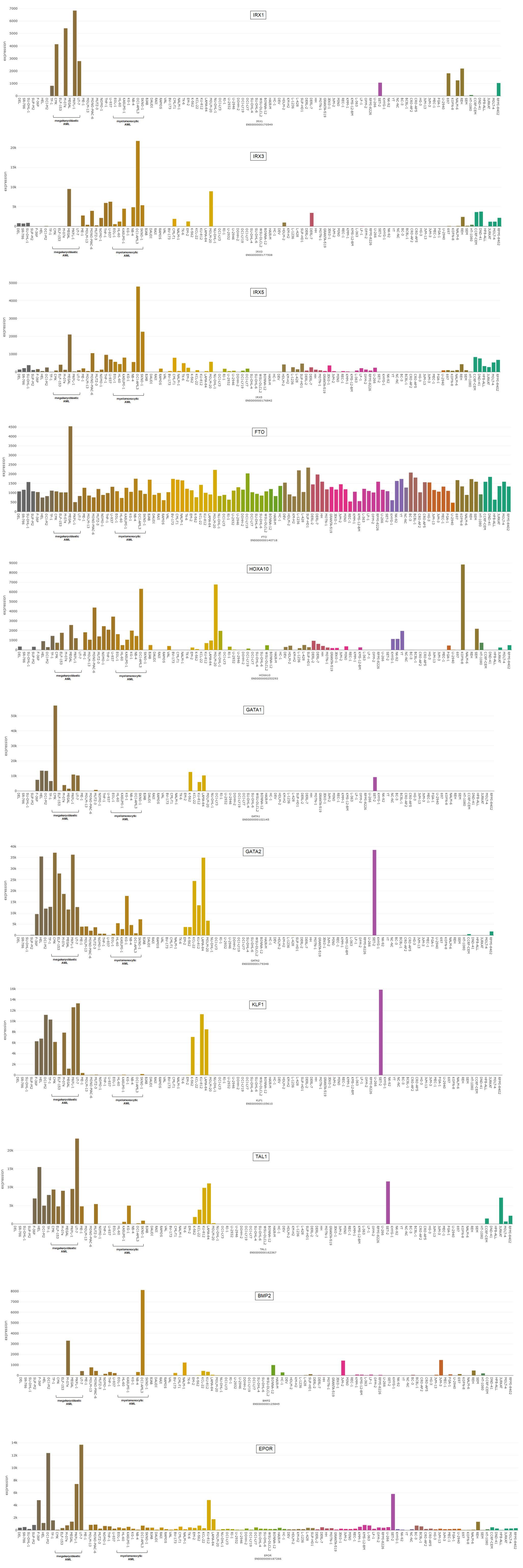

RNA-seq expression data for selected genes were obtained from the LL-100 dataset. Megakaryoblastic and myelomonocytic AML entities are indicated.

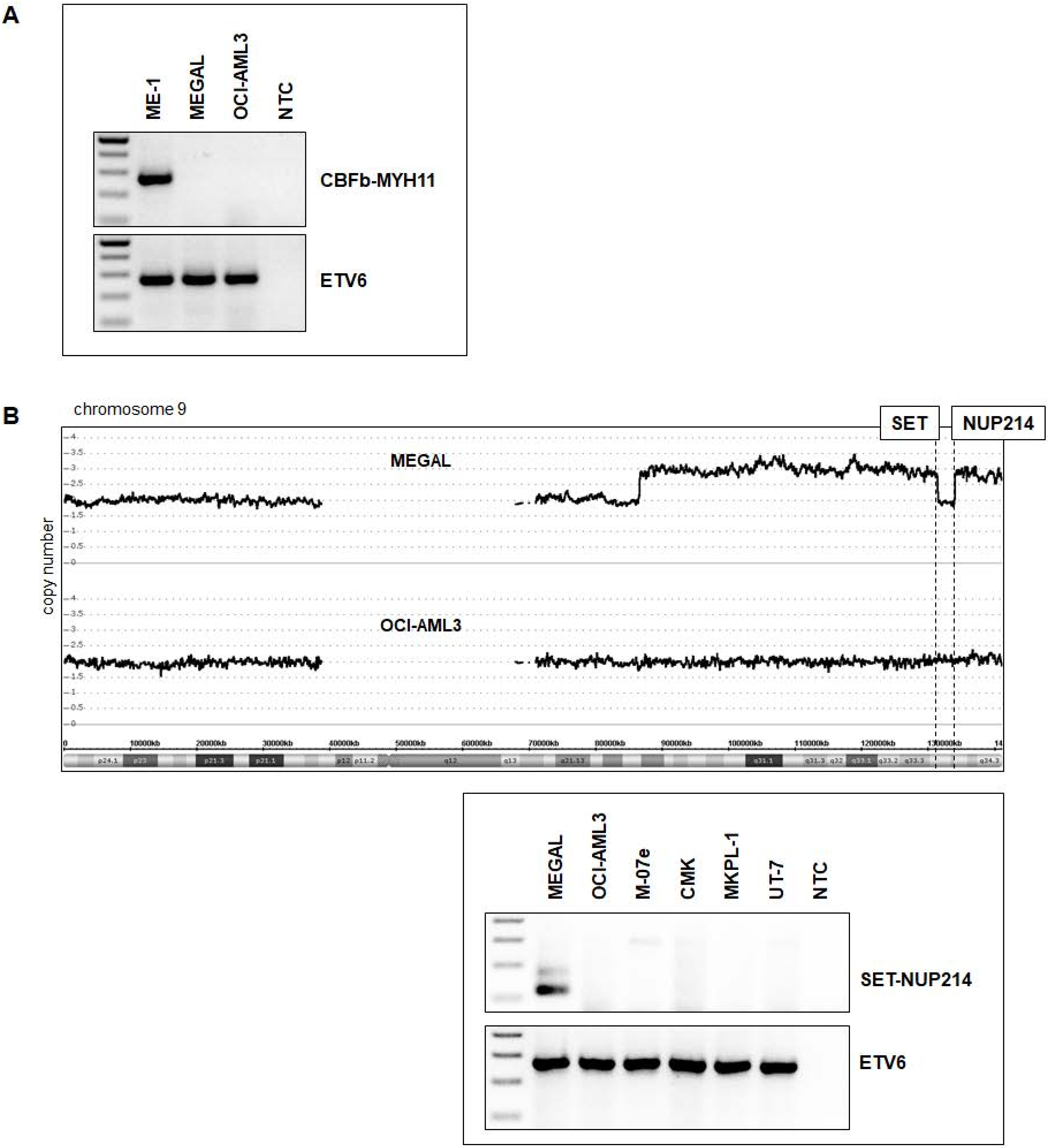

(A) RQ-PCR analysis of fusion gene CBFb-MYH11. AML cell line ME-1 served as positive control for the fusion transcript. ETV6 served as control for cDNA integrity. (B) Genomic profiling data for chromosome 9 from cell lines MEGAL and OCI-AML3 show a deletion in MEGAL at 9q34 which targets the genes SET and NUP214 (above). RQ-PCR analysis of fusion gene SET-NUP214 confirmed the presence in cell line MEGAL (below). ETV6 served as control for cDNA integrity. NTC: no template control.

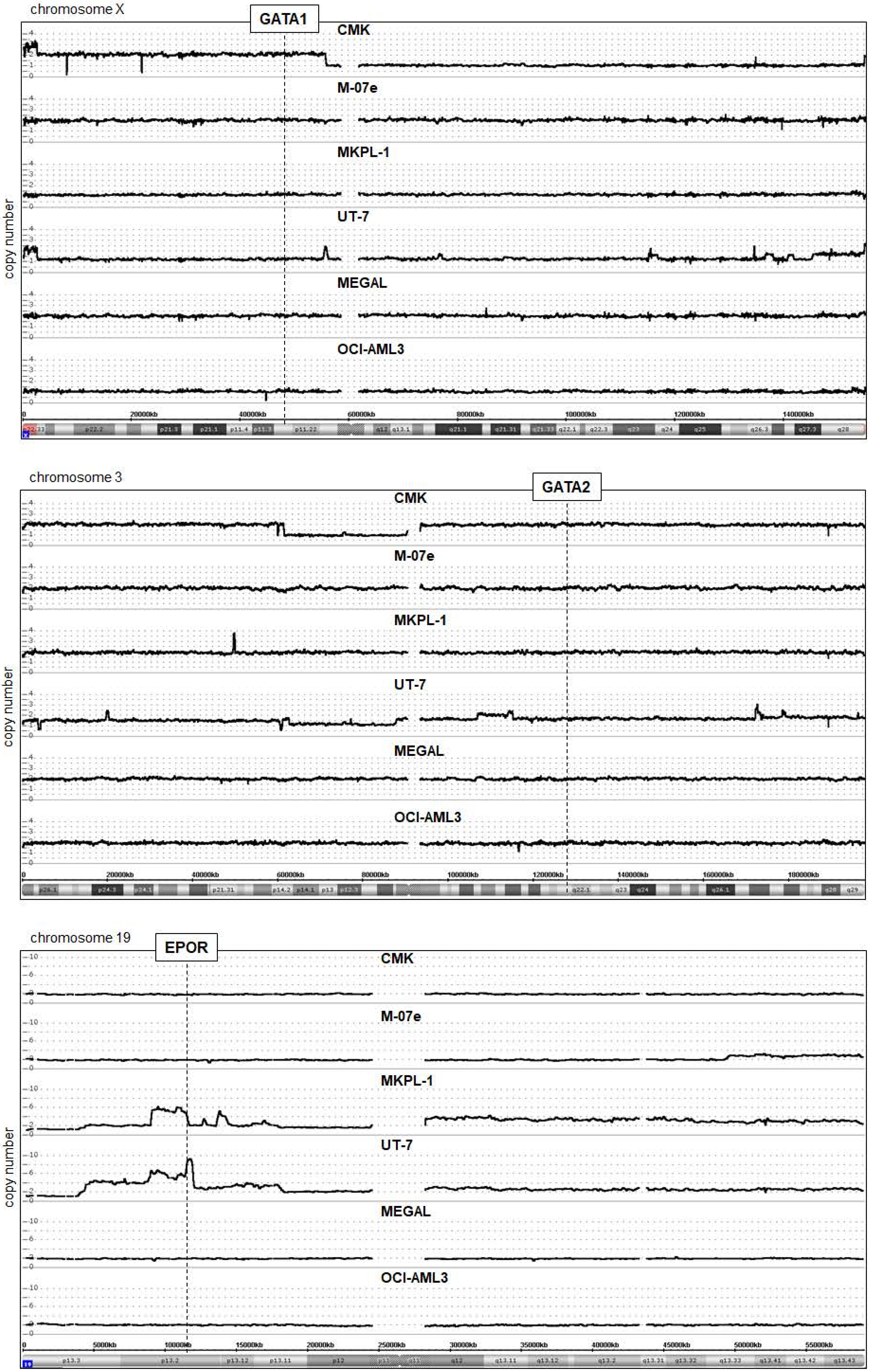

Genomic profiling data for chromosome X (above), chromosome 3 (middle), and chromosome 19 (below) from cell lines CMK, M-07e, MKPL-1, UT-7, MEGAL and OCI-AML3. The genes GATA1, GATA2 and EPOR are indicated.

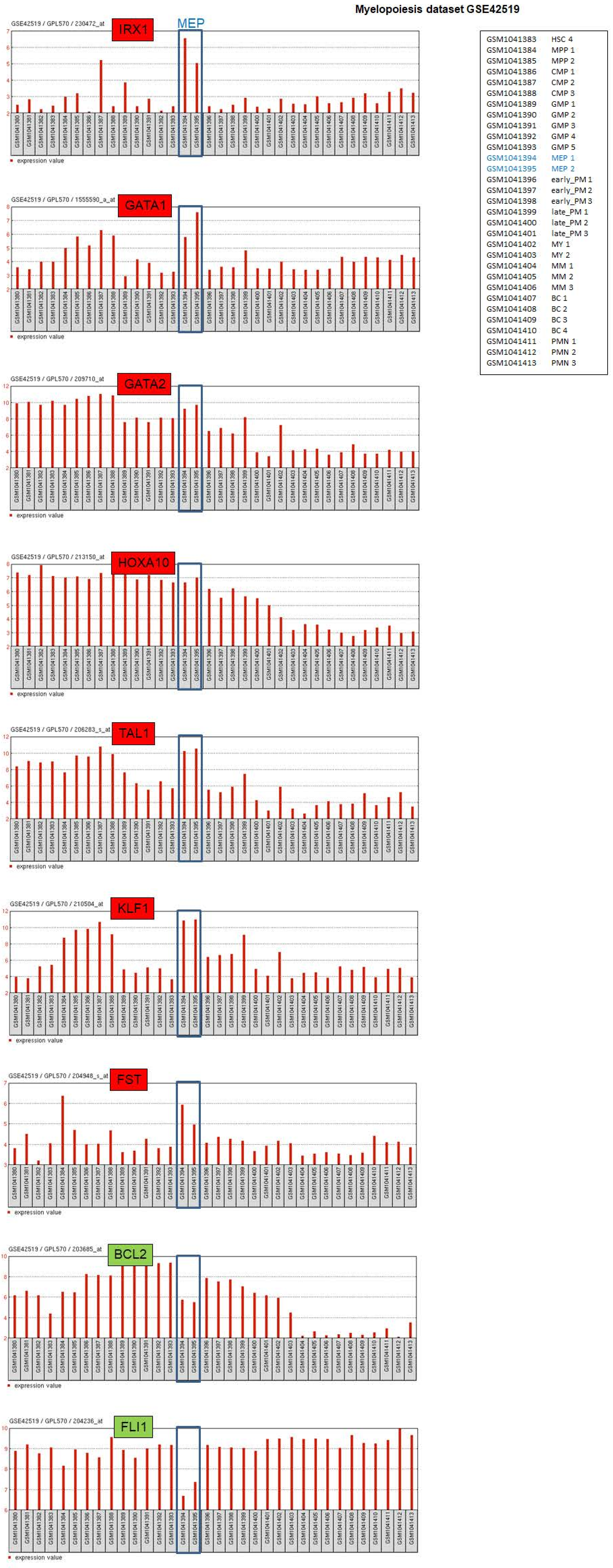

Gene expression profiling data for selected genes from dataset GSE42519. The data for MEP are highlighted in blue. Genes elevated or decreased in MEP are indicated in red or green, respectively.
