# Supplementary material for: The Hematopoietic TALE-Code Shows Normal Activity of IRX1 in Myeloid Progenitors and Reveals Ectopic Expression of IRX3 and IRX5 in Acute Myeloid Leukemia
Source: Int J Mol Sci. 2022 Mar 16;23(6):3192. doi: 10.3390/ijms23063192 (PMC8952210; doi:10.3390/ijms23063192)
Supplement: Supplementary file 1 [file ijms-23-03192-s001.zip › Table S1_LL100_IRX1-IRX35.pdf]

gene	CMK	M-07e	MKPL-1	UT-7	OCI-AML3	MEGAL	IRX1	IRX35	IRX1-IRX35
EEF1A1	1242567	1249235	1354612,5	1298236	1288572,7	566886,8	1286162,78	927729,75	358433,025
HBG2	118671,9	65203,95	86844,53	407387,5	0	15,79	169526,975	7,895	169519,08
AC104389.6	72407,63	29972,95	377741,35	87502,85	0	0	141906,195	0	141906,195
HBG1	69851,76	24291,81	1253,87	374225	0	12,23	117405,608	6,115	117399,493
GLUL	157283,8	71999,53	81074,42	219373,6	25248,95	11287,73	132432,82	18268,34	114164,48
TFRC	108932,9	130618,7	128238,09	219380	6135,98	95318,19	146792,39	50727,085	96065,305
RPL13A	232972,1	391593	429906,39	227075,2	373496,32	81163,6	320386,663	227329,96	93056,7025
HSP90AB1	205847,2	147852,2	184058,83	279946,2	163180,86	86911,45	204426,09	125046,155	79379,935
ITGA2B	79497,21	42930,7	196079,73	65428,02	146,89	33203,03	95983,915	16674,96	79308,955
MT-CO2	196349,8	273481,8	274326,73	205766,7	218407,94	111935	237481,268	165171,47	72309,7975
APOE	16218,59	32464,04	182151,04	49259,19	63,62	167,38	70023,215	115,5	69907,715
HSPA8	219859	132433,5	99208,35	203825,3	156784,79	38700,84	163831,535	97742,815	66088,72
PRKCSH	16022,72	30297,46	95515,63	199618,6	25604,1	13145,74	85363,59	19374,92	65988,67
MT-CO3	271710,3	347480,3	431733,99	244012,8	276963,81	238629,5	323734,328	257796,67	65937,6575
FTH1	89488,45	83027,51	137803,75	136921,4	57066,16	40896,52	111810,265	48981,34	62828,925
CALR	111091,9	54179,64	144562,86	111848,8	38256,67	47246,64	105420,798	42751,655	62669,1425
RPL10	140681,3	136161,3	180251,07	185920,7	146644,57	51525,96	160753,58	99085,265	61668,315
RPLP0	264458,2	227220,4	295495,68	197442,7	280271,59	90236,9	246154,248	185254,245	60900,0025
TUBB	174797	148273,4	136476,62	155884,2	116576,75	70675,76	153857,81	93626,255	60231,555
PIM1	35678,52	38502,64	96378,52	78794,68	377,59	6132,4	62338,59	3254,995	59083,595
HNRNPA1	220575,9	236493,8	201587,34	182616,7	200623,89	102000,8	210318,43	151312,355	59006,075
ENO1	230771,4	240135,4	307260,94	183592,4	280222	83868,13	240440,023	182045,065	58394,9575
HSPG2	14033,29	84077,84	73989,57	59334,01	284,88	3752,76	57858,6775	2018,82	55839,8575
RPL4	202597,9	215386,6	222625,35	181304,8	202598,45	96851,86	205478,633	149725,155	55753,4775
TRIM28	74198,71	106776	117944,76	64396,93	38188,46	32513,63	90829,0975	35351,045	55478,0525
RPS3	238253,3	213625	187608,96	226281,8	252866,21	69798,08	216442,24	161332,145	55110,095
HDC	75619,23	136,35	127333,81	21647,76	3,46	2279,45	56184,2875	1141,455	55042,8325
ILF3	45417,19	50150,8	137178,27	96771,67	24359,43	31307,82	82379,4825	27833,625	54545,8575
FLNA	90548,87	95819,78	141844,61	205478,3	88759,27	73258,41	133422,9	81008,84	52414,06
PRG2	43747,52	212,22	48462	114295,5	97,98	36,91	51679,305	67,445	51611,86
RPS5	89819,5	132589,8	132361,05	97871,24	97958,37	31806,96	113160,395	64882,665	48277,73
RPS2	225886,3	269593,2	215286,96	195857	319802,6	39526,63	226655,875	179664,615	46991,26

**SMARCA4**	24918,64	27448,5	126775,18	83664,47	20899,15	19516,21	65701,6975	20207,68	45494,0175
**PFN1**	63394,24	115831,8	181891,06	109634,3	118553,9	27226,25	117687,845	72890,075	44797,77
**RPL18A**	111494,2	110070,4	97323,12	153943,7	113978,15	37365,6	118207,843	75671,875	42535,9675
**TMSB10**	31958,01	43037,02	123776,57	49401,69	26653,75	13662,01	62043,3225	20157,88	41885,4425
**RPS9**	69282,42	127262,8	113419,84	81486,81	91530,72	23212,64	97862,9725	57371,68	40491,2925
**TPM4**	16179,8	42734,7	122527,82	66341,37	14789,22	29080,31	61945,9225	21934,765	40011,1575
**DNM2**	12523,11	16826,02	73580,5	90545,69	8957,36	10333,42	48368,83	9645,39	38723,44
**RPS11**	104901	118616,6	224011,58	109880,2	156021,06	47210,49	139352,345	101615,775	37736,57
**MT-ND4**	309245	322753,5	331251,5	242542	269242,12	258649,4	301448,008	263945,75	37502,2575
**TUBA1B**	201715,7	139181,5	151785,8	163833,4	95578,15	157987,6	164129,09	126782,88	37346,21
**RHAG**	25752,99	60821,85	40327,08	25716,4	0	2437,04	38154,58	1218,52	36936,06
**RPS19**	68767,31	84424,05	164305,56	73846,17	96710,73	25278,85	97835,7725	60994,79	36840,9825
**HMGA1**	91697,09	56749,39	48432,71	30421,53	26888,02	13251,8	56825,18	20069,91	36755,27
**LAPTM5**	31841,33	77935,39	63685,44	82007,56	30799,15	24285,79	63867,43	27542,47	36324,96
**HSPA5**	76541,85	55442,15	47854,57	48267,7	24442,77	18276,53	57026,5675	21359,65	35666,9175
**MT-CO1**	574923,3	690272,3	656997,68	536111,7	621536,7	536703,4	614576,245	579120,07	35456,175
**GATA1**	101163,4	6953,14	19903,01	18229,44	0	2900,42	36562,255	1450,21	35112,045
**CSF2RB**	51280,5	16705,99	27236,16	47957,61	2009,29	524,33	35795,065	1266,81	34528,255
**RPL3**	179979,1	249469,5	202614,67	170118,9	253299,09	79551,79	200545,513	166425,44	34120,0725
**KCNH2**	28376,84	37177,36	36199,08	33722,64	6,68	22,97	33868,98	14,825	33854,155
**RPL6**	121032,6	117322,1	135696,07	103902,9	125329,32	46628,69	119488,415	85979,005	33509,41
**PSAP**	48981,65	33175,69	119928,85	33502,97	18899,99	31962,4	58897,29	25431,195	33466,095
**IGFBP4**	527,71	42607,8	65130,87	98064,03	4509,44	32060,07	51582,6025	18284,755	33297,8475
**RPL18**	70665,38	80509,89	143438,1	96674,96	99281,49	31121,94	97822,0825	65201,715	32620,3675
**GATA2**	63632,83	33068,4	55733,55	20722,73	1433,09	20790,99	43289,3775	11112,04	32177,3375
**SH3BGRL3**	30363,91	23767,57	71523,55	35170,4	9617,2	8069,22	40206,3575	8843,21	31363,1475
**CFL1**	103384,8	56518,38	92429,34	62656,08	51111,13	43728,68	78747,155	47419,905	31327,25
**SLC40A1**	35655,71	35119,13	25376,15	52388,93	339,33	11505,07	37134,98	5922,2	31212,78
**TLN1**	70856,72	80775,51	62953,53	55941,1	23342,13	49922,49	67631,715	36632,31	30999,405
**APOC1**	7765,03	11647,45	80613,42	25088,82	317,68	483,77	31278,68	400,725	30877,955
**ACTN4**	28897,3	41985,62	64216,62	71852,89	13847,55	28363,82	51738,1075	21105,685	30632,4225
**YBX1**	80924,34	77228,36	95990,03	103672,7	77425,07	40820,37	89453,8525	59122,72	30331,1325
**RPL11**	64583,21	73066,58	146090,59	68365,5	84821,49	34248,16	88026,47	59534,825	28491,645

**EIF5A**	31230,49	59159,48	69393,95	42494,61	30237,7	13950,01	50569,6325	22093,855	28475,7775
**DIAPH1**	49917,71	79340,42	27498,02	36223,76	22028,24	17938,78	48244,9775	19983,51	28261,4675
**DLK1**	111781,6	1099,41	84,6	0	0	1,93	28241,4075	0,965	28240,4425
**SPINT2**	10676,72	17633,86	82664	17612,22	0,94	8004,67	32146,7	4002,805	28143,895
**RPL28**	60377,75	91813,3	136304,15	77546,92	102727,03	24236,93	91510,53	63481,98	28028,55
**ESYT1**	24283,94	40920,96	33381,6	48775,64	7139,83	11934,94	36840,535	9537,385	27303,15
**GPI**	53332,94	50232,55	87114,11	52834,97	50020,09	17169,08	60878,6425	33594,585	27284,0575
**SMARCA2**	15128,05	13107,48	14400,58	99257,68	5234,53	11224,21	35473,4475	8229,37	27244,0775
**EGFL7**	19646,3	18007,89	45900,3	29773,1	721,43	1471,4	28331,8975	1096,415	27235,4825
**VASP**	27756,28	20084,87	46970,67	40111,45	8629,39	4559,84	33730,8175	6594,615	27136,2025
**CDC37**	11930,71	16514,68	64299,37	60456,04	15060,99	7738,37	38300,2	11399,68	26900,52
**PDIA3**	62986,66	35271,34	70396,56	38699,06	27372,2	22531,15	51838,405	24951,675	26886,73
**MYC**	27139,61	63974,61	27919,01	45278,91	27626,4	1087,96	41078,035	14357,18	26720,855
**MLC1**	12339,83	60981,61	24513,36	21445,69	619,46	7099,26	29820,1225	3859,36	25960,7625
**SLC25A6**	77768,26	75522,99	21514,38	51724,41	38647,31	23454,8	56632,51	31051,055	25581,455
**RPSA**	134167,1	124696,9	105810,75	77459,93	118156,16	53319,35	110533,683	85737,755	24795,9275
**PFKL**	22593,52	24221,69	66378,97	52030,42	23811,05	9255,39	41306,15	16533,22	24772,93
**S100A6**	11391,78	11298,45	59139,55	44283,7	8896,07	4845,68	31528,37	6870,875	24657,495
**PIEZO1**	48426,4	51309,47	113502,12	45935,39	37200,42	43146,41	64793,345	40173,415	24619,93
**CD24**	81429,26	52,19	8647,31	8079,51	0	2,48	24552,0675	1,24	24550,8275
**MT-ND1**	217787,2	177887,9	146483,85	86701,39	146088,88	120189,9	157215,088	133139,38	24075,7075
**TAL1**	17601,46	16839,76	18559	43253,53	291,04	13,17	24063,4375	152,105	23911,3325
**LDB1**	29899,9	36067,6	32371,6	33323,87	4038,19	14131,6	32915,7425	9084,895	23830,8475
**EIF3G**	14605,21	12640,99	64271,84	48012,43	15024,2	7240,69	34882,6175	11132,445	23750,1725
**SOD1**	32395,81	11517,77	81012,13	22734,79	14458,51	12336,48	36915,125	13397,495	23517,63
**TCP1**	41178,1	38173,97	22780,34	68619,74	18714,34	19897,72	42688,0375	19306,03	23382,0075
**MT-ATP6**	181610,1	226052,2	236851,05	172388,8	149322,81	212443,2	204225,563	180883,005	23342,5575
**HDAC7**	17722,94	11798,04	38145,86	35790,6	2116,82	3362,34	25864,36	2739,58	23124,78
**RPS28**	33264,47	30241,78	74989,03	61428,3	46567,74	7384,56	49980,895	26976,15	23004,745
**HBA1**	15110,78	585,83	57665,27	18054,93	0	9,09	22854,2025	4,545	22849,6575
**TGM2**	4636,96	26690,15	30891,66	29275,3	108,64	5,04	22873,5175	56,84	22816,6775
**NFE2**	26432,44	3563,05	53254,46	23351,5	891,38	6889,94	26650,3625	3890,66	22759,7025
**ANK1**	17452,57	23981,99	21559,17	25796,18	0	3,14	22197,4775	1,57	22195,9075

**RPS16**	74829,22	77032,25	139331,86	82632,12	105424,89	37442,62	93456,3625	71433,755	22022,6075
**SLC1A5**	25063,43	26556,94	47302,28	53879,71	12854,82	20642,78	38200,59	16748,8	21451,79
**DDOST**	28348,82	25285,84	60037	34476,27	19249,23	11943,28	37036,9825	15596,255	21440,7275
**DNMT1**	26004,39	22155,69	57057,46	70389,59	16978,9	28181,48	43901,7825	22580,19	21321,5925
**S100A4**	24759,48	21659,79	46488,94	25544,68	11940,52	5255,21	29613,2225	8597,865	21015,3575
**HK1**	27131,58	20561,97	23267,8	39053,39	4807,81	8728,83	27503,685	6768,32	20735,365
**UBB**	70037,14	59941,82	100448,08	56486,82	50918,1	51850,9	71728,465	51384,5	20343,965
**HNRNPAB**	40326,65	36247,18	26154,28	44039,88	14188,79	18673,19	36691,9975	16430,99	20261,0075
**LY6E**	25349,17	29714,25	36615,82	10507,59	10703,22	13,01	25546,7075	5358,115	20188,5925
**RPS10**	70102,34	66168,7	115889,15	63320,02	103114,23	14362,82	78870,0525	58738,525	20131,5275
**KHSRP**	28776,93	41122,25	24754,03	69737,96	13824,1	28140,85	41097,7925	20982,475	20115,3175
**PTMA**	159778,7	191655,3	192607,52	148762,9	210705,89	95591,19	173201,1	153148,54	20052,56
**CHD4**	70303,72	54583,37	69747,51	38530,54	37943,01	38762,6	58291,285	38352,805	19938,48
**RAVER1**	13113,41	14835,34	37401,75	43433,56	3504,81	11058,87	27196,015	7281,84	19914,175
**HSPD1**	92252,04	64603,26	38484,94	64338,53	73071,71	17450,24	64919,6925	45260,975	19658,7175
**EIF4G2**	74632,19	54096,12	44531,27	54405,57	42680,39	32178,27	56916,2875	37429,33	19486,9575
**MYH9**	34719,76	117123,8	41181,62	56161,48	50382,51	35251,41	62296,6625	42816,96	19479,7025
**CD34**	88076,85	102,17	46247,45	382,74	5,65	28528,62	33702,3025	14267,135	19435,1675
**U2AF1**	4324,58	9663,03	57919,47	24309,48	5764,03	3514,54	24054,14	4639,285	19414,855
**TXNDC5**	25346,77	16493,71	40487,01	29580,33	8782,7	8391,57	27976,955	8587,135	19389,82
**MRPL4**	11299,83	9864,98	44077,8	31630,06	7168,17	2499,27	24218,1675	4833,72	19384,4475
**NIBAN2**	11411,93	14124,21	33139,05	36489,89	6703,64	2474,52	23791,27	4589,08	19202,19
**PICALM**	28893,2	12815,76	37875,69	30943,05	5759,29	11351,87	27631,925	8555,58	19076,345
**KIT**	5009,37	32695,92	28962,3	20758,49	1,27	5617,95	21856,52	2809,61	19046,91
**LENG8**	16120,72	34851,35	53727,52	22615,44	13241,67	12978,68	31828,7575	13110,175	18718,5825
**FERMT3**	30472,24	15852,5	52501,23	23922,05	12778,44	11866,29	30687,005	12322,365	18364,64
**ABCC4**	41496,34	14363,88	33825,37	21393,26	1400,91	17500,34	27769,7125	9450,625	18319,0875
**PGD**	23046,45	16873,93	32810,33	30545,47	3986,7	11583,39	25819,045	7785,045	18034
**NUCB1**	12541,44	17816,12	51246,9	20000,61	6940,07	7909,61	25401,2675	7424,84	17976,4275
**SERPINB1**	7503,78	15765,86	72090,81	7378,91	9084,82	6346,48	25684,84	7715,65	17969,19
**UBL5**	7398,98	6068,51	51056,77	27077,6	6266,32	3617,31	22900,465	4941,815	17958,65
**RPS6**	86865,52	155529	147766,62	479062,4	325583,79	73128,39	217305,89	199356,09	17949,8
**PPIB**	32460,54	26362	66897,21	28583,23	30922,41	10405,01	38575,745	20663,71	17912,035

**LYL1**	21596,41	14406,24	35240,74	37633,92	9758,19	8988,28	27219,3275	9373,235	17846,0925
**KLF1**	11866,03	14885,54	24172,92	25152,67	33,42	2357,54	19019,29	1195,48	17823,81
**UROD**	18522,56	17173,34	32608,12	21598,84	2444,49	6869,94	22475,715	4657,215	17818,5
**TGFB1**	29688,98	19364,54	60026,57	29992,91	21293,16	12639,65	34768,25	16966,405	17801,845
**IFITM3**	1750,98	41,26	66262,88	2553,58	367,53	210,59	17652,175	289,06	17363,115
**MYL4**	18697,35	7628,08	35224,23	8272,16	0	763,04	17455,455	381,52	17073,935
**HLA-A**	18497,63	34308,9	155983,18	5123,77	65173,22	7748,29	53478,37	36460,755	17017,615
**SLC43A3**	44737,31	13937,08	18302,88	34843,79	16999,54	4898,1	27955,265	10948,82	17006,445
**HERC2**	29043	30974,56	40670,2	42858,1	6849,58	31230,82	35886,465	19040,2	16846,265
**RPS18**	132317,1	126115,7	225480,91	124296,7	246658,6	24834,22	152052,6	135746,41	16306,19
**MPP1**	17770,44	8996,38	21805,83	30134,35	2431,13	4310,25	19676,75	3370,69	16306,06
**FAU**	31884,18	32368,37	56408,71	34945,07	32828,18	12495,43	38901,5825	22661,805	16239,7775
**RPL36**	31005,67	38576	63360,66	81051,32	65877,16	8664,27	53498,4125	37270,715	16227,6975
**PRRC2A**	31118,54	45819,34	37388,48	48025,52	27411,76	21464,41	40587,97	24438,085	16149,885
**BLVRB**	11743,69	7836,67	27828,88	24668,88	1978,28	1972,77	18019,53	1975,525	16044,005
**HNRNPM**	37599,92	38272,57	34386,28	48239,01	17838,36	29330,02	39624,445	23584,19	16040,255
**PRSS57**	12354,84	28850,48	36623,97	11128,11	28,72	12967,25	22239,35	6497,985	15741,365
**GABRE**	7920,15	10658,76	24617,84	19272,01	0	0	15617,19	0	15617,19
**SRSF3**	40907,23	39744,38	38462,86	29698,81	20242,55	23089	37203,32	21665,775	15537,545
**PDE4DIP**	5117,58	18169,17	4771,88	35588,81	655,61	196,76	15911,86	426,185	15485,675
**CALM3**	22592,58	36014,02	49700,66	51229,38	21765,73	27141,69	39884,16	24453,71	15430,45
**HYOU1**	23781,84	13672,85	28571,41	24036,09	6174,95	8065,13	22515,5475	7120,04	15395,5075
**PDLIM1**	14864,35	27274,18	9249,31	11160,09	94,85	663,22	15636,9825	379,035	15257,9475
**LAPTM4B**	24144,53	41821,32	12996,52	19355,57	8676,95	10000,08	24579,485	9338,515	15240,97
**RAB6B**	7274,26	14012,78	5455,49	34258,02	51,2	47,91	15250,1375	49,555	15200,5825
**TAGLN2**	70884,62	69324,06	71290,69	86197,56	63511,09	54953,41	74424,2325	59232,25	15191,9825
**EIF4B**	43748,42	83380,24	35922,93	52781,04	46291,98	31464,52	53958,1575	38878,25	15079,9075
**PPAN**	7151,89	6680,02	36967,39	27587,46	7349,9	1697,95	19596,69	4523,925	15072,765
**TXNIP**	21009,57	26882,75	33590,06	17267,15	5938,52	13652,8	24687,3825	9795,66	14891,7225
**FAM117A**	8501,81	10463,87	36464,08	15628,69	2399,61	3606,05	17764,6125	3002,83	14761,7825
**CKB**	19777,2	10041,14	29483,34	12156,89	1042,29	5467,58	17864,6425	3254,935	14609,7075
**ODC1**	32871,71	22658,86	20855,56	10314,24	7326,42	6849,31	21675,0925	7087,865	14587,2275
**RPL7A**	143885,4	159960,3	125499,02	137644,4	143397,32	111099,8	141747,27	127248,535	14498,735

**NCL**	125723,2	101973,7	83934,12	78472,54	107048,48	59035,25	97525,8725	83041,865	14484,0075
**VCP**	37308,14	27593,59	28921,24	25706,34	10401,57	20422,94	29882,3275	15412,255	14470,0725
**CTNNBL1**	16507	11187,97	39537,56	23018,56	4672,87	11724,02	22562,7725	8198,445	14364,3275
**CA1**	25146,56	5245,96	7854,75	18993,11	0	6,38	14310,095	3,19	14306,905
**MIF**	50096,48	54904,41	58487,75	36919,32	57010,27	14746,41	50101,99	35878,34	14223,65
**PTPN1**	11925,32	6712,81	27920,77	36075,11	2623,81	10457,02	20658,5025	6540,415	14118,0875
**STIP1**	31805,66	21802,4	21650,68	28289,8	12207,93	11391,26	25887,135	11799,595	14087,54
**SRSF2**	45342,45	64015,8	32969,8	34365,78	30508,4	29705,29	44173,4575	30106,845	14066,6125
**ARL6IP4**	21310,29	14236,36	42002,02	16416,46	10131,41	8731,99	23491,2825	9431,7	14059,5825
**MCM3**	34806,16	22406,29	22126,44	42734,56	11414,6	21623,62	30518,3625	16519,11	13999,2525
**HPGD**	15613,64	6925,72	16321,99	17125,04	0	2,47	13996,5975	1,235	13995,3625
**COX6B1**	11617,13	7939,6	54427,03	16566,63	12581,03	4858,11	22637,5975	8719,57	13918,0275
**PKN1**	27270,03	30592,13	35793,61	41843,26	22875,7	17265,93	33874,7575	20070,815	13803,9425
**EIF3B**	26346,16	36282,6	33877,73	30337,55	20454,76	15565,31	31711,01	18010,035	13700,975
**TIMP1**	24248,58	16842,79	24674,42	16178,66	8468,46	5242,74	20486,1125	6855,6	13630,5125
**ATP5F1A**	55869,06	53141,75	49139,67	51602,39	48324,14	29360,6	52438,2175	38842,37	13595,8475
**BTK**	16313,57	8483,59	40326,27	15507,7	9928,96	3254,53	20157,7825	6591,745	13566,0375
**PLIN2**	4674,31	7382,87	9743,98	66287,7	15287,62	1969,97	22022,215	8628,795	13393,42
**MARS1**	15387,71	10299,59	19349,14	20672,12	2512,53	3589,6	16427,14	3051,065	13376,075
**RRP1B**	14052,9	6887,95	33699,56	17203,21	3341,43	5901,71	17960,905	4621,57	13339,335
**MTSS1**	10807,44	26610,42	8434,1	8450,38	400,55	229,25	13575,585	314,9	13260,685
**JUP**	4749,42	26675,16	26025,2	8118,5	83,19	6234,44	16392,07	3158,815	13233,255
**FXYD5**	14937,58	5908,86	39004,54	12639,48	5870,87	3914,29	18122,615	4892,58	13230,035
**CAPN1**	15709,2	25621,04	18911,92	21405,88	6194,05	8269,78	20412,01	7231,915	13180,095
**KEAP1**	9817,21	9069,41	34331,35	29491,94	8575,38	6557,19	20677,4775	7566,285	13111,1925
**ADCY7**	10160,87	15113	5527,23	29310,97	661,42	3190,94	15028,0175	1926,18	13101,8375
**RPS25**	69825,12	46154,81	86787,86	70718,99	90041,05	20556,48	68371,695	55298,765	13072,93
**TUBA1C**	40317,31	24253,51	41299	35428,79	22231,77	22297,64	35324,6525	22264,705	13059,9475
**PPP6R1**	23296,93	36813,92	29385,91	46984,32	21051,98	21090,39	34120,27	21071,185	13049,085
**LUC7L3**	22155,23	24006,12	61199,71	22300,74	18190,82	20580,97	32415,45	19385,895	13029,555
**IGF2BP1**	22578,73	18354,41	11347,2	0	105,43	1,66	13070,085	53,545	13016,54
**COTL1**	36484,38	16955,25	33248,09	23982,36	24084,5	5247,42	27667,52	14665,96	13001,56
**TTC3**	16061,38	17536,48	40232,75	13231,25	6259,81	11388,01	21765,465	8823,91	12941,555

**RPS12**	75438,19	75845,09	88365,26	93071,99	105136,26	35439,97	83180,1325	70288,115	12892,0175
**RPS26**	23857,09	14296,66	59392,39	18272,9	17542,46	14632,2	28954,76	16087,33	12867,43
**PDIA6**	35574,16	24712,9	38894,14	15386,53	22011,12	9613,09	28641,9325	15812,105	12829,8275
**SERF2**	34787,13	34044,6	78251,91	33148,6	45892,25	18638,11	45058,06	32265,18	12792,88
**VEGFA**	10179,75	2370,97	35450,02	21942,14	7348,43	2406,65	17485,72	4877,54	12608,18
**CPSF1**	17990,43	30011,01	39143,1	26400,27	24550,02	7082,29	28386,2025	15816,155	12570,0475
**SLC44A2**	4859,12	4206,12	26635,67	25332,96	664,1	4717,32	15258,4675	2690,71	12567,7575
**RPS4Y1**	29944,42	0	1,11	21142,03	473,83	8,75	12771,89	241,29	12530,6
**DDB1**	26939,66	28438,43	28176,29	28917,47	12724,39	18516,77	28117,9625	15620,58	12497,3825
**CCT8**	60036,54	34455,71	93282,46	41283,91	73995,64	15646,29	57264,655	44820,965	12443,69
**YBX3**	18951,22	17347,05	29550,93	15587,89	15860,62	6,46	20359,2725	7933,54	12425,7325
**PRKDC**	76903,63	45394,66	46558,18	52647,08	62784,19	23222,86	55375,8875	43003,525	12372,3625
**BAG6**	19727,95	23944,05	37266,31	28310,35	22199,08	7817,89	27312,165	15008,485	12303,68
**EIF4G1**	45957,9	47287,95	33272,36	29252,64	28743,74	24579,71	38942,7125	26661,725	12280,9875
**ARHGDIA**	26831,19	32897,78	29041,91	27781,68	20167,28	13548,25	29138,14	16857,765	12280,375
**HSP90B1**	76595,22	74104,85	64826,95	37900,29	73354,16	28847,27	63356,8275	51100,715	12256,1125
**TIE1**	11902,14	6124,95	28109,47	3479,61	0	301,55	12404,0425	150,775	12253,2675
**WARS1**	12801,74	9234,57	22930,87	27050,49	5492,21	6354,86	18004,4175	5923,535	12080,8825
**GCN1**	20803,55	24447,64	25759,77	24856,71	7973,72	15847,41	23966,9175	11910,565	12056,3525
**ALDH1A1**	18690,3	13411,02	54447,5	4506,03	298,21	21226,44	22763,7125	10762,325	12001,3875
**ANGPTL6**	2304,2	4692,81	15228,41	26099,88	9,65	221,56	12081,325	115,605	11965,72
**MPIG6B**	24458,88	2504	15648,01	6525,54	1,6	682,85	12284,1075	342,225	11941,8825
**AARS1**	29571,65	14354,37	16163,77	20708,15	11339,8	5207,6	20199,485	8273,7	11925,785
**TOMM40**	18957,65	16910,82	29615,26	19911,25	9869,14	9045,01	21348,745	9457,075	11891,67
**VPS51**	7092,08	27136,32	22382,42	16573,13	8458,81	4467,35	18295,9875	6463,08	11832,9075
**PRMT1**	25241,56	30760,01	35928,32	24699,01	23547,68	11147,24	29157,225	17347,46	11809,765
**NOP53**	22468,97	41024,94	55915,29	26255,46	35137,62	14093,34	36416,165	24615,48	11800,685
**FLOT2**	11666,28	16956,31	29002,18	13655,97	6596,48	5741,02	17820,185	6168,75	11651,435
**UBA1**	46635,44	28119,46	24355,72	22273,22	17960,37	19481,87	30345,96	18721,12	11624,84
**FARP1**	6103,35	86,42	41810,02	228,45	55,58	811,46	12057,06	433,52	11623,54
**RAB8A**	11505,93	11056,88	25296,43	19163,14	2640,85	7639,98	16755,595	5140,415	11615,18
**PRPF19**	21989,43	24131,18	23684,41	26785,68	9306,22	15795,69	24147,675	12550,955	11596,72
**FBL**	26329,68	21122,02	28847	28678,42	19708,69	9645,26	26244,28	14676,975	11567,305

**LMNA**	5127,45	22241,25	10151,49	52949,2	19715,77	2403,39	22617,3475	11059,58	11557,7675
**LONP1**	18559,89	17834,65	23647,22	42209,8	22833,43	5204,96	25562,89	14019,195	11543,695
**WDR1**	27285,12	33396,71	19986,81	24456,37	16935,91	12625,2	26281,2525	14780,555	11500,6975
**SOS1**	21875,22	9955,97	10196,01	14160,23	1080,33	4039,88	14046,8575	2560,105	11486,7525
**RUVBL2**	15030,8	24493,35	25977,28	26863,56	15386,94	7846,74	23091,2475	11616,84	11474,4075
**RNF130**	18014,87	19052,78	23112,39	20404,93	5562,18	11888,21	20146,2425	8725,195	11421,0475
**HMGB1**	39428,33	63567,28	45302,03	43578,25	36363,7	36928,09	47968,9725	36645,895	11323,0775
**SAMSN1**	9991,5	6266,92	27387,4	6801,31	853,71	1726,36	12611,7825	1290,035	11321,7475
**TFDP1**	24792,47	12042,07	9986,47	19537,8	10589,48	92,61	16589,7025	5341,045	11248,6575
**CAPNS1**	12808,74	15163,39	47414,38	16338,2	14153,91	9232,5	22931,1775	11693,205	11237,9725
**MEST**	14650,61	6807,21	17995,73	5943,01	181,64	41,38	11349,14	111,51	11237,63
**NOC2L**	15134,84	12776,38	20673,2	22708,42	7326,03	5847,53	17823,21	6586,78	11236,43
**STXBP5**	30901,81	12405,58	8345,9	18658,67	166,03	12542,39	17577,99	6354,21	11223,78
**CLIC1**	35434,96	34017,37	54659,12	54233,4	54890,94	11883,75	44586,2125	33387,345	11198,8675
**UNC13D**	21084,13	30357,79	26671,5	26725,96	19454,83	10576,53	26209,845	15015,68	11194,165
**RPL24**	65270,8	84510,2	91075,15	47052,86	97929,58	23736,02	71977,2525	60832,8	11144,4525
**STXBP2**	8908,36	12939,57	19168,2	30172,59	13319,69	46,16	17797,18	6682,925	11114,255
**FSCN1**	9954,34	3897,9	35439,47	23019,49	1079,34	12903,66	18077,8	6991,5	11086,3
**ATP7B**	18266,46	13398,05	3498,83	11801,26	59,83	1266,65	11741,15	663,24	11077,91
**CD164**	41800,2	36125,71	34360,59	15714,78	15272,33	26574,31	32000,32	20923,32	11077
**SNRPD2**	18046,95	16994,45	44770,28	21988,38	19112,42	9728,54	25450,015	14420,48	11029,535
**MACROH2A1**	36670,21	42655,63	46006,46	33655,41	19720,65	37770,54	39746,9275	28745,595	11001,3325
**TPM3**	42388,85	39197,16	58887,02	69795,84	43824,63	39437,62	52567,2175	41631,125	10936,0925
**RCC2**	32510,3	28334,47	34935,56	29396,73	10902,52	29828,86	31294,265	20365,69	10928,575
**COL18A1**	30725,75	2117,43	11199,8	5922,38	2242,57	990,17	12491,34	1616,37	10874,97
**POLR2A**	14397,63	30012,46	22809,21	21005,76	9111,92	13401,62	22056,265	11256,77	10799,495
**LIMD2**	10474,19	14157,16	15200,38	16059,98	2172,99	4237,65	13972,9275	3205,32	10767,6075
**SERBP1**	57259,84	57518,8	48078,33	59499,45	60863,9	28812,74	55589,105	44838,32	10750,785
**GATD3B**	4943,46	4426,54	36561,49	14878,27	4067,02	4893,93	15202,44	4480,475	10721,965
**RPS7**	76167,7	69302,02	84842,13	34305,75	94867,42	16004,56	66154,4	55435,99	10718,41
**KPNB1**	52604,03	40961,81	31452,04	38230,52	23576,6	36633,25	40812,1	30104,925	10707,175
**JUND**	10941,71	18074,8	20036,02	19814,9	8875,57	4250,32	17216,8575	6562,945	10653,9125
**EI24**	36827,98	11479,87	5946,33	11013,94	5315,59	6015,28	16317,03	5665,435	10651,595

**NOP56**	37480,94	20299,61	20533,21	19501,67	16151,75	11480,44	24453,8575	13816,095	10637,7625
**MGAT4B**	20927,97	16387,43	14321,81	25167,72	4713,59	12472,93	19201,2325	8593,26	10607,9725
**TALDO1**	32147,29	13019,11	29539	32022,28	17717,48	14514,67	26681,92	16116,075	10565,845
**RRBP1**	8046,21	12582,65	26324,84	24889,01	7654,97	7171,62	17960,6775	7413,295	10547,3825
**SREBF1**	12679,44	19069,27	28596,26	12991,88	9446,36	6321,45	18334,2125	7883,905	10450,3075
**GART**	21053,73	13657,14	12683,67	32952,86	9776,73	9542,24	20086,85	9659,485	10427,365
**UBASH3B**	7215,61	3005,32	16275,37	21318,61	628,72	2492,1	11953,7275	1560,41	10393,3175
**RACK1**	111210	124999,5	88823,67	112938,9	136953,8	61353,31	109493,043	99153,555	10339,4875
**HS6ST1**	8174,69	14888,55	11443,18	13928,44	286,36	3271,54	12108,715	1778,95	10329,765
**ICAM5**	2385,61	4520,39	20746,47	15857,75	221,12	1040,36	10877,555	630,74	10246,815
**CFH**	11750,68	7536,43	14367,76	7930,07	39,38	367,98	10396,235	203,68	10192,555
**PITX1**	48,26	23989,38	6966,28	10527,96	9,7	425,31	10382,97	217,505	10165,465
**BCAT1**	22561,25	20833,04	15257,33	12564,47	646,46	14651,81	17804,0225	7649,135	10154,8875
**H2AX**	21353,78	17975,5	16054,45	15551,68	5192,75	9971,74	17733,8525	7582,245	10151,6075
**ATP2A3**	13677,22	35088,45	26879,48	27666,88	4839,19	26641,48	25828,0075	15740,335	10087,6725
**IFITM1**	2168,91	7716,48	34345,59	3844,69	2,63	3879,31	12018,9175	1940,97	10077,9475
**AP1B1**	14930,22	12812,83	21665,51	16052,12	4577,62	8051,9	16365,17	6314,76	10050,41
**DKC1**	22204,98	15991,06	23103,55	27619,9	13057,01	11359,56	22229,8725	12208,285	10021,5875
**MYO9B**	7821,07	11949,85	21359,32	42710,79	10014,65	11863	20960,2575	10938,825	10021,4325
**HBS1L**	9855,95	8664,41	11753,02	29697,19	3500,57	6596,14	14992,6425	5048,355	9944,2875
**ASAH1**	27266,58	9775,25	20234,96	8269,79	6970,25	5917,38	16386,645	6443,815	9942,83
**PLA2G6**	8275,48	16404,71	13075,6	9520,61	928,23	2861,2	11819,1	1894,715	9924,385
**UBAC1**	11102,3	9448,95	13350,52	20714,3	3079,46	4468,66	13654,0175	3774,06	9879,9575
**YIF1B**	8611,08	10583,14	21780,67	20692,96	5223,13	5893,44	15416,9625	5558,285	9858,6775
**IL2RG**	13640,48	17626,71	15370,06	6040,11	690,27	6081,15	13169,34	3385,71	9783,63
**U2AF2**	24456,95	32086,44	29497,34	28798,47	22292,48	15609,39	28709,8	18950,935	9758,865
**ATP5PO**	24466,72	11637,49	37161,85	16124,62	16949,38	8243,49	22347,67	12596,435	9751,235
**HNRNPUL1**	28988,68	32728,33	43919,99	35209,29	21117	29907,26	35211,5725	25512,13	9699,4425
**CTSH**	3294,14	20877,02	9159,76	12735,21	3206,69	433,1	11516,5325	1819,895	9696,6375
**PLCXD1**	27687,55	9219,35	5505,85	8013,33	897,66	4925,35	12606,52	2911,505	9695,015
**ITGA4**	44502,58	26532,41	36587,07	11511,71	9042,62	31196,13	29783,4425	20119,375	9664,0675
**PKM**	111286,5	78650,97	157400,58	51153,43	108480,73	71463,61	99622,8625	89972,17	9650,6925
**CCT3**	45146,4	29983,56	30176,21	43522,67	37860,19	17324,39	37207,21	27592,29	9614,92

**PNN**	23572,83	27026,73	30302,15	17069,24	9851,53	20113,42	24492,7375	14982,475	9510,2625
**AGPAT3**	12217,17	10091,33	16925,67	17171,43	4275,69	4921,64	14101,4	4598,665	9502,735
**KRI1**	4255,33	6320,73	28505,57	18624,34	4328,11	5528,5	14426,4925	4928,305	9498,1875
**IRX1**	8324,96	10520,88	13678,36	5456,82	0	0	9495,255	0	9495,255
**ADD2**	2760,6	23181,84	3665,53	14481,76	568,51	2526,53	11022,4325	1547,52	9474,9125
**ATP1A1**	37334,74	22910,21	37814,66	30039,54	19346,82	25776,79	32024,7875	22561,805	9462,9825
**NUDT3**	13129,18	16738,21	20882,58	9228,99	6971,02	4214,38	14994,74	5592,7	9402,04
**DLC1**	11842,25	13753,41	3232,3	8730,05	0	18,04	9389,5025	9,02	9380,4825
**IFITM2**	11007,79	10414,75	28958,53	8428,93	6572,95	4139,57	14702,5	5356,26	9346,24
**ATP5F1B**	83182,71	63785,55	55617,52	62295,42	75442,55	38328,91	66220,3	56885,73	9334,57
**CCT2**	29901,66	25928,26	22998,39	29350,6	19371,48	16051,8	27044,7275	17711,64	9333,0875
**ANPEP**	4380,36	6255,61	17977,22	10840,01	994,31	206,98	9863,3	600,645	9262,655
**PTPN7**	23554,48	26829,41	17454,17	32242,85	18718,7	12837,73	25020,2275	15778,215	9242,0125
**STOM**	18462,71	11379,11	7061,5	24916,96	2265,01	10175,87	15455,07	6220,44	9234,63
**NECTIN2**	6349,04	4635,64	31775,65	8941,67	1146,74	6491,92	12925,5	3819,33	9106,17
**UBXN6**	6146,92	6780,12	14074,72	31641,9	7785,67	3374,61	14660,915	5580,14	9080,775
**CARM1**	6348,98	5604,74	16043,51	20399,38	3959,6	2098,71	12099,1525	3029,155	9069,9975
**PA2G4**	36637,35	28522,24	21444,55	32323,52	23928,67	17426,51	29731,915	20677,59	9054,325
**ICAM3**	4332,08	5627,56	28147,18	10350,93	963,84	5159,09	12114,4375	3061,465	9052,9725
**CD151**	11093,5	13400,39	20167,13	11982,78	6617,52	3648,17	14160,95	5132,845	9028,105
**DCTN1**	12919,65	14916,13	28301,27	16788,57	9940,26	8527,25	18231,405	9233,755	8997,65
**MYH10**	14971,49	16738,94	12080,27	12458,94	49,06	10090,36	14062,41	5069,71	8992,7
**KDM1A**	19074,57	21506,32	32729,55	17035,2	12611,66	14585,68	22586,41	13598,67	8987,74
**RBM3**	34331,51	25819,94	16863,45	17138,14	20440,13	8691,8	23538,26	14565,965	8972,295
**GOLGA8A**	12377,55	28796,96	4519,22	469,36	4660,65	596,23	11540,7725	2628,44	8912,3325
**LDHA**	132098,4	121245,3	66014,06	84362,92	125635,1	58453,77	100930,175	92044,435	8885,74
**SRPRA**	18737,9	7636,05	16301,24	23853,75	9183,52	6382,5	16632,235	7783,01	8849,225
**TMBIM1**	8698,01	11813,94	7344,44	15899,65	3294,76	890,97	10939,01	2092,865	8846,145
**ST3GAL2**	5774,23	16253,18	8553,99	10338,4	1572,3	1392,9	10229,95	1482,6	8747,35
**TUFM**	27883,1	32307,07	23262,18	23301,92	19981,69	15916,77	26688,5675	17949,23	8739,3375
**AP2M1**	27914,74	20741,55	39150,51	26764,19	24076,94	15778,49	28642,7475	19927,715	8715,0325
**ATAD3A**	11138,3	11781,09	16284,09	15921,87	5797,57	4392,24	13781,3375	5094,905	8686,4325
**MGAT3**	7348,28	7761,28	3914,04	16971,78	4,77	621,49	8998,845	313,13	8685,715

**CDKN2A**	0	2032,59	49240,81	0	338,56	7929,71	12818,35	4134,135	8684,215
**SEC61A1**	24659,37	19713,63	33054,02	21534,16	15042,34	17069,88	24740,295	16056,11	8684,185
**CCT6A**	34797,68	22996,41	18474,09	20950,05	19226,13	12044,85	24304,5575	15635,49	8669,0675
**EIF3I**	22325,97	17611,9	16564,12	21424,56	14069,55	7563,64	19481,6375	10816,595	8665,0425
**PLIN3**	4905,47	11481,7	15412,79	24007,06	5866,74	4719,14	13951,755	5292,94	8658,815
**UBR4**	24659,31	22861,35	64870,34	56780,45	22881,49	44388,13	42292,8625	33634,81	8658,0525
**PECAM1**	11248,99	11493,49	13794,7	5100,84	2338,8	1202	10409,505	1770,4	8639,105
**EPB41**	10816,92	14435,96	13158,8	17322,46	2006,39	8623,49	13933,535	5314,94	8618,595
**FAM178B**	955,01	3326,12	4828,67	25443,1	0	64,84	8638,225	32,42	8605,805
**PHB2**	24978,47	30363,53	21840,64	16433,31	19973,96	9675,49	23403,9875	14824,725	8579,2625
**SYNGR2**	7729,71	10989,36	15921,93	22788,91	7439,85	4143,52	14357,4775	5791,685	8565,7925
**PSMD3**	16989,47	13953,57	14185,77	15842,48	6597,49	6789,54	15242,8225	6693,515	8549,3075
**PHB**	23781,62	16707,8	19130,13	17938,51	13851,14	7829,89	19389,515	10840,515	8549
**SRSF1**	29685,45	36234,4	19599,09	32091,02	12384,44	29328,26	29402,49	20856,35	8546,14
**AP1M1**	7858,99	8357,89	15868,27	28757,61	7416,08	5949,1	15210,69	6682,59	8528,1
**VARS1**	22657,21	19455,05	12984,03	20708,3	17240,96	3637,78	18951,1475	10439,37	8511,7775
**EIF2S3**	47769,58	18994,08	16970,17	22902,09	21948,8	14380,87	26658,98	18164,835	8494,145
**CHCHD2**	35158,76	29534,21	30686,55	19299,73	25125,31	15280,31	28669,8125	20202,81	8467,0025
**SNRNP70**	19863,83	30982,16	34516,17	22715,44	22443	14679,34	27019,4	18561,17	8458,23
**GYPC**	9266,92	11050,77	7553,71	17959,66	3086,07	2936	11457,765	3011,035	8446,73
**SNX5**	23340,52	24467,67	12181,96	18338,68	12366,94	9920,23	19582,2075	11143,585	8438,6225
**CTNNA1**	12265,69	14383,29	14263,64	10684,65	8929,61	20,34	12899,3175	4474,975	8424,3425
**STAT5B**	13148,79	18106,18	16783,04	18599,85	2878,06	13609,09	16659,465	8243,575	8415,89
**USP48**	16583,38	12234,09	17714,75	18433,42	5903,51	9757,27	16241,41	7830,39	8411,02
**BCAP31**	15273,94	13036,13	24440,89	23190,56	13019,02	8138,81	18985,38	10578,915	8406,465
**CLUH**	7046,96	19101,28	17557,54	16391,45	9163,51	4120,31	15024,3075	6641,91	8382,3975
**PPP2R1A**	15687,4	27668,22	38626,24	17667,47	21433,82	11636,71	24912,3325	16535,265	8377,0675
**ZNF385A**	11936,78	13527,53	11452,51	8159,43	958,82	4860,09	11269,0625	2909,455	8359,6075
**CTBP2**	10826,69	12763,9	4933,31	10935,5	3062,01	30,9	9864,85	1546,455	8318,395
**PPP1CA**	25612,39	19617,39	21157,95	17570,73	11729,49	13621,87	20989,615	12675,68	8313,935
**ITGB3**	13277,23	1522,6	13652,44	5681,28	18,12	441,56	8533,3875	229,84	8303,5475
**RCL1**	2169,33	3712,94	2749,96	31370,26	2013,94	1389,02	10000,6225	1701,48	8299,1425
**FARSA**	14940,6	8710,01	17792,61	28013	10613,12	7544,3	17364,055	9078,71	8285,345

**RNPS1**	23301,23	21157,7	20069,37	22410,88	19394,51	7569,5	21734,795	13482,005	8252,79
**FCER1A**	23805,77	1207,88	17342,52	6888,27	2,96	8152,64	12311,11	4077,8	8233,31
**STT3A**	25883,38	10109,73	15054,5	29778,08	11914,47	12065,59	20206,4225	11990,03	8216,3925
**PLOD1**	6567,06	13009,52	24030,43	10865,97	9519,29	1344,91	13618,245	5432,1	8186,145
**PYCR1**	10010,19	6624,83	8757,64	11354,88	2014,16	81,93	9186,885	1048,045	8138,84
**DBN1**	15373,45	9397,53	22488,55	15426,16	10475,55	4615,24	15671,4225	7545,395	8126,0275
**ABCF1**	12387,73	10837,32	17430,32	17880,32	9002	4016,3	14633,9225	6509,15	8124,7725
**SERPINF1**	386,44	2070,78	30001,55	83,58	20,13	17,36	8135,5875	18,745	8116,8425
**EMP3**	12400,51	10663,72	23563,42	16580,34	12074,6	3296,36	15801,9975	7685,48	8116,5175
**NUMA1**	23809,3	26489,88	27609,17	30149,86	14408,82	23424,38	27014,5525	18916,6	8097,9525
**EPOR**	157,18	1473,6	14387,8	24485,28	1392,85	2731,9	10125,965	2062,375	8063,59
**PSMD8**	16584,04	11574,58	20243,31	18497,17	10280,3	7045,91	16724,775	8663,105	8061,67
**BANF1**	25628,47	11099,11	19084,74	12378,3	9210,66	8776,55	17047,655	8993,605	8054,05
**MAN1A1**	7539,13	4584,92	4726,59	16528	579,94	3,5	8344,66	291,72	8052,94
**TYK2**	7070,92	6552,91	33657,56	28353,89	15848,26	5913,12	18908,82	10880,69	8028,13
**NARF**	5444,51	10614,95	32058,6	12751,38	7262,51	7133,14	15217,36	7197,825	8019,535
**SAE1**	17684,1	16479,8	25582,4	20983,75	7448,28	16912,75	20182,5125	12180,515	8001,9975
**CTSB**	10425,29	14118,77	20219,6	30608,9	17148,29	4551,42	18843,14	10849,855	7993,285
**CTSD**	20609,02	15551,59	28722,41	18092,85	15163,87	10431,24	20743,9675	12797,555	7946,4125
**UCP2**	16829,44	4182,36	9195,06	14883,35	1943,97	4734,48	11272,5525	3339,225	7933,3275
**GSE1**	15387,15	24661,23	12480,29	19064,84	4139,74	15871,7	17898,3775	10005,72	7892,6575
**HNRNPC**	65003,91	63962,93	54597,61	56945,12	74678,93	29798,74	60127,3925	52238,835	7888,5575
**CAD**	29344,42	15430,5	23072,9	9576,25	14733,31	8281,01	19356,0175	11507,16	7848,8575
**TBCD**	10974,68	10382,37	23619,25	7861,42	3322,07	7404,16	13209,43	5363,115	7846,315
**HUWE1**	42211,15	26950,31	25083,24	25768,93	16814,72	27559,92	30003,4075	22187,32	7816,0875
**PIM2**	7103,83	6757,29	13882,32	12280,66	1458,78	2944,42	10006,025	2201,6	7804,425
**ISYNA1**	4385,88	17174,48	8043,61	23974,94	2102,33	9138,87	13394,7275	5620,6	7774,1275
**FBRSL1**	13819,84	19289,76	16074,47	14235,74	7983,56	8184,27	15854,9525	8083,915	7771,0375
**GSPT1**	20128,52	16063,94	13579,4	23368,43	15543	5520,51	18285,0725	10531,755	7753,3175
**MTA2**	17395,76	18588,95	21023,56	16193,35	8997,23	12161,73	18300,405	10579,48	7720,925
**EIF1**	25762,55	33751,33	43401,12	32846,57	38460,71	14029,65	33940,3925	26245,18	7695,2125
**KCNK6**	4313,63	3681,21	20068,95	16539,25	2382,99	4529,83	11150,76	3456,41	7694,35
**SDF4**	15271,52	11381,22	23154,37	14225,86	9600,31	7053,48	16008,2425	8326,895	7681,3475

**MBOAT7**	5963,68	7508,16	20122,59	17115,12	2766,15	7255,2	12677,3875	5010,675	7666,7125
**GRB10**	6921,42	10051,7	6904,79	8097,82	541,03	125,24	7993,9325	333,135	7660,7975
**SERP1**	14653,32	20276,21	20425	16749,56	13340,22	7390,4	18026,0225	10365,31	7660,7125
**PRAME**	24497,07	669,38	12,83	5453,25	16,29	7,53	7658,1325	11,91	7646,2225
**MPST**	8129,32	23139,68	9840,91	9797,42	6101,02	4127,02	12726,8325	5114,02	7612,8125
**TPSB2**	26,64	15520,05	2579,29	15168,61	1,88	1433,72	8323,6475	717,8	7605,8475
**SARS1**	10994,11	10604,06	13190,48	18876,97	7163,39	4469,13	13416,405	5816,26	7600,145
**MT-ND5**	111759,3	179748,1	74015,61	113404,8	108087,45	116205,3	119731,968	112146,38	7585,5875
**TNFSF13B**	331,12	5,14	36724,51	272,73	2311,23	1199,51	9333,375	1755,37	7578,005
**DRAP1**	14922,92	6987,68	16195,37	13856,53	4149,98	6675,68	12990,625	5412,83	7577,795
**FST**	13359,16	534,98	8398,87	8108,93	29,05	30,44	7600,485	29,745	7570,74
**NUP62**	12674,85	25038,53	17907,56	16204,31	8988,84	11797,36	17956,3125	10393,1	7563,2125
**EIF3K**	14516,15	11546,04	30096,85	17015,38	14939,83	6523,77	18293,605	10731,8	7561,805
**ICAM4**	3146,81	7747,15	21624,68	6805,36	7,24	4582,33	9831	2294,785	7536,215
**NCF4**	25855,1	16230,79	34649,99	14117,01	29046,64	1326,94	22713,2225	15186,79	7526,4325
**NRROS**	10927,28	11657,68	13411,13	12049,11	3407,37	5571,41	12011,3	4489,39	7521,91
**YARS1**	15554,94	15231,06	33402,79	20421,92	11919,16	15367,34	21152,6775	13643,25	7509,4275
**SPTBN2**	320,52	27802,98	643,95	33871,76	18,49	16299,12	15659,8025	8158,805	7500,9975
**PSMD2**	22423,44	16657,27	15098,38	15441,37	8635,24	11194,4	17405,115	9914,82	7490,295
**SUPT5H**	8454,81	13676,28	28369,7	14232,17	8582,51	8824,63	16183,24	8703,57	7479,67
**GANAB**	33569,88	39550,46	43659,02	28754,46	29231,66	28600,36	36383,455	28916,01	7467,445
**MLLT1**	7361,46	10717,77	9128,09	30119,97	6597,95	7131,16	14331,8225	6864,555	7467,2675
**PARP1**	43655,9	25600,96	28961,93	29958,26	16320,61	32839,5	32044,2625	24580,055	7464,2075
**ACTB**	383943,6	354823,2	356225,83	338545,4	516779,62	185107,9	358384,515	350943,775	7440,74
**FASN**	86296,1	61085,87	53122,44	36505,49	76786,68	26849,2	59252,475	51817,94	7434,535
**MCM3AP**	10833,7	11737,88	17333,49	12570,86	4759,92	6664,19	13118,9825	5712,055	7406,9275
**ACSL4**	28932,8	2788,86	14292,55	10827,89	5902,13	7722,21	14210,525	6812,17	7398,355
**LTBP4**	2558,22	2096,73	34666,3	7475,6	4096,39	4539,58	11699,2125	4317,985	7381,2275
**ANAPC5**	17405,13	15293,08	25225,93	14738,76	10534,28	11118,59	18165,725	10826,435	7339,29
**PFAS**	14406,37	15442,64	7168,67	8664,93	5674,9	2494,54	11420,6525	4084,72	7335,9325
**EEF1G**	186314,5	172788,5	219187,18	171931,9	269578,16	90862,21	187555,528	180220,185	7335,3425
**EBNA1BP2**	11890,71	6413,33	14881,15	12067,93	5156,53	2827,1	11313,28	3991,815	7321,465
**MRPS12**	10794,48	4542,4	9817,52	13354,36	3578,12	1038,51	9627,19	2308,315	7318,875

**CTSW**	7074,36	11631,65	19965,3	674,06	6,07	5032,12	9836,3425	2519,095	7317,2475
**NOLC1**	24379,37	21978,61	13400	16041,93	11748,95	11573,08	18949,9775	11661,015	7288,9625
**DHX9**	37194,48	26067,98	22681,57	30196,49	17029,64	26509,63	29035,13	21769,635	7265,495
**CYC1**	15915,36	28164,31	11603,88	14091,62	17304,25	3129,11	17443,7925	10216,68	7227,1125
**ADAM15**	11457,23	7687,78	12915,9	13905,14	7818,46	760,43	11491,5125	4289,445	7202,0675
**POLD1**	12302,32	24761,64	16948,66	16567,33	9997,76	10910,32	17644,9875	10454,04	7190,9475
**TTLL12**	13130,31	18259,38	10560,81	14883,09	6016	8040,81	14208,3975	7028,405	7179,9925
**FLOT1**	5429,01	6688,32	17512,1	15722,96	5014,53	3306,2	11338,0975	4160,365	7177,7325
**WDR83OS**	4741,44	4651,49	29270,73	6202,43	6087,59	1996,42	11216,5225	4042,005	7174,5175
**LGALS9**	3728,51	3295,72	18103,38	6867,02	1480,94	206,52	7998,6575	843,73	7154,9275
**ALDH16A1**	7327,47	6060,04	14601,77	9508,23	1873,1	2593,63	9374,3775	2233,365	7141,0125
**FKBP1A**	19450,81	19190,51	18739,9	18773,59	11364,76	12442,79	19038,7025	11903,775	7134,9275
**HSH2D**	9639,8	15463,05	20810,27	9416,59	463,54	12933,6	13832,4275	6698,57	7133,8575
**FAM89A**	1811,2	11911,9	4024,07	11615,85	407,4	7,86	7340,755	207,63	7133,125
**MAP4K4**	18385,31	14970,95	8851,92	18726,85	4869,01	11332,5	15233,7575	8100,755	7133,0025
**RPN1**	29251,16	23326,22	26552,46	19798,38	14298,48	20963,37	24732,055	17630,925	7101,13
**EMILIN1**	429,65	29870,41	390,89	101	88,94	1121,27	7697,9875	605,105	7092,8825
**AP2A1**	7300,86	17729,18	12023,46	16216,63	5915,37	6592,46	13317,5325	6253,915	7063,6175
**SRM**	16115,32	10670,51	15337,49	16183,88	12239,07	2824,84	14576,8	7531,955	7044,845
**MDK**	796,11	1230,7	37903,22	324,88	205,76	5853,34	10063,7275	3029,55	7034,1775
**LAMTOR1**	17036,29	6396,22	13012,67	9380,03	4442,42	4421	11456,3025	4431,71	7024,5925
**RAN**	57741,32	36822,25	47887,59	32568,5	45414,4	28049,08	43754,915	36731,74	7023,175
**FP565260.1**	4242,71	2462,76	14635,06	15380,87	2195,78	2155,34	9180,35	2175,56	7004,79
**SLC39A3**	20358,62	18888,04	14628,57	7305,34	3216,54	13393,63	15295,1425	8305,085	6990,0575
**SSU72**	9721,57	10278,53	20141,24	17352	9209,75	5567,42	14373,335	7388,585	6984,75
**PIN1**	4785,45	5558,49	19358,93	14000,96	3416,14	4478,09	10925,9575	3947,115	6978,8425
**XK**	15458,36	6781,58	4843,22	849,64	42,46	1,75	6983,2	22,105	6961,095
**NME4**	14782,38	14182,71	13186,58	15037,54	11368,17	3309,92	14297,3025	7339,045	6958,2575
**SIGLEC6**	1251,51	2891,49	616,9	27984,86	3,04	2478,15	8186,19	1240,595	6945,595
**COL23A1**	7042,44	7074,65	12851,73	5652,53	2445,98	9,72	8155,3375	1227,85	6927,4875
**NME1**	23137,57	15394,32	14187,39	13970,18	13577,59	5937,38	16672,365	9757,485	6914,88
**YIPF2**	2398,43	3575,75	16948,05	14783,11	3198,33	1919,18	9426,335	2558,755	6867,58
**SAFB**	16110,25	15797,67	18132,94	22807,17	12467,64	10251,43	18212,0075	11359,535	6852,4725

**AHCY**	22370,99	27145,46	12260,16	22698,27	15783,81	12814,18	21118,72	14298,995	6819,725
**MINPP1**	10869,05	15475,09	2977,27	13184,34	1032,38	6648,06	10626,4375	3840,22	6786,2175
**TAF15**	10220,47	17942,13	18975,72	14826,09	6719,72	10700,77	15491,1025	8710,245	6780,8575
**SLC29A1**	11796,96	4781,38	5410,32	23882,05	6341,42	3055,1	11467,6775	4698,26	6769,4175
**LRRC59**	14244,7	15053,31	10130,93	18305,95	6362,15	8982,03	14433,7225	7672,09	6761,6325
**CCT7**	47598,29	35435,28	32325,85	40906,85	40249,04	24428,87	39066,5675	32338,955	6727,6125
**RHOG**	12717,39	9372,68	21982,92	15384,82	12445,07	3849,86	14864,4525	8147,465	6716,9875
**MARCKSL1**	8526,5	19183,05	29178,67	2537,66	7948,43	8363,23	14856,47	8155,83	6700,64
**PKLR**	1278,01	14983,98	807,51	9715,34	0	4,04	6696,21	2,02	6694,19
**MATK**	12712,6	12118,4	3803,8	1649,75	510,15	1247,97	7571,1375	879,06	6692,0775
**EHBP1L1**	5333,27	17236	9524,81	14266,68	5243,51	4578,01	11590,19	4910,76	6679,43
**SERPINH1**	13509,46	15316,89	10968,88	7918,21	4023,36	6508,34	11928,36	5265,85	6662,51
**MYO16**	6037,35	2,81	11373,75	10387,3	0	576	6950,3025	288	6662,3025
**GMPR**	5698,77	8740,62	9232,78	9144,59	127,37	2995,42	8204,19	1561,395	6642,795
**EIF4EBP1**	12875,49	8930,72	15139,41	10038,11	8723,49	1538,78	11745,9325	5131,135	6614,7975
**NUDC**	13858,21	10959,65	18174,46	17055,92	8598,33	8221,14	15012,06	8409,735	6602,325
**PLXND1**	13846,6	4556,71	21799,89	22379,11	12203,69	5915,54	15645,5775	9059,615	6585,9625
**RPL23**	58779,72	69325,53	107744,12	48415,73	104987,77	23979,24	71066,275	64483,505	6582,77
**RNF24**	13764,84	8346,35	8998,46	16067,19	3739,45	6711,99	11794,21	5225,72	6568,49
**PEBP1**	18674,14	16493,05	29151,12	11330,01	14887,84	9814,63	18912,08	12351,235	6560,845
**CPT1A**	7108,73	15395,84	10606,84	19999,27	322,06	13130,99	13277,67	6726,525	6551,145
**PPP1R14B**	13053,85	16409,17	20509,7	14626,35	13864,31	5341,04	16149,7675	9602,675	6547,0925
**SF3A1**	14112,19	17188,2	11962,18	16538,03	5921,7	10915,92	14950,15	8418,81	6531,34
**PSMC3**	18418,7	10817,42	11669,84	22354,8	9518,31	9053,72	15815,19	9286,015	6529,175
**NHP2**	11902,08	11611,24	11786,66	12842,69	6708,76	4335,06	12035,6675	5521,91	6513,7575
**JAK2**	1667,33	2063,97	1647,86	29080,24	1167,95	3048,82	8614,85	2108,385	6506,465
**CCND2**	11799,73	18741,65	18260,82	6,89	2,77	11404,01	12202,2725	5703,39	6498,8825
**PSMD13**	16195,36	8915,76	16919,18	14166,21	9192,73	5916,8	14049,1275	7554,765	6494,3625
**VPS37B**	8729,45	14712,84	13316,81	12853,56	3242,12	8613,52	12403,165	5927,82	6475,345
**ELAC2**	9339,65	11918,48	10839,99	8438,31	5255,63	2067,51	10134,1075	3661,57	6472,5375
**FAM171A1**	16367,51	1581,56	14006,32	10526,3	17,76	8278,63	10620,4225	4148,195	6472,2275
**NACC1**	7221,67	12000,07	8238,37	17115,4	4983,46	4384,36	11143,8775	4683,91	6459,9675
**CDC42BPA**	7138,28	6902,89	10107,62	6549,74	2429,16	9,3	7674,6325	1219,23	6455,4025

**F2RL3**	3576,77	4806,42	7902,12	11619,82	508,58	599,11	6976,2825	553,845	6422,4375
**INTS1**	12369,79	18455,48	13219,39	11860,56	5894,86	9242,9	13976,305	7568,88	6407,425
**HINT1**	21987,52	12403,98	21411,45	13177,43	7664,3	14062,71	17245,095	10863,505	6381,59
**MAP3K11**	11813,39	9355,45	13235,19	7812,18	4360,54	3985,67	10554,0525	4173,105	6380,9475
**CCND3**	15223,56	13930,2	22719,63	26177,03	12481,37	13784,01	19512,605	13132,69	6379,915
**RPS4X**	99463,71	122055,7	99708,34	73389,63	107910,39	76646,21	98654,335	92278,3	6376,035
**MFNG**	8471,19	12608,99	14578,63	14863,37	6699,68	5861,74	12630,545	6280,71	6349,835
**SLC25A37**	11504,98	7587,09	9845,28	17839,9	8805,15	1900,82	11694,3125	5352,985	6341,3275
**CD82**	2275,25	8848,6	17185,13	25116,73	264,43	13766,03	13356,4275	7015,23	6341,1975
**NOMO2**	20004,29	11181,03	24133,87	22036,94	17513,05	8514,5	19339,0325	13013,775	6325,2575
**NBEAL2**	15670,8	14902,46	15529,16	14874,27	6800,81	11108,35	15244,1725	8954,58	6289,5925
**ELOF1**	2702,85	3356,97	3617,5	23995,94	2817,3	1445,23	8418,315	2131,265	6287,05
**DDX5**	30889,66	52561,82	33814,6	35852,97	28073,06	35926,69	38279,7625	31999,875	6279,8875
**SLC16A1**	16178,9	9951,34	8947,7	15494,16	5023,74	7717,7	12643,025	6370,72	6272,305
**ILF2**	25705,89	20496,47	22482,98	28574,24	19905,25	16198,72	24314,895	18051,985	6262,91
**TESPA1**	11320,9	4720,97	5727,32	7978,29	23,19	2347,48	7436,87	1185,335	6251,535
**RRP1**	8798,66	5719,18	15373,93	9585,09	4918,88	2334,24	9869,215	3626,56	6242,655
**CDC42**	18974,32	16223,62	21508,15	29159,29	14680,63	15769,37	21466,345	15225	6241,345
**NT5DC2**	11705,95	7517,58	3868,34	7883,39	3007,57	6,65	7743,815	1507,11	6236,705
**WNT5B**	4446,32	4264,22	12057,6	4398,67	10,39	116,72	6291,7025	63,555	6228,1475
**H2AZ1**	45430,46	29587,57	23884,7	28986,9	22833,56	28663,74	31972,4075	25748,65	6223,7575
**FKBP4**	28272,03	15963,45	23115,89	17138,91	19928,76	9873,43	21122,57	14901,095	6221,475
**PSMF1**	15482,18	7689,89	12172,42	11231,48	4127,66	6742,04	11643,9925	5434,85	6209,1425
**EPN1**	8436,33	14239,17	15775,01	15394,43	9549,43	4984,83	13461,235	7267,13	6194,105
**NDUFA11**	8003,86	8419,73	12924,02	17938,59	6532,61	4737,03	11821,55	5634,82	6186,73
**ELAVL1**	13451,19	10676,06	7992,01	22015,04	6030,04	8674,4	13533,575	7352,22	6181,355
**TOR4A**	7585,1	7369,97	11078,6	12511,63	4155,33	2763,86	9636,325	3459,595	6176,73
**KIF13A**	4004,93	1244,59	10116,81	9352,96	29,73	0	6179,8225	14,865	6164,9575
**CXXC5**	5311,46	22140,29	5296,84	11575,68	735,02	9137,85	11081,0675	4936,435	6144,6325
**ATP6AP1**	9918,05	7922,89	20704,29	12201,7	7495,64	5599,82	12686,7325	6547,73	6139,0025
**APEH**	15239,92	14952,33	20088,28	10218,19	7674,22	10299,5	15124,68	8986,86	6137,82
**SNRPB**	46539,75	29038,02	24123,26	26098,35	28256,11	22409,98	31449,845	25333,045	6116,8
**ISOC2**	5133,27	8900,78	14076,41	10615,94	4761,47	2377,98	9681,6	3569,725	6111,875

**KCNN4**	6408,78	5528,72	18739,44	10220,02	5433,6	2834,97	10224,24	4134,285	6089,955
**CFP**	9825,38	1564,84	11434,06	5841,38	705,5	1462,48	7166,415	1083,99	6082,425
**GARS1**	15373,75	12308,14	19178,9	20012,03	8014,06	13258,01	16718,205	10636,035	6082,17
**DCAF15**	5740,73	4347,03	18491,16	11844,06	3939,36	4148,18	10105,745	4043,77	6061,975
**HMGN1**	48202,36	24458,02	37698,49	39792,77	40506,06	22453,03	37537,91	31479,545	6058,365
**BCL2L1**	8043,49	14307,99	11174,72	14612,35	1422,05	10551,25	12034,6375	5986,65	6047,9875
**PRF1**	5380,94	12529,75	7389,41	612,07	9,93	886,13	6478,0425	448,03	6030,0125
**RBM42**	7839,03	7419,99	19116,76	11104,55	6131,2	4549,73	11370,0825	5340,465	6029,6175
**NR1H2**	4051,47	5689,1	19589,74	9555,21	4917,1	2502,33	9721,38	3709,715	6011,665
**XPO5**	14506,47	8671,71	12787,35	17554,4	6635,34	8109,4	13379,9825	7372,37	6007,6125
**NTRK1**	4830,44	11275,95	7453,94	7132,7	6,8	3339,68	7673,2575	1673,24	6000,0175
**CLTC**	25308,57	23091,24	20993,88	36239,56	29098,86	11728,79	26408,3125	20413,825	5994,4875
**NAPA**	5736,88	6626,65	19425,89	7837,49	3150,75	4682,29	9906,7275	3916,52	5990,2075
**MFN2**	9377,36	9456,48	11441,78	21629,51	7912,75	6064,18	12976,2825	6988,465	5987,8175
**ICAM1**	705,89	1225,94	21049,72	2562,34	425,76	380,76	6385,9725	403,26	5982,7125
**TRA2B**	20691,48	27967,92	13501,34	14873,68	12858,96	13696,7	19258,605	13277,83	5980,775
**IER2**	9261,82	12213,16	29863,9	19409,18	14968,92	8452,61	17687,015	11710,765	5976,25
**TRIM58**	8629,92	5367,94	2038,67	7851,56	0	19,22	5972,0225	9,61	5962,4125
**UPF1**	14015,2	15314,25	10540,12	15976,88	5937,87	10109,64	13961,6125	8023,755	5937,8575
**LMAN2**	11722,4	11116,89	15759,68	14700,25	8387,96	6391,16	13324,805	7389,56	5935,245
**PRDX6**	31555,81	11709,73	22418,01	20883,1	22152,44	9260,63	21641,6625	15706,535	5935,1275
**NDUFV3**	6396,74	4205,35	18811,51	6357,59	4182,66	1838,72	8942,7975	3010,69	5932,1075
**SLC25A3**	31521,57	34840,98	30075,84	26085,37	34304,41	15108,45	30630,94	24706,43	5924,51
**PRDX1**	16001,74	17334,95	13752,1	19673,5	10863,78	10668,69	16690,5725	10766,235	5924,3375
**CDC20**	14949,23	8416,69	18012,11	10917,08	8189,67	6110,31	13073,7775	7149,99	5923,7875
**ITM2A**	9069,24	22427,22	8735,77	8147,95	0,91	12352,38	12095,045	6176,645	5918,4
**STAT3**	7111,22	14820,47	22552,95	10172,55	3494,51	12018,69	13664,2975	7756,6	5907,6975
**ATP5MG**	15300,39	7907,27	19508,03	14632,75	10453,95	6425,21	14337,11	8439,58	5897,53
**NME2**	35285,16	31754,75	45497,6	32484,56	48364,37	12359,16	36255,5175	30361,765	5893,7525
**DPP3**	9533,35	11149,52	6130,89	7201,38	2404,97	2829,72	8503,785	2617,345	5886,44
**MT-CYB**	109437,2	187694,4	164130,27	96287,11	88796,76	178239,7	139387,258	133518,215	5869,0425
**HDLBP**	32662,33	16603,27	28643,25	39649,16	20108,39	26933,98	29389,5025	23521,185	5868,3175
**MUC1**	335,06	6859,8	16073,05	3321,2	712,04	891,1	6647,2775	801,57	5845,7075

**DHCR24**	19404,74	11891,01	4502,49	8650,57	10541,81	2,08	11112,2025	5271,945	5840,2575
**IL1B**	8862,41	2042,97	14137,64	6881,01	2440,34	1863,89	7981,0075	2152,115	5828,8925
**KCNK5**	7796,36	9193,23	10138,24	8048,5	824,74	5149,36	8794,0825	2987,05	5807,0325
**SSR4**	10117,97	7001,26	18786,61	11550,94	8049,96	4096,35	11864,195	6073,155	5791,04
**GPAA1**	9164,51	22419,5	8615,86	8364,35	9177,71	3558,38	12141,055	6368,045	5773,01
**PDXK**	7402,67	8278,16	15975,39	18263,15	6584,63	6861,37	12479,8425	6723	5756,8425
**TIMM50**	10409,81	8546,03	13921,67	10303,43	6096,27	3981,42	10795,235	5038,845	5756,39
**SSBP3**	6432,04	8666,38	12882,19	9688,07	563,47	6758,96	9417,17	3661,215	5755,955
**ADAMTS14**	5791,75	6196,99	13280,87	4143,04	20,19	3205,04	7353,1625	1612,615	5740,5475
**TPST2**	3893,99	21955,85	4949,89	3930,23	1451,74	4446,77	8682,49	2949,255	5733,235
**AK3**	2178,26	3365,65	1775,77	28921,1	1026,63	5649,08	9060,195	3337,855	5722,34
**ADRM1**	12670,53	8990,53	11873,65	13908,4	5984,86	6300,03	11860,7775	6142,445	5718,3325
**HNRNPA3**	51802,09	42794,17	34338,96	41445,91	53189,95	20567,52	42595,2825	36878,735	5716,5475
**STARD8**	5980,47	6399,92	4452,99	7668,61	802,05	16,61	6125,4975	409,33	5716,1675
**CAPN15**	5335,49	13145,86	7035,26	10159,52	4390,62	2022,28	8919,0325	3206,45	5712,5825
**MCM7**	41647,82	44269,83	31861,73	23591,2	29807,4	29467,18	35342,645	29637,29	5705,355
**ANKLE1**	5513,61	6199,23	3561,6	11684,28	1969,99	102,48	6739,68	1036,235	5703,445
**GALNT2**	12893,17	20806,41	13545,21	17998,83	3812,97	17409,78	16310,905	10611,375	5699,53
**CASP3**	4122,26	13010,2	5337,2	10302,3	1723,67	3264,68	8192,99	2494,175	5698,815
**PSMB2**	16215,85	9553,03	12467,06	13459,13	6267,31	8196,31	12923,7675	7231,81	5691,9575
**RAB1B**	18194,03	14124,9	17398,31	13824,89	10771,77	9620,24	15885,5325	10196,005	5689,5275
**CLCN4**	12441,55	3968,49	3329,41	3023,98	15,64	4,54	5690,8575	10,09	5680,7675
**GRK6**	9790,21	7819,31	7335,92	11084,94	3258,14	3443,25	9007,595	3350,695	5656,9
**PRPF31**	8093,34	11442,78	12582,35	8460,64	4244,22	4784,13	10144,7775	4514,175	5630,6025
**PLTP**	5654,04	4000,35	13588,68	18753,1	5983,88	3780,37	10499,0425	4882,125	5616,9175
**SFPQ**	33152,46	36444,57	46868,09	42382,84	32423,55	35790,21	39711,99	34106,88	5605,11
**CORO1C**	15355,36	17642,82	12520,32	11387,13	6491,62	10761,42	14226,4075	8626,52	5599,8875
**PUF60**	13976,42	22839,91	12858,54	8761,1	14864,96	3163,09	14608,9925	9014,025	5594,9675
**ATF5**	3220	6211,86	9712,19	10218,53	1197,61	2300,72	7340,645	1749,165	5591,48
**CHID1**	8388,36	7210,05	12001,52	6960,28	3719,88	2380,38	8640,0525	3050,13	5589,9225
**STK40**	5797,18	7763,74	12359,94	7786,64	1566,4	4115,81	8426,875	2841,105	5585,77
**CNRIP1**	8872,37	12097,35	13811,72	9039,39	56,57	10706,83	10955,2075	5381,7	5573,5075
**PSIP1**	6245,31	9993,71	7182,08	62600,55	8982,11	22922,62	21505,4125	15952,365	5553,0475

**TMED9**	12381,36	10096,18	15730,81	16742,94	8756,91	7652,82	13737,8225	8204,865	5532,9575
**MAT2A**	22685,61	29434,72	34340	20041,29	16758,18	25438,91	26625,405	21098,545	5526,86
**PTGS1**	17252,94	25457,67	8843,33	6769,78	1494,39	16631,89	14580,93	9063,14	5517,79
**NUP98**	12909,53	11711,67	18004,29	15928,46	8569,49	9685,25	14638,4875	9127,37	5511,1175
**TXN2**	7685,34	16504,38	14554,55	11611,73	9650,15	4513,38	12589	7081,765	5507,235
**ARFGAP1**	6635,65	5824,5	19184,97	12458,57	4906,17	6177,77	11025,9225	5541,97	5483,9525
**FAM32A**	6247,26	8305,44	19705,31	15857,34	8345,54	5763,82	12528,8375	7054,68	5474,1575
**PPIF**	19659,53	22044,4	11045,27	9692,93	8079,02	12206,14	15610,5325	10142,58	5467,9525
**B2M**	56876	51404,9	129451,07	16171,21	63130,28	52893,7	63475,795	58011,99	5463,805
**CD320**	5903,49	4442,85	13974,91	10411,18	4050,4	2408,15	8683,1075	3229,275	5453,8325
**CMBL**	5108,3	11650,14	9226,35	8700,07	133,69	6304,54	8671,215	3219,115	5452,1
**NASP**	22652,3	26041,34	20867,64	21692,59	16667,09	18066,78	22813,4675	17366,935	5446,5325
**OS9**	11738,89	12816,35	31816,67	13769,21	15268,4	8943,18	17535,28	12105,79	5429,49
**SF1**	20744,09	27309,71	30010,95	20112,75	21582,81	16651,12	24544,375	19116,965	5427,41
**RANGAP1**	13699,07	14292,5	13635,46	17268,15	10378,88	8244,81	14723,795	9311,845	5411,95
**HSPE1**	14850,59	9893,46	10656,62	13300,39	10604,96	2924,26	12175,265	6764,61	5410,655
**TMEM109**	14224,71	10754,73	8650,23	8383,52	4857,94	5330,9	10503,2975	5094,42	5408,8775
**SNAPC3**	1249,76	2451,86	1119,65	29967,27	3052,35	3548,2	8697,135	3300,275	5396,86
**SLC38A5**	18641,43	382,41	3893,48	8272,29	3206,61	1611,74	7797,4025	2409,175	5388,2275
**TRIM27**	9327,99	7238,98	10677,9	14955,6	6257,02	4069,01	10550,1175	5163,015	5387,1025
**NOP58**	20355,55	12998,44	17188,59	15421,98	14199,74	8033,71	16491,14	11116,725	5374,415
**TXLNA**	8416,49	8667,21	13265,84	10192,39	4953,6	4569,68	10135,4825	4761,64	5373,8425
**GPR85**	11975,46	2214,89	4092,72	3363,85	71,96	4,38	5411,73	38,17	5373,56
**SSRP1**	34978,89	24831,84	25124,02	29934,58	28052,67	18652,48	28717,3325	23352,575	5364,7575
**ARHGEF12**	4813,67	5364,41	3920,8	9733,94	1117,35	75,5	5958,205	596,425	5361,78
**PLEC**	3571,49	38067,44	2582,34	13193,68	10769,17	7219,54	14353,7375	8994,355	5359,3825
**APOBEC3C**	11371,54	11904,42	10812,51	8447,91	4683,66	5869,4	10634,095	5276,53	5357,565
**RAB11A**	12598,29	8358,22	20571,91	11218,32	6536,15	9123,31	13186,685	7829,73	5356,955
**MLXIP**	6393,95	16222,7	15740,64	12225,74	4350,65	10234,21	12645,7575	7292,43	5353,3275
**ERGIC3**	11651,26	12624,84	19826,06	26248,9	13743,98	10726,32	17587,765	12235,15	5352,615
**PSMC4**	12246,54	6087,12	12342,67	11222,27	5713,29	4553,12	10474,65	5133,205	5341,445
**GDF11**	2267,94	16283,46	6305,48	10400,56	2101,04	4872,79	8814,36	3486,915	5327,445
**BMP2K**	11732,82	18649,87	6382,51	14725,35	1154,83	13941,01	12872,6375	7547,92	5324,7175

**DDAH2**	1713,48	3979	17042,88	5880,55	1959,46	1709,12	7153,9775	1834,29	5319,6875
**RPS8**	115680,7	100130,7	170393,18	101377,2	193148,67	40008,08	121895,44	116578,375	5317,065
**JPT2**	8492,19	11749,93	5268,42	10946,81	2672,2	4931,26	9114,3375	3801,73	5312,6075
**NDUFC2**	11132,03	6671,51	13902,33	11120,5	6982,62	3817,37	10706,5925	5399,995	5306,5975
**MCM5**	17233,02	21218,58	12612,98	16579,52	6843,67	16374,02	16911,025	11608,845	5302,18
**SNRPC**	9997,23	6798,24	12763,24	8718,02	5523,63	3010,58	9569,1825	4267,105	5302,0775
**PTOV1**	8382,33	13466,86	15546,15	10901,1	7728,14	5822,79	12074,11	6775,465	5298,645
**GCSAML**	13833,3	6800,97	3218,55	7932,22	18,84	5297,65	7946,26	2658,245	5288,015
**POLR1A**	8989,83	10477,79	11415,55	10200,51	3524,07	6450,96	10270,92	4987,515	5283,405
**DPP9**	5667,39	7256,91	5024,53	15162,94	2810,8	3180,25	8277,9425	2995,525	5282,4175
**SPTA1**	3513,67	3750,61	10727,8	4545,28	0	706,04	5634,34	353,02	5281,32
**QPRT**	8481,81	8574,73	10730,75	5637,57	375	5812,5	8356,215	3093,75	5262,465
**UQCRFS1**	10525,09	9679,78	19326,95	12140,26	9566,3	5756,66	12918,02	7661,48	5256,54
**AQP3**	908,74	3689,38	2190,84	14411,95	62,78	41,33	5300,2275	52,055	5248,1725
**PCMT1**	11064	8495,9	10163,23	20437,58	5593,24	8991,87	12540,1775	7292,555	5247,6225
**MLEC**	16243,25	14739,12	19655	20894,42	8844,78	16436,14	17882,9475	12640,46	5242,4875
**APOC2**	8220,92	9435,54	3939,57	534,02	345,8	235,92	5532,5125	290,86	5241,6525
**EPHB4**	3857	5530,62	7418,51	9774,51	902,99	1919,27	6645,16	1411,13	5234,03
**ETS2**	14902,93	13290,44	10585,01	22927,66	10352,32	10043,72	15426,51	10198,02	5228,49
**BLVRA**	7675,79	1959,91	8840,98	10741,39	1770,57	2391,79	7304,5175	2081,18	5223,3375
**POLR2E**	20951,11	17182,89	13353,93	9045,69	12568,91	7254,43	15133,405	9911,67	5221,735
**TMC6**	10985,15	12563,74	13114,94	29287,89	15919,86	6617,46	16487,93	11268,66	5219,27
**TMEM14C**	8285,88	5667,31	17318,94	6493,21	6029,82	2427,95	9441,335	4228,885	5212,45
**SLC39A7**	7277,5	6765,5	13513,91	7069,12	4480,67	2410,63	8656,5075	3445,65	5210,8575
**ACAP1**	2749,9	11703,28	14313,8	7333,44	2018,95	5618,56	9025,105	3818,755	5206,35
**RBBP4**	24244,41	23109,27	20816,92	23600,04	12358,46	23116,81	22942,66	17737,635	5205,025
**CHD3**	7513,41	21479,2	24816,65	17659,84	15583,95	9744,67	17867,275	12664,31	5202,965
**SLC19A1**	8049,88	5117,73	8662,34	11414,72	3367,85	2852,13	8311,1675	3109,99	5201,1775
**FURIN**	9015,61	16513,63	15772,91	8807,71	2510,66	12144,91	12527,465	7327,785	5199,68
**ATP6V0A1**	3370,91	4519,95	6850,06	7995,7	699,19	277,78	5684,155	488,485	5195,67
**TRAP1**	12402,93	17265,42	9789,13	15911,67	15562,78	1783,98	13842,2875	8673,38	5168,9075
**SNRNP200**	28495,23	29167,21	28803,29	27670,71	13936,32	32802,87	28534,11	23369,595	5164,515
**EGLN2**	6310,42	7776,78	16320,13	10742,67	5428,26	4844,03	10287,5	5136,145	5151,355

**STAT5A**	24859,62	29001,14	51888,41	24304,59	174,88	54550,83	32513,44	27362,855	5150,585
**PAICS**	36495,73	29175,76	13575,69	30642	21364,23	23309,79	27472,295	22337,01	5135,285
**CUTA**	8694,1	7705,86	14146,34	6142,19	5752,1	2327,71	9172,1225	4039,905	5132,2175
**KARS1**	20678,62	15635,39	14339,13	15382,82	18718,31	4058,57	16508,99	11388,44	5120,55
**MICALL2**	4767,04	8460,1	5052,5	7160,94	1834,1	656,44	6360,145	1245,27	5114,875
**RPL35**	48577,77	50863,3	59090,16	43254,24	68670,01	22013,97	50446,3675	45341,99	5104,3775
**AKT1S1**	4854,1	6973,69	12015,23	6992,02	2996,37	2212,78	7708,76	2604,575	5104,185
**UBAP2L**	9674,92	15155,99	16857,02	19915,76	10031,22	10567,95	15400,9225	10299,585	5101,3375
**MORC3**	5052,44	2649,84	17978,53	9253,57	5421,22	1853,93	8733,595	3637,575	5096,02
**CSNK2B**	9735,01	8540,59	13298,21	12379,77	9154,43	2643,2	10988,395	5898,815	5089,58
**CDK4**	21197,62	18976,49	16637,34	16407,48	10515,56	15923,65	18304,7325	13219,605	5085,1275
**ADSL**	13543,15	13859,52	7680,79	14079,48	8296,33	6139,19	12290,735	7217,76	5072,975
**PSMG1**	10568,01	4367,45	4985,52	16583,19	3292,61	4831,27	9126,0425	4061,94	5064,1025
**HNRNPA2B1**	103762,3	106191,7	94109,43	78058,34	67493,32	113442,3	95530,4375	90467,785	5062,6525
**ICAM2**	5641,9	6812,13	7929,96	5408,36	322,76	2471,73	6448,0875	1397,245	5050,8425
**SUPT16H**	26693,52	25592,09	14536,65	23273,84	14425,42	20532,94	22524,025	17479,18	5044,845
**TIMM44**	4739,31	4861,3	7163,64	13893,28	3097,24	2153,66	7664,3825	2625,45	5038,9325
**PLVAP**	507,16	2367,48	1735,24	15561,56	0	13	5042,86	6,5	5036,36
**NET1**	12446,84	12028,47	8624,35	23806,62	7962,09	10420,72	14226,57	9191,405	5035,165
**PRSS21**	8610,85	5702,78	7814,21	0	1,08	995,44	5531,96	498,26	5033,7
**MRPL37**	9738,17	10477,2	18868,98	15556,96	11417,49	5853,93	13660,3275	8635,71	5024,6175
**C1QBP**	13470,17	21356,02	19041,08	16388,73	21729,83	3352,59	17564	12541,21	5022,79
**ARHGEF6**	11635,18	10226,07	12484,87	24854,93	10285,89	9269,56	14800,2625	9777,725	5022,5375
**LIG1**	10515,29	12844,26	10802,31	10452,77	5127,54	7137,08	11153,6575	6132,31	5021,3475
**CS**	16736,29	18309,23	15519,99	15476,36	12977,06	10009,31	16510,4675	11493,185	5017,2825
**UHRF1**	9237,11	14024,96	4964,34	23386,88	4701,97	11070,2	12903,3225	7886,085	5017,2375
**SYNCRIP**	23765,58	23611,97	21458,62	30437,64	19359,61	20250,2	24818,4525	19804,905	5013,5475
**GTF2F1**	6675,66	8607,48	8515,92	20069,12	6224,47	5691,67	10967,045	5958,07	5008,975
**ANKRD27**	12881,12	12871,11	19850,29	18429,69	3565,72	18452,43	16008,0525	11009,075	4998,9775
**CHERP**	9246,78	10455,29	12230,19	12878,93	6067,91	6348,21	11202,7975	6208,06	4994,7375
**LEPR**	5307,73	5192,71	3639,15	11216,2	224,09	2477,18	6338,9475	1350,635	4988,3125
**SART1**	13939,87	8862,68	10872,21	11115,96	8291,73	4132,45	11197,68	6212,09	4985,59
**NOSIP**	7485,36	6733,94	12508,44	8289,02	4101,48	3440	8754,19	3770,74	4983,45

**DDX54**	12751,56	13890,6	13863,22	12714,87	11763,37	4889,69	13305,0625	8326,53	4978,5325
**EIF5B**	26423,99	19565,58	33021,81	26773,83	30514,07	12463,18	26446,3025	21488,625	4957,6775
**PPP1R14A**	2277,84	2890,11	12110,75	2485,18	0	0,93	4940,97	0,465	4940,505
**PSMA7**	23840,39	15685,59	21882,9	24750,76	16298,58	16924,36	21539,91	16611,47	4928,44
**PMPCA**	7504,82	11607,81	9901	9019,43	5108,21	4072,3	9508,265	4590,255	4918,01
**TCOF1**	15672,54	14586,2	9943,57	11870,63	10511,14	5708,64	13018,235	8109,89	4908,345
**LASP1**	11913,2	17474,73	15945,52	20886,52	14061,95	9256,13	16554,9925	11659,04	4895,9525
**WDR46**	7147,77	6834,54	10026,39	7968,98	4637,66	1575,17	7994,42	3106,415	4888,005
**MRPS34**	10859,7	9402,47	10081,47	9469,88	8894,1	1267,75	9953,38	5080,925	4872,455
**SF3B5**	6455,42	8034,55	9927,95	11711,51	4354,07	3990,69	9032,3575	4172,38	4859,9775
**IPO4**	8890,74	13833,93	5359,15	8850,28	5962,36	2792,21	9233,525	4377,285	4856,24
**TRIO**	4741,34	3317,5	13922,14	3110,51	2829,81	10,01	6272,8725	1419,91	4852,9625
**EIF3A**	24646,89	39774,31	19585,7	29707,48	22862,46	24289,72	28428,595	23576,09	4852,505
**FAH**	6888,01	2597,85	8555,15	6334,73	1277,41	1217,83	6093,935	1247,62	4846,315
**CAPZB**	18529,98	12927,26	25126,5	22159,74	17453,86	12232,94	19685,87	14843,4	4842,47
**PYCR2**	19763,72	7555,66	10979,06	8317,3	8490,07	5147,9	11653,935	6818,985	4834,95
**FAM234A**	5522,63	7101,68	12827,31	4501,74	2566,32	2748,49	7488,34	2657,405	4830,935
**SND1**	20883,69	20954,21	30333,09	20656,67	22892,47	13905,96	23206,915	18399,215	4807,7
**GMFG**	9085,03	8034,97	41406,73	14654,42	17185,83	9791,03	18295,2875	13488,43	4806,8575
**GALNT10**	9543,76	2985,75	6423,13	12891,18	655,48	5659,22	7960,955	3157,35	4803,605
**SCAF1**	7197,6	13699,69	12658,13	9584,46	5421,65	6562,18	10784,97	5991,915	4793,055
**SHKBP1**	6219,1	7543,58	20764,41	11684,92	7110,01	6412,74	11553,0025	6761,375	4791,6275
**GRWD1**	7987,86	7062,05	6532,84	7391,27	2763,09	2144,14	7243,505	2453,615	4789,89
**ERP29**	11249,69	15191,14	18132,7	8076,96	12361,98	4388,73	13162,6225	8375,355	4787,2675
**PARK7**	22108	15352,16	23742,55	19059,14	20224,48	10359,17	20065,4625	15291,825	4773,6375
**NUP153**	10166,07	12220,27	7608,82	11476,52	5160,43	6029,05	10367,92	5594,74	4773,18
**THRAP3**	17902,86	18540,48	18131,64	14501,17	11444,7	13605,35	17269,0375	12525,025	4744,0125
**ECPAS**	12544,19	9971,58	14706,72	10880,54	11959,66	2617,65	12025,7575	7288,655	4737,1025
**RNF10**	11034,75	11988,78	22731,75	14976,92	10134,98	10757,79	15183,05	10446,385	4736,665
**TRMT1**	6270,99	6511,21	8260,96	9847,16	4353,49	1625,44	7722,58	2989,465	4733,115
**RIN3**	7389,04	13140,74	4073,68	6886,12	1896,6	4421,63	7872,395	3159,115	4713,28
**HSPA9**	45938,42	32894,37	17475,34	28659,22	27965,68	25099,48	31241,8375	26532,58	4709,2575
**ZNF266**	2939,71	2358,88	11721,25	11583,9	2640,15	2259,03	7150,935	2449,59	4701,345

**FAM118A**	3068,29	3897,14	11433,35	9186,87	1183,33	3227,3	6896,4125	2205,315	4691,0975
**USP22**	17457,09	22989,39	20607,64	15689,84	15123,11	13877,7	19185,99	14500,405	4685,585
**CHD7**	9803,63	7179,84	7750,97	6073,05	4321,71	1717,37	7701,8725	3019,54	4682,3325
**EIF6**	9074,53	8765,82	14052,67	19600,01	9574,27	6808,54	12873,2575	8191,405	4681,8525
**ENSA**	18350,49	18881,45	26259,47	18310,87	18566,94	12973,42	20450,57	15770,18	4680,39
**ARGLU1**	9999,14	9232,46	13345,93	5758,41	4839,16	4971,98	9583,985	4905,57	4678,415
**NFATC3**	11001,11	13737,57	8899,99	9837,63	7118,07	5267,74	10869,075	6192,905	4676,17
**THBS1**	15280,86	179,81	3206,44	62,87	12,62	20,34	4682,495	16,48	4666,015
**LRP5**	5692,52	6471,76	11283,6	7532,79	1686,66	4483,33	7745,1675	3084,995	4660,1725
**SH3GL1**	4130,88	5493,27	5131,99	18170,08	3521,05	3640,61	8231,555	3580,83	4650,725
**PTK2**	3439,15	11862,23	2968,55	3088,71	1366,06	12,03	5339,66	689,045	4650,615
**ATF6B**	7372,2	9657,31	13681,74	8123,91	6937,95	3179,69	9708,79	5058,82	4649,97
**NCLN**	14894,16	14914,89	7387,02	7559,03	7935,27	5147,85	11188,775	6541,56	4647,215
**CHAF1A**	7459,64	9460,51	5674,19	19501,65	4719,59	7042,71	10523,9975	5881,15	4642,8475
**SLC39A4**	4193,03	8434,91	4821,91	2290,26	332,2	275,4	4935,0275	303,8	4631,2275
**NADK**	7688,31	9324,89	10112,69	10029,29	4238,98	5085,39	9288,795	4662,185	4626,61
**TLNRD1**	2770,27	9395,4	8048,15	8365,84	1019,86	4041,37	7144,915	2530,615	4614,3
**TMED3**	8595,15	7272,18	15376,02	7802,55	6558,83	3743,95	9761,475	5151,39	4610,085
**CHST2**	9401,6	1820,24	9745,2	5276,76	1663,94	2250,06	6560,95	1957	4603,95
**AP2B1**	15385,91	18562,6	17655,01	22524,63	13390,31	14467,46	18532,0375	13928,885	4603,1525
**URB1**	7275,72	7648,33	5750,51	9964,97	3213,16	2905,42	7659,8825	3059,29	4600,5925
**PBX2**	8418,66	8858,3	11292,57	10357,71	5756,94	4523,71	9731,81	5140,325	4591,485
**PTGER3**	2152,9	2415,4	9605,88	14038,68	4931,96	4,4	7053,215	2468,18	4585,035
**CDK11B**	9564,65	5528,92	13633,96	8337,72	4085,56	5289,88	9266,3125	4687,72	4578,5925
**ARL2**	7147,38	5408,43	12674,69	7691,42	4395,24	2911,03	8230,48	3653,135	4577,345
**DYRK1A**	8230,8	4690,07	22298,4	10175,32	5958,56	7585,85	11348,6475	6772,205	4576,4425
**PSMB4**	23783,32	14893,08	21679,68	24569,74	25006,07	8316,05	21231,455	16661,06	4570,395
**CLPTM1**	6977,52	6847	15982,89	10519,21	6990,31	4041,62	10081,655	5515,965	4565,69
**TRMT112**	12992,49	8317,58	13129,95	12108,78	10491,1	3655,98	11637,2	7073,54	4563,66
**UBE2L6**	5596,55	6163,44	13227,96	6777,09	795,13	5974,61	7941,26	3384,87	4556,39
**ATP13A2**	9220,74	5576,1	9390,25	7175,87	1640,88	4936,35	7840,74	3288,615	4552,125
**IRAK1**	15006,37	15658,3	15379,66	27638,54	14145,13	13600,16	18420,7175	13872,645	4548,0725
**PDCD11**	12624,14	13215,92	8599,84	9707,69	6113	6888,75	11036,8975	6500,875	4536,0225

**MYDGF**	7456,57	5954,58	13101,24	13202,76	8249,86	2537,31	9928,7875	5393,585	4535,2025
**PTP4A2**	18504,32	17638,16	16253,31	22613,29	16567,81	11886,89	18752,27	14227,35	4524,92
**GOLIM4**	6066,3	7818,24	2995,78	5246,85	1919,09	95,88	5531,7925	1007,485	4524,3075
**PRDX4**	14798,52	5029,27	5749,72	5036,38	2295,88	3969,55	7653,4725	3132,715	4520,7575
**FHL2**	5723,38	8273,82	3616,63	6353,5	83,84	2870,72	5991,8325	1477,28	4514,5525
**HSPBP1**	5740,38	10017,25	8373,31	6618,55	3972,52	2383,43	7687,3725	3177,975	4509,3975
**PDIA4**	23350,22	13515,32	17370,76	11404,9	13853,23	9954,39	16410,3	11903,81	4506,49
**IFRD2**	12398,61	9748,52	7810,4	7022,83	6910,34	2576,99	9245,09	4743,665	4501,425
**BACE2**	291,11	1734,49	23390,19	18,39	1,14	3737,69	6358,545	1869,415	4489,13
**APOL4**	1254,33	8944,4	1266,85	6483,81	0	0	4487,3475	0	4487,3475
**TMED1**	3334,9	2942,88	11696,44	8405,06	3025,66	1190,08	6594,82	2107,87	4486,95
**AFDN**	106,82	102,26	21,01	22229,96	2267,06	1,35	5615,0125	1134,205	4480,8075
**MAF1**	7090,8	21886,37	9556,84	6239,51	11463,7	1995,94	11193,38	6729,82	4463,56
**TSC22D1**	6626,38	8025,46	7223,36	3826,59	7,89	3922,48	6425,4475	1965,185	4460,2625
**AMMECR1**	9075,73	4920,08	7350,51	3892,39	1452,51	2275,77	6309,6775	1864,14	4445,5375
**YWHAG**	25095,97	22617,6	19334,29	21147,56	18553,56	16665,3	22048,855	17609,43	4439,425
**KLHDC3**	12607,09	14657,1	19564,16	21733,83	8888,96	16523,9	17140,545	12706,43	4434,115
**UBE2S**	15358,37	25204,68	15523,85	18012,67	15506,17	12683,35	18524,8925	14094,76	4430,1325
**NDUFB11**	16646,42	7841,61	12232,74	6726,58	8799,05	4066,46	10861,8375	6432,755	4429,0825
**CCAR2**	11175,99	14121,65	8628,5	9246,26	7293,38	5442,04	10793,1	6367,71	4425,39
**PRMT2**	4962,77	4678,68	16848,61	7783,11	4489,73	3803	8568,2925	4146,365	4421,9275
**SCAF4**	8772,86	5251,49	14252,5	7168,27	3863,75	5018,41	8861,28	4441,08	4420,2
**ELOVL1**	6501,63	3605,12	15816,11	6479,46	3740,42	3630,74	8100,58	3685,58	4415
**SSR1**	13149,25	11027,56	12297,18	16712,33	10452,65	7312,54	13296,58	8882,595	4413,985
**GTPBP4**	10516,44	9169,22	14433,12	10769,84	7922,11	5699,76	11222,155	6810,935	4411,22
**SZRD1**	11463,62	13767,64	13066,85	14004,18	8584,34	8758,44	13075,5725	8671,39	4404,1825
**HDAC2**	18308,9	13244,21	19182,33	27108,6	11295,56	18843,1	19461,01	15069,33	4391,68
**UQCRH**	25188,77	18538,9	33865,47	26746,3	34929,11	8463,91	26084,86	21696,51	4388,35
**DHRS3**	3565,34	3504,66	10868,87	2681,33	264,99	1275,07	5155,05	770,03	4385,02
**CYB5R3**	11828,17	10505,19	13635,82	6536,74	5595,18	6899,97	10626,48	6247,575	4378,905
**PNP**	10105,17	8569,66	7685,01	11443,32	6170,17	3976,03	9450,79	5073,1	4377,69
**PRKCB**	8352,12	18025,43	7100	39083,04	14625,01	12902,22	18140,1475	13763,615	4376,5325
**CSTB**	7636,82	3142,35	15226,02	7113,72	5666,37	2144,07	8279,7275	3905,22	4374,5075

**ATP5MC3**	20008,87	16350,23	16081,53	14974,73	20537,41	4423	16853,84	12480,205	4373,635
**BOP1**	14209,52	26887,77	9260,33	10642,8	20205,76	1547,24	15250,105	10876,5	4373,605
**CDK16**	14957,75	5655,78	5576	6729,56	4341,79	3407,68	8229,7725	3874,735	4355,0375
**C19orf53**	3974,35	4757,24	18118,55	6318,67	5829,44	2070,38	8292,2025	3949,91	4342,2925
**TWF2**	8921,39	8151,71	8239,72	7339,17	4690,1	2952,89	8162,9975	3821,495	4341,5025
**RPL10A**	71609,84	72900,12	73916,43	50738,61	99771,36	26128,65	67291,25	62950,005	4341,245
**PEAR1**	3994,3	1160,17	2336,5	10121,47	40,93	125,65	4403,11	83,29	4319,82
**TPSAB1**	871,7	15450,73	643,85	2765,17	23,88	1210,74	4932,8625	617,31	4315,5525
**MDN1**	27050,56	11750,18	28189,1	18764,59	27962,01	6285,26	21438,6075	17123,635	4314,9725
**TK1**	16215	12089,48	13574,16	11590,67	8369,47	9735,81	13367,3275	9052,64	4314,6875
**PPP1R26**	4137,55	5951,98	1951,49	6770,49	800,98	0	4702,8775	400,49	4302,3875
**PUM3**	7803,4	4430,78	4199,63	19721,93	7429,19	2045,14	9038,935	4737,165	4301,77
**NDUFV1**	16257,36	13070,65	14503,34	18177,62	14276,22	8133,77	15502,2425	11204,995	4297,2475
**PSAT1**	4038,92	1047,68	5781,09	8296,1	977,31	14,32	4790,9475	495,815	4295,1325
**KANK2**	1643,12	3147,88	9698,57	10069,07	1734,68	1957,51	6139,66	1846,095	4293,565
**NR2F2**	48	4632,65	0	12695,73	97,96	9,34	4344,095	53,65	4290,445
**MGAT1**	8666,67	8986,25	9052,26	13479,42	6995,32	4517,23	10046,15	5756,275	4289,875
**PNPLA6**	3033,07	5855,52	7866,33	18523,46	6660,69	2403,31	8819,595	4532	4287,595
**TNIP1**	5738,57	15257,42	6901,79	9812,74	7259,43	3035,66	9427,63	5147,545	4280,085
**DDX39A**	15770,47	15158,72	13671,83	24446,43	16194,39	9798,46	17261,8625	12996,425	4265,4375
**SHFL**	913,47	1157,95	13092,32	6136,28	647,45	1475,92	5325,005	1061,685	4263,32
**BSDC1**	2633,19	4578,27	8669,6	10142,71	1950,2	2541,9	6505,9425	2246,05	4259,8925
**UBE2J2**	7030,04	6602,04	8500,2	9636,3	4215,94	3165,74	7942,145	3690,84	4251,305
**NADSYN1**	5542,19	7339,92	8211,38	7903,02	3114,56	2883,42	7249,1275	2998,99	4250,1375
**XRCC6**	50661,16	38772,57	33330,24	36629,74	39716,88	31483,27	39848,4275	35600,075	4248,3525
**BST2**	1078,33	19695,67	17421,03	14038,29	15945,08	1679,09	13058,33	8812,085	4246,245
**TRIM6**	6623,36	4775,73	1375,45	7434,67	0	1618,48	5052,3025	809,24	4243,0625
**MED25**	5688,08	9491,44	9597,28	9262,78	3819,62	4723	8509,895	4271,31	4238,585
**POM121**	12716,36	17056,23	15119,62	11545,81	7159,43	12591,98	14109,505	9875,705	4233,8
**WASH6P**	10582,54	5068,46	11037,28	10850,61	6875,27	3434,95	9384,7225	5155,11	4229,6125
**GATAD2A**	9443,27	13083,84	7141,49	12345,81	3234,16	9314,2	10503,6025	6274,18	4229,4225
**CPOX**	9119,3	7458,9	3944,39	10394,55	3549,51	3451,16	7729,285	3500,335	4228,95
**RASAL3**	5092,55	4787,95	9317,92	7811,49	1669,19	3384,01	6752,4775	2526,6	4225,8775

**ZYX**	17406,71	10389,08	17071,98	9772,44	13250,71	5618,62	13660,0525	9434,665	4225,3875
**TMEM164**	11729,74	9865,25	5133,45	8341	2952,27	6132,77	8767,36	4542,52	4224,84
**DCTPP1**	9893,96	8894,85	6712,82	10481,95	3753,29	5792,8	8995,895	4773,045	4222,85
**CRISP3**	37,11	11957,68	4806,06	68,96	0	11,31	4217,4525	5,655	4211,7975
**ANXA1**	8983,41	5201,75	17056,44	4548,57	2495,43	6977,47	8947,5425	4736,45	4211,0925
**SOCS3**	172,49	326,04	15012,02	1841,29	126,03	144,93	4337,96	135,48	4202,48
**TBL3**	9038,8	6088,2	4729,11	6866,73	4031,47	943,12	6680,71	2487,295	4193,415
**AGPAT1**	4927,18	8983,38	6696,47	9269,97	2832,02	3723,44	7469,25	3277,73	4191,52
**PDCD5**	6158,22	3461,97	13542,35	8211,9	5077,49	2234,26	7843,61	3655,875	4187,735
**NDUFB7**	5862,26	5613,82	9572,51	10668,25	4766	2719,28	7929,21	3742,64	4186,57
**PSMD1**	19511,81	7686,45	8901,02	12926,15	5733,5	10430,25	12256,3575	8081,875	4174,4825
**HPSE2**	6441,68	4890,92	2466,8	3005,77	7,9	46,6	4201,2925	27,25	4174,0425
**CLU**	1299,12	4218,95	6504,54	10112,67	127,94	2600,07	5533,82	1364,005	4169,815
**F11R**	1742,74	6814,99	7161,44	6739,16	790,72	2099,33	5614,5825	1445,025	4169,5575
**SLC2A3**	27994,53	31153,43	12362,26	16181,09	31527,11	3980,83	21922,8275	17753,97	4168,8575
**POLR2L**	6142,74	6335,19	10625,65	6884,7	5088,05	1569,49	7497,07	3328,77	4168,3
**IMP4**	8893,5	10010,53	7310,49	10440,1	6536,04	3458,75	9163,655	4997,395	4166,26
**TAPBP**	5413,81	8449,44	13706,03	7957,86	5270,38	4164,6	8881,785	4717,49	4164,295
**CMIP**	8139,32	14890,91	10092,72	13623,12	5754,72	9313,68	11686,5175	7534,2	4152,3175
**SLC52A2**	4651,83	10209,25	5546,11	5257,84	3352,22	1186,72	6416,2575	2269,47	4146,7875
**SNU13**	14945,55	11666,98	10029,73	12504,79	12119,53	4161,03	12286,7625	8140,28	4146,4825
**EZH2**	7923,8	7138,4	5617,14	4910,15	4391,54	112,41	6397,3725	2251,975	4145,3975
**SLC25A39**	11133,3	7541,35	12236,64	11923,93	7311,6	5817,61	10708,805	6564,605	4144,2
**KDM5C**	19540,91	23440,82	10779,21	10718,39	7496,96	16456,16	16119,8325	11976,56	4143,2725
**MRTO4**	9688,88	8027,76	9286,96	8776,6	6888,82	2719,3	8945,05	4804,06	4140,99
**HEXA**	5061,2	6638,05	19899,54	4643,89	6022,64	3823,85	9060,67	4923,245	4137,425
**ECSIT**	2796,08	3765,69	3935,97	19051,08	4550,73	1958,83	7387,205	3254,78	4132,425
**MT-ND6**	30716,07	46719,75	43633,94	27212,06	40807,18	25072,22	37070,455	32939,7	4130,755
**CD52**	534,78	2533,1	14048,45	2855,01	0	1748,49	4992,835	874,245	4118,59
**SIPA1**	11628,28	4524,9	10122,79	8014,07	7495,68	1415,24	8572,51	4455,46	4117,05
**PDLIM7**	5694,23	5935,61	10917,89	10951,91	6218,08	2309,88	8374,91	4263,98	4110,93
**LYAR**	6852,58	6270,22	14101,34	5597,66	5830,55	2360,76	8205,45	4095,655	4109,795
**ATP5PF**	9665,56	5349,34	16764,72	6766,45	7684,12	3372,72	9636,5175	5528,42	4108,0975

**DDX1**	18382,91	13409,63	12776,73	7027,95	10462,87	7129,52	12899,305	8796,195	4103,11
**CSNK1D**	11872,25	12132,91	15888,64	12589,55	8366,16	9679,29	13120,8375	9022,725	4098,1125
**RNF5**	4214,63	6964,13	9848,05	8532,4	4335,1	2275,4	7389,8025	3305,25	4084,5525
**U2AF1L5**	21150,89	3924,96	12442,33	8687,08	4383,99	10553,02	11551,315	7468,505	4082,81
**GLYR1**	9141,54	9516,5	5908,37	9155,74	5442,19	3279,27	8430,5375	4360,73	4069,8075
**GTF3C1**	13267,7	10633,29	13634,65	10632,65	11690,96	4254,15	12042,0725	7972,555	4069,5175
**MAGI1**	5479,88	6129,16	2319,23	2339,07	2,93	6,13	4066,835	4,53	4062,305
**PEPD**	4774,58	3451,73	11745,95	4446,8	1201,66	2890,9	6104,765	2046,28	4058,485
**SPATC1L**	4838,34	26,92	9835,26	6331,64	1671,17	729,54	5258,04	1200,355	4057,685
**RPA2**	10090,53	6952,55	10027,43	9729,15	5454,76	4835,76	9199,915	5145,26	4054,655
**RAB11B**	6303,64	7308,35	11276,04	12681,25	6236,67	4441,84	9392,32	5339,255	4053,065
**PLAGL1**	4598,49	4658,77	1587,33	9410,29	1097,64	928,85	5063,72	1013,245	4050,475
**GFUS**	2701,9	13662,34	4597,87	5864,41	4230,99	1088,85	6706,63	2659,92	4046,71
**ENPP3**	1331,26	40,7	6909,33	8300,25	3,94	206,63	4145,385	105,285	4040,1
**RUNX1**	9894,25	14348,41	12527,48	18613,31	2541,16	17081,02	13845,8625	9811,09	4034,7725
**YWHAQ**	25003,89	24334,65	16469,41	11578,34	15856,9	14779,46	19346,5725	15318,18	4028,3925
**SULT1A3**	10275,29	8165,39	1822,45	2669,77	1144,54	2282,34	5733,225	1713,44	4019,785
**KIF21B**	4497,51	2237,98	1596,18	18456,34	2019,02	3336,21	6697,0025	2677,615	4019,3875
**AP2S1**	8699,02	5784,95	16787,15	10786,88	9569,55	3441,86	10514,5	6505,705	4008,795
**CMPK1**	12657,25	6964,86	10067,52	14536,17	7753,57	6346,04	11056,45	7049,805	4006,645
**SEPHS2**	11056,34	18566,75	20744,83	33535,4	8742,46	25200,67	20975,83	16971,565	4004,265
**CCDC86**	7551,95	6177,8	5260,26	6809,75	3012,88	1892,84	6449,94	2452,86	3997,08
**LYPLA2**	5718,88	6313,6	13303,81	6291,5	3751,06	4105,29	7906,9475	3928,175	3978,7725
**SRSF5**	20682,21	29287,44	26472,84	13087,69	13832,36	22981,1	22382,545	18406,73	3975,815
**NAP1L4**	13053,83	10968,09	12755,3	13456,27	6367,45	10800,86	12558,3725	8584,155	3974,2175
**ITGA5**	8609,64	22270,32	4631	24771,75	12529,24	9667,6	15070,6775	11098,42	3972,2575
**OSBPL6**	5423,59	320,83	1736,17	8490,68	35,07	22,28	3992,8175	28,675	3964,1425
**SPTB**	2738,39	895,61	131,96	12110,33	15,28	12,33	3969,0725	13,805	3955,2675
**KCTD15**	2860,31	3673,81	4957,11	4504,06	87,73	0	3998,8225	43,865	3954,9575
**TNPO2**	7279,33	10774,79	7508,13	10561,4	3743,77	6409,01	9030,9125	5076,39	3954,5225
**MATR3**	6775,2	12024,93	3416,28	7961,31	3123,52	4060,15	7544,43	3591,835	3952,595
**PSTPIP2**	9157,21	5857,08	4236,44	5617,61	741,98	3794,09	6217,085	2268,035	3949,05
**RCC1**	13705,54	10688,74	9768,79	10946,95	7604,56	7056,49	11277,505	7330,525	3946,98

**FADS2**	44810,65	14489,85	55462,15	29669,07	13412,6	50915,43	36107,93	32164,015	3943,915
**GNG2**	3362,61	6557,46	2356,83	7623,62	523,02	1544,25	4975,13	1033,635	3941,495
**CD36**	3193,99	4,67	7188,72	20429,59	7497,82	28,52	7704,2425	3763,17	3941,0725
**MRPL12**	12694,28	11039,87	12730,66	9549,71	12347,66	2780,46	11503,63	7564,06	3939,57
**OTUD5**	9207,29	5809,73	7313,2	6880,77	2120,9	4606,5	7302,7475	3363,7	3939,0475
**CCNL2**	9225,63	8351,19	18616,8	12050,76	10713,19	5532,05	12061,095	8122,62	3938,475
**ACTR3**	17027,6	23203,65	14539,03	26960,77	16831,37	16165,81	20432,7625	16498,59	3934,1725
**SLC12A7**	4086,96	2352,3	13552,1	6144,33	174,62	5028,2	6533,9225	2601,41	3932,5125
**KDM5D**	8897,65	0	0	6931,4	55,02	3,32	3957,2625	29,17	3928,0925
**RBM4**	11312,45	9646,03	11307	6230,34	5842,89	5568,05	9623,955	5705,47	3918,485
**SRSF7**	27492,13	20902,12	18963,64	13395,01	21257,11	11283,68	20188,225	16270,395	3917,83
**PISD**	5155,8	6772,4	4900,93	7710,93	2353,96	2082,83	6135,015	2218,395	3916,62
**HNRNPL**	42267,92	33574,27	30480,12	30971,97	35186,57	25631,65	34323,57	30409,11	3914,46
**PSMB3**	13083,2	6405,72	13416,6	10331,1	8991,25	4820,14	10809,155	6905,695	3903,46
**TMEM14B**	4860,88	4605,43	11632,16	6462,54	3969,54	2004,98	6890,2525	2987,26	3902,9925
**PPP5C**	7951,21	6702,16	9020,48	9011,95	3901,71	4637,29	8171,45	4269,5	3901,95
**ID1**	943,35	1610,76	3930,48	17275,51	3491,6	588,45	5940,025	2040,025	3900
**HDGF**	23259,89	28642,14	33328,42	42188,92	35494,99	20431,83	31854,8425	27963,41	3891,4325
**ST3GAL4**	6168,85	2543,3	4177,82	13016,31	2752,91	2419,92	6476,57	2586,415	3890,155
**TSPAN4**	5828,3	6815,92	13777,08	5422,67	5017,72	3125,3	7960,9925	4071,51	3889,4825
**DCAF7**	12423,58	18146,28	17483,15	17118,51	14186,63	10626,88	16292,88	12406,755	3886,125
**PABPN1**	22935,23	18197,08	19910,17	15647,43	14136,46	16439,13	19172,4775	15287,795	3884,6825
**PITRM1**	6380,6	7732,37	10818,2	7452,28	5206,99	3216,39	8095,8625	4211,69	3884,1725
**MAVS**	11885,47	10804,15	10792,11	12830,66	6701,88	8688,48	11578,0975	7695,18	3882,9175
**USP11**	9949,06	6148,35	12010,33	8780,81	1282,68	9397,52	9222,1375	5340,1	3882,0375
**PGAM5**	6199,88	7715,58	6334,81	8531,96	2990,88	3637,59	7195,5575	3314,235	3881,3225
**PLEKHM2**	6067,04	5789,07	10860,64	11945,29	5036,3	4540,19	8665,51	4788,245	3877,265
**FGFR3**	4138,86	3784,65	3370,61	4320,12	51,2	5,22	3903,56	28,21	3875,35
**SAMD1**	8244,81	5941,24	8933,64	8514,21	3743,9	4323,59	7908,475	4033,745	3874,73
**RBPMS2**	9181,2	2075,25	8413,66	172,73	5,95	2169,7	4960,71	1087,825	3872,885
**ZPR1**	8578,55	5326,15	6034,6	11798,45	6131,88	1998,81	7934,4375	4065,345	3869,0925
**CBWD1**	4634,46	3698,85	3060,2	29059,94	9228,15	3264,56	10113,3625	6246,355	3867,0075
**IL27RA**	3706,37	2745,07	10014,27	4188,55	1078,58	1529,17	5163,565	1303,875	3859,69

**HMGN3**	7423,46	4983,44	11485,86	8197,33	3022,1	5323,38	8022,5225	4172,74	3849,7825
**COPS3**	11255,46	8623,78	7047,48	8452,83	6520,5	3470,85	8844,8875	4995,675	3849,2125
**TBC1D4**	6937,31	7294,6	8594,28	5289,05	1400,81	4959,13	7028,81	3179,97	3848,84
**TRIR**	10407,48	13537,37	16085,16	13803,37	12087,97	7132,09	13458,345	9610,03	3848,315
**MAPK8IP3**	4522,25	6808,93	11593,09	12026,28	5383,36	4427,07	8737,6375	4905,215	3832,4225
**PML**	4428,41	8677,89	10526,22	8205,77	4364,97	3892,13	7959,5725	4128,55	3831,0225
**NAPRT**	5273,32	502,52	7024,85	5964,49	314,47	1413,58	4691,295	864,025	3827,27
**MRPS15**	6952,2	5029,23	11587,35	6435,87	4925,88	2425,7	7501,1625	3675,79	3825,3725
**QARS1**	21145,98	20915,47	20916,2	13791,75	19052,24	11689,13	19192,35	15370,685	3821,665
**THAP4**	5862,51	3009,47	14352,66	5586,6	5043,79	1721,03	7202,81	3382,41	3820,4
**RRN3**	15004,9	5118,29	5179,09	7101,39	5642,28	2934,26	8100,9175	4288,27	3812,6475
**ECI2**	3271,97	4679,05	7777,05	5900,15	1947,2	1273,63	5407,055	1610,415	3796,64
**AGPAT4**	5030,28	1519,31	3331,28	9028,65	74,64	1789,14	4727,38	931,89	3795,49
**PQBP1**	10819,88	4659,23	7261,83	6891,31	4971,12	2262,73	7408,0625	3616,925	3791,1375
**PHF6**	6555,47	6031,63	3316,6	4968,9	2845,06	10,56	5218,15	1427,81	3790,34
**PITHD1**	7477,16	4889,1	9280,73	7468,64	3192,01	3785,55	7278,9075	3488,78	3790,1275
**CELF2**	10148,87	28230,52	8125,38	11665,68	9707,79	11803,68	14542,6125	10755,735	3786,8775
**UMPS**	7791,2	8701,2	4908,27	1641,8	2365,9	1583,13	5760,6175	1974,515	3786,1025
**XAB2**	6055,88	6511,53	7474,2	16945,5	6196,87	4725,56	9246,7775	5461,215	3785,5625
**NPEPPS**	11621,12	13140,57	9956	14209,36	5786,92	11108,09	12231,7625	8447,505	3784,2575
**FKBP8**	16151,14	25538,18	32272,53	20992,12	28777,64	11142,56	23738,4925	19960,1	3778,3925
**DIPK1B**	10698,85	1909,11	12325,19	2524,92	3157,3	3025,89	6864,5175	3091,595	3772,9225
**RPS6KB2**	7377,24	4726,76	7938,55	5970,45	2174,14	3293,74	6503,25	2733,94	3769,31
**ATP5PB**	17896,64	13938,96	22742,07	21531,3	20891,97	9632,31	19027,2425	15262,14	3765,1025
**LRPAP1**	7827,75	8355,97	8885,99	5022,57	4684,93	2846,51	7523,07	3765,72	3757,35
**DDX3Y**	6507,53	0	0	8981,1	239,91	0	3872,1575	119,955	3752,2025
**YOD1**	15284,11	3049,92	3003,49	8159,58	2391,4	4868,96	7374,275	3630,18	3744,095
**FTSJ3**	7750,05	6432,25	8274,4	7347,14	5553,05	1867,8	7450,96	3710,425	3740,535
**PPT2**	3546,38	4288,13	6468,34	5464,18	1072,92	1333,57	4941,7575	1203,245	3738,5125
**ERCC1**	6468,15	6915,69	15453,24	8759,02	7071,71	4251,66	9399,025	5661,685	3737,34
**TANC1**	3325,39	5733,04	7224,42	7598,36	401,98	4064,09	5970,3025	2233,035	3737,2675
**MDH2**	16634,6	18711,25	22392,31	14563,69	20371,38	8309,47	18075,4625	14340,425	3735,0375
**SCAP**	9351,47	11539,17	13070,97	6877,12	5615,07	7353,86	10209,6825	6484,465	3725,2175

**ROBO2**	6499,37	2759,85	4750,73	1465,72	270,59	21,45	3868,9175	146,02	3722,8975
**ATP6V0B**	10391,72	6564,72	10659,09	8342,12	6016,71	4518,85	8989,4125	5267,78	3721,6325
**LIF**	2575,22	862,19	2649,2	8877,73	14,28	25,32	3741,085	19,8	3721,285
**AAMP**	9317,97	9547,12	13387,86	9850,35	7742,18	5897,87	10525,825	6820,025	3705,8
**TOR3A**	10755,19	6754,98	3432,33	5378,46	2254,95	3497,77	6580,24	2876,36	3703,88
**TNKS1BP1**	3391,24	6848,99	5945,06	9579,68	3156,32	2320,24	6441,2425	2738,28	3702,9625
**LRBA**	9766,54	5658,55	10268,92	7630,86	3103,59	6179,99	8331,2175	4641,79	3689,4275
**MYBBP1A**	5589,37	12636,13	17757,51	9524,21	13809,91	1565,68	11376,805	7687,795	3689,01
**RPS6KA1**	8689,33	15382,9	12072,11	16742,2	14170,23	4898,6	13221,635	9534,415	3687,22
**TMED2**	12901,47	12972,91	11256,89	16515,96	8225,4	11226,47	13411,8075	9725,935	3685,8725
**TNS4**	0	4700,42	628,33	10388,5	0	499,22	3929,3125	249,61	3679,7025
**NMU**	2786,69	3311,92	1761,85	6855,15	0	5	3678,9025	2,5	3676,4025
**SLFN11**	6531,69	1,1	5198,88	6643,4	1815,39	24,81	4593,7675	920,1	3673,6675
**NAA10**	5836,67	4640,65	7504,37	9177,81	4193,13	2049,02	6789,875	3121,075	3668,8
**ATP1B3**	15867,72	5596,02	8445,65	6002,96	5281,35	5349,52	8978,0875	5315,435	3662,6525
**CD4**	10988,73	6242,09	16435,53	7010,16	12935,99	80,64	10169,1275	6508,315	3660,8125
**SLC5A6**	9123,85	6436,6	3637,59	3474,4	2170,95	1843,7	5668,11	2007,325	3660,785
**COLGALT1**	8500,54	12543,36	7517,81	23978,88	10319,44	8633,37	13135,1475	9476,405	3658,7425
**NOMO1**	13172,74	9413,79	24766,99	13536,98	9199,92	13935,91	15222,625	11567,915	3654,71
**VAT1**	5232,12	10628,81	21926,57	8848,09	11247,17	4766,52	11658,8975	8006,845	3652,0525
**BCAT2**	3539,6	5390,73	9652,58	4842,65	3030,48	1383,54	5856,39	2207,01	3649,38
**NECAP2**	4150,41	6025,07	9156,43	8812,55	4053,51	2724,53	7036,115	3389,02	3647,095
**ZNF598**	6633,76	8429,48	5395,48	8662,54	5110,29	2157,22	7280,315	3633,755	3646,56
**USP7**	12447,91	11788,09	4978,83	20279,57	7297,22	10162,82	12373,6	8730,02	3643,58
**RPL14**	99628,03	91620,73	129339,83	55753,32	141909,81	38983,2	94085,4775	90446,505	3638,9725
**TSR1**	6166,8	12141,49	9802,72	7710,88	8239,58	2397,01	8955,4725	5318,295	3637,1775
**MCUR1**	6778,8	3071,38	7678,46	5761,32	3016,79	1387,98	5822,49	2202,385	3620,105
**CPPED1**	7382,32	3748,62	5729,02	12498,9	4960,49	2479,33	7339,715	3719,91	3619,805
**SLC6A8**	25917,29	10861,12	6984,17	25709,66	26741	762,68	17368,06	13751,84	3616,22
**RIPOR3**	8395,05	74,3	5978,14	6654,15	12,46	3315,78	5275,41	1664,12	3611,29
**RBBP7**	24213,44	11637	10559,13	11194,84	6889,95	14692,94	14401,1025	10791,445	3609,6575
**SF3A3**	14225,26	10484,67	14805,14	14476,49	9960,15	9816,38	13497,89	9888,265	3609,625
**NSMF**	6628,43	6161,96	2738,93	6010,71	3153,76	399,23	5385,0075	1776,495	3608,5125

**PTPA**	7793,93	10330,13	12109,1	8245,38	6682,94	5362,46	9619,635	6022,7	3596,935
**RANBP1**	23137,72	16601,28	10862,55	12304,01	13319,64	10945,72	15726,39	12132,68	3593,71
**EIF3D**	27707,21	34145,99	21132,38	20077,77	32662,58	11685,32	25765,8375	22173,95	3591,8875
**CD33**	5048,26	7468,54	8800,16	7613,93	4783,19	2519,66	7232,7225	3651,425	3581,2975
**DAG1**	4973,59	7415,38	7376,81	4955,01	993,06	4206,31	6180,1975	2599,685	3580,5125
**KIFAP3**	16919,91	2917,45	4043,88	3828,72	1131,7	5569,84	6927,49	3350,77	3576,72
**CENPV**	7494,5	6612,32	5458,06	1306,65	1409,32	1873,85	5217,8825	1641,585	3576,2975
**SEPTIN8**	3367,41	4652,64	2664,9	18638,94	2553,81	4955,61	7330,9725	3754,71	3576,2625
**SELENBP1**	4738,95	267,85	9255,7	57,36	6,67	7,2	3579,965	6,935	3573,03
**CPSF6**	8989,61	13766,7	16328,04	9380,99	8518,89	8572,88	12116,335	8545,885	3570,45
**SIN3B**	4626,17	4809,15	7571,77	8703,21	2546,33	3168,32	6427,575	2857,325	3570,25
