# Supplementary material for: The Hematopoietic TALE-Code Shows Normal Activity of IRX1 in Myeloid Progenitors and Reveals Ectopic Expression of IRX3 and IRX5 in Acute Myeloid Leukemia
Source: Int J Mol Sci. 2022 Mar 16;23(6):3192. doi: 10.3390/ijms23063192 (PMC8952210; doi:10.3390/ijms23063192)
Supplement: Supplementary file 1 [file ijms-23-03192-s001.zip › Table S2_LL100_IRX1-IRX35.pdf]

gene	CMK	M-07e	MKPL-1	UT-7	OCI-AML3	MEGAL	IRX1	IRX35	IRX1-IRX35
ACTG1	223311,4	195232,3	203340,62	107478	582953,38	54097,98	182340,57	318525,68	-136185,11
CTSG	1,77	289,67	22,96	3,38	269981,04	27,96	79,445	135004,5	-134925,055
EEF2	187238,5	343697,2	117035,6	122441,9	481409,92	139799	192603,288	310604,435	-118001,148
TMSB4X	81648,53	71701,03	39238,17	39629,11	288980,69	35133,98	58054,21	162057,335	-104003,125
SRGN	21922,62	4772,76	20554,74	6478,63	160262,18	2639,78	13432,1875	81450,98	-68018,7925
NUP214	7062,72	9420,91	10963,74	6651,18	124576,92	13896,5	8524,6375	69236,71	-60712,0725
GAPDH	313436,1	299496,3	380097,24	211942,5	577973	144884,1	301243,035	361428,57	-60185,535
VIM	36691,45	67485,78	80245,62	53109,69	194598,9	37969,44	59383,135	116284,17	-56901,035
ZFP36L2	12593,51	21570,56	8441,19	28352,04	130472,95	14315,62	17739,325	72394,285	-54654,96
RPL37	44469,38	50700,52	151328,72	53360,76	233422,07	15194,34	74964,845	124308,205	-49343,36
IRF2BP2	24292,9	17892,97	15681,46	26842,12	120966,72	19142,75	21177,3625	70054,735	-48877,3725
NDRG1	335,13	11708,06	1752,64	4564,2	100229,19	726,46	4590,0075	50477,825	-45887,8175
RPL13	141573,6	159836	142575,15	132719,6	248888,84	127749,7	144176,09	188319,29	-44143,2
PABPC1	131408,7	128256,8	103102,1	101729,1	279922,48	33581,15	116124,17	156751,815	-40627,645
PLAC8	1589,34	967,13	2775,73	1291,84	81534,17	2166,96	1656,01	41850,565	-40194,555
IRF8	1,22	3,99	0	2,81	80286,41	0	2,005	40143,205	-40141,2
RPS24	59516,41	60919,59	65668,18	42124,09	152636,31	41029,89	57057,0675	96833,1	-39776,0325
RPS20	55872,51	60493,92	78893,66	41597,75	183201,38	14593,39	59214,46	98897,385	-39682,925
IRX3	0,8	12,28	5,81	0	56419,53	20277,02	4,7225	38348,275	-38343,5525
CDK6	19957,25	21927,26	23984,55	12394,89	8110,73	107564,1	19565,9875	57837,435	-38271,4475
RPL12	47984,23	79338,24	67530,07	46947,44	177074,61	18494,1	60449,995	97784,355	-37334,36
TOP2A	26909,86	31259,63	27427	30424,67	56040,13	75959,92	29005,29	66000,025	-36994,735
KIAA0930	3829,15	3215,76	1078,89	5103,72	76262,9	2400,55	3306,88	39331,725	-36024,845
MRC2	319,55	6807,71	2059,64	8720,13	74549,18	2564,94	4476,7575	38557,06	-34080,3025
SLC7A5	18506,09	21741,54	12652,51	13842,42	9553,78	89333,86	16685,64	49443,82	-32758,18
RPL41	71849,81	82056,07	113289,54	55009,55	198733,23	25538,26	80551,2425	112135,745	-31584,5025
PYGL	5639,92	4609,73	1637,41	1806,5	65059,66	4922,04	3423,39	34990,85	-31567,46
STK24	13573,32	11609,68	12937,54	12744,05	5426,76	83072,78	12716,1475	44249,77	-31533,6225
RPS3A	122926,4	121229,1	107001,2	165816,9	227120,07	92789,27	129243,395	159954,67	-30711,275
CAVIN1	297,89	7909,33	7331,86	13986,96	0	75769,77	7381,51	37884,885	-30503,375
SMC4	19849,53	15367,48	10203,65	12060,22	39995,45	48962,76	14370,22	44479,105	-30108,885
SRRM2	87505,5	74065,76	157825,54	71076,81	228385	25622,2	97618,4025	127003,6	-29385,1975

**RPL30**	54652,35	42856,85	60142,88	36233,45	139763,28	11202,53	48471,3825	75482,905	-27011,5225
**CXCL8**	1245,17	121,15	129,86	1160,9	54684,4	99,36	664,27	27391,88	-26727,61
**EIF3E**	34673,04	36031,58	27136,52	25538,54	104817,28	9767,6	30844,92	57292,44	-26447,52
**GRK2**	27961,68	17396,74	40813,73	22670,13	90465,11	16301,77	27210,57	53383,44	-26172,87
**IRS2**	892,78	1946,22	2231,52	2655,82	34262,53	21667,86	1931,585	27965,195	-26033,61
**BASP1**	2689,23	116,5	626,41	707,46	54033,51	3,46	1034,9	27018,485	-25983,585
**NUCKS1**	50491,6	24611,33	22007,52	32702,64	52939,85	63649,16	32453,2725	58294,505	-25841,2325
**CXCR4**	71,03	156,85	280,97	31,65	49716,75	1256,76	135,125	25486,755	-25351,63
**PGK1**	74320,96	77247,98	63506,1	47964,19	157408,45	22963,34	65759,8075	90185,895	-24426,0875
**RPS27**	36548,42	38872,77	55953,17	43470,88	122282,38	13898,11	43711,31	68090,245	-24378,935
**STT3B**	15504,33	15317,78	11120,85	9869,31	20126,27	54520,47	12953,0675	37323,37	-24370,3025
**LYST**	843,26	235,48	371,56	488,88	48666,23	872,37	484,795	24769,3	-24284,505
**SPTLC2**	4970,13	5991,07	4085,25	5178,37	42894,82	15571,76	5056,205	29233,29	-24177,085
**MEF2D**	2143,08	3971,73	1463,04	2793,32	50731,1	2275,28	2592,7925	26503,19	-23910,3975
**OGFRL1**	1557,76	1336,67	293,05	1176,73	41824,92	7842,45	1091,0525	24833,685	-23742,6325
**PPP1R27**	2,89	2,27	4,15	5,53	46473,29	9,87	3,71	23241,58	-23237,87
**LAMP5**	0	0	0	0	46162,91	0	0	23081,455	-23081,455
**RPL26**	58827,92	62493,33	65266,3	43590,54	144448,28	16720,07	57544,5225	80584,175	-23039,6525
**MDM4**	5308,81	3468,45	3469,01	4394,31	47022,37	7285,65	4160,145	27154,01	-22993,865
**CEBPA**	796,43	47,52	7002,18	13,5	49181,08	643,12	1964,9075	24912,1	-22947,1925
**NAP1L1**	28000,23	47461,79	14734,62	33252,9	68747,47	37398,96	30862,385	53073,215	-22210,83
**RPL9**	75516	63079,91	52755,79	50828,78	130941,69	33835,35	60545,12	82388,52	-21843,4
**IL17RA**	11,55	1975,18	0	0	42448,63	1250,21	496,6825	21849,42	-21352,7375
**RPL21**	55941,13	51045,11	39038,38	38861,01	120328,61	14052,96	46221,4075	67190,785	-20969,3775
**CITED2**	13467,51	24385,85	6840,08	28398,62	71108,43	7285,31	18273,015	39196,87	-20923,855
**GPX1**	33633,15	19988,47	45707,15	21871,28	90843,31	11370,17	30300,0125	51106,74	-20806,7275
**KCNAB2**	9017,97	4269,58	2579,55	16730,49	43239,28	13723,2	8149,3975	28481,24	-20331,8425
**RPL32**	59616,74	57498,93	80844,52	39241,24	132543,68	26024,33	59300,3575	79284,005	-19983,6475
**RPS15A**	51669,13	52582,65	50435,4	46072,07	108240,16	31511,02	50189,8125	69875,59	-19685,7775
**CD44**	6655,19	15861,09	3226,57	5068,92	32940,42	21673,79	7702,9425	27307,105	-19604,1625
**GRB2**	10429,57	12628,13	14395,2	13108,98	53165,25	10493,33	12640,47	31829,29	-19188,82
**BCL2**	898,9	5840,09	632,82	2615,24	18973,06	24203,55	2496,7625	21588,305	-19091,5425
**RPL23A**	76701,83	69265,15	68521,17	46644,31	130148,24	38587,93	65283,115	84368,085	-19084,97

**TIMP3**	80276	8739,87	9007,79	30307,9	101826,44	36,7	32082,89	50931,57	-18848,68
**HMGB2**	41637,4	33133,46	26735,87	33670,28	51237,15	53967,94	33794,2525	52602,545	-18808,2925
**AZU1**	36,72	349,51	228,17	135,11	37687,97	194,15	187,3775	18941,06	-18753,6825
**PPIA**	98637,9	75669,16	131765,41	94856,41	179263,43	58647,88	100232,22	118955,655	-18723,435
**CEBPD**	586,94	428,47	1240,65	950,92	38394,38	1,75	801,745	19198,065	-18396,32
**LIPA**	4095,96	2843,5	1898,97	1553,74	3066,51	38684,36	2598,0425	20875,435	-18277,3925
**RPL36A**	41291,23	35445,82	56189,34	30648,88	100894,61	17292,21	40893,8175	59093,41	-18199,5925
**LAT2**	3329,8	5770,17	4625,72	2161,32	34522,24	8717,92	3971,7525	21620,08	-17648,3275
**TGFBR1**	2199,79	3124,42	1102,69	2369,58	29797,75	8984,97	2199,12	19391,36	-17192,24
**MICAL1**	879,57	1543,96	915,81	2243,32	25787,52	11291,38	1395,665	18539,45	-17143,785
**SATB1**	473,29	2005,42	3834,34	230,95	24820,23	12676,88	1636	18748,555	-17112,555
**IST1**	8966,95	10367,69	8459,77	6515,98	7383,9	43530,81	8577,5975	25457,355	-16879,7575
**ANXA6**	680,8	2510,32	2380,17	463,8	26638,3	9951,87	1508,7725	18295,085	-16786,3125
**CORO1A**	17992,11	10441,93	1690,79	23629,27	47225,08	12219,17	13438,525	29722,125	-16283,6
**CBFB**	4884,96	8578,76	2715,05	3449,78	5234,18	37130,29	4907,1375	21182,235	-16275,0975
**BAZ1A**	10317,57	9754,59	8758,45	7005,48	43705,74	6718,23	8959,0225	25211,985	-16252,9625
**RPL39**	24670,8	24131,57	40392,27	23451,15	75910,71	12513,83	28161,4475	44212,27	-16050,8225
**NPM1**	138658,1	113371	69243,55	94685,06	188318,36	51531,96	103989,415	119925,16	-15935,745
**MT2A**	758,66	5837,41	3697,44	1556,22	37119,36	420,38	2962,4325	18769,87	-15807,4375
**ZFR**	6504,57	7333,37	8139,51	8368,13	36364,84	10225,42	7586,395	23295,13	-15708,735
**ACTR2**	34963,13	34779,12	21759,46	41324	53594,89	44103,77	33206,4275	48849,33	-15642,9025
**DAP**	6583,18	6625,23	16282,67	9597,01	36769,56	13733,3	9772,0225	25251,43	-15479,4075
**ITGB2**	8173,87	4601,1	6091,05	6208,37	42960,63	128,48	6268,5975	21544,555	-15275,9575
**MYBPH**	1,81	0	0	0	30441,75	1,79	0,4525	15221,77	-15221,3175
**LPCAT1**	3985,83	990,18	3430,92	996,41	27245,24	7306,91	2350,835	17276,075	-14925,24
**CACNA2D4**	1904,55	966,43	115,68	358,43	30920,99	581,78	836,2725	15751,385	-14915,1125
**CBX5**	11080,57	12976,71	7276,61	12994,14	12795,1	39110,2	11082,0075	25952,65	-14870,6425
**RPL35A**	34627,23	40731,51	50446,88	27516,7	92849,88	13521,75	38330,58	53185,815	-14855,235
**XPO1**	28375,06	20142,75	15181,32	24112,01	35954,16	37592,65	21952,785	36773,405	-14820,62
**PGAM1**	51055,56	45497,7	28630,44	25557,8	93648,57	11106,12	37685,375	52377,345	-14691,97
**CA2**	1348,16	2062,77	1046,15	7834,79	34573,61	1,62	3072,9675	17287,615	-14214,6475
**PLPPR3**	998,92	8172,29	1819,89	623,39	32931,47	816,9	2903,6225	16874,185	-13970,5625
**PRTN3**	0	402,61	12,12	5,66	28059,83	3,51	105,0975	14031,67	-13926,5725

**RAD21**	20875,2	16494,26	14479	17687,52	43561,49	19024,83	17383,995	31293,16	-13909,165
**FAM107B**	1301,76	1173,31	343,69	22,85	24331,1	4518,31	710,4025	14424,705	-13714,3025
**NAV3**	3,33	2,85	1,73	0	27385,38	4,62	1,9775	13695	-13693,0225
**RFLNB**	704,5	4830,09	2892,71	855,27	26707,95	5148,49	2320,6425	15928,22	-13607,5775
**PLEK**	9180,61	11080,61	849,45	1070,21	22340,34	15933,04	5545,22	19136,69	-13591,47
**RPL7**	146065,5	142989,9	129608,91	96253,1	249350,54	35054,69	128729,33	142202,615	-13473,285
**RXRA**	2177,81	328,56	1342,29	3803,16	24213,46	6400,45	1912,955	15306,955	-13394
**CAPG**	670,1	864,29	254,75	878,15	26770,73	1302,44	666,8225	14036,585	-13369,7625
**EDEM1**	4205,3	3921,53	2625,96	3987,51	30319,51	3703,62	3685,075	17011,565	-13326,49
**TOMM20**	33824,89	24900,25	19180,01	19660,97	56949,75	18337,77	24391,53	37643,76	-13252,23
**PREX1**	6328,55	14487,14	5562,2	1827,24	12232,48	28368,2	7051,2825	20300,34	-13249,0575
**HMGCS1**	15971,07	3640,23	4850,23	4241,17	7463,74	32656,18	7175,675	20059,96	-12884,285
**ELANE**	7,38	71,62	271,83	27,85	25890,84	0	94,67	12945,42	-12850,75
**RPL8**	108815	273390,5	167962,6	99293,64	323081,55	27235,8	162365,455	175158,675	-12793,22
**SERPINB8**	0	0	68,37	0,82	25567,2	15,79	17,2975	12791,495	-12774,1975
**FOSL2**	753,28	1827,93	7048,21	1443,99	29534,83	1215,61	2768,3525	15375,22	-12606,8675
**DHX38**	6331,7	7620,62	8394,83	5486,53	11191,94	27834,89	6958,42	19513,415	-12554,995
**PTPRN2**	23,24	15,85	136,01	0	24999,33	133,55	43,775	12566,44	-12522,665
**SMC2**	8553,39	6746,78	3061,45	7007,5	7352,24	30370,72	6342,28	18861,48	-12519,2
**RHOA**	37311,14	32406,65	34454,78	27353,69	57489,7	33240,78	32881,565	45365,24	-12483,675
**CFD**	928,4	1734,79	1121,8	387,7	26954,9	12,74	1043,1725	13483,82	-12440,6475
**MT1X**	429,85	928,11	2066,3	567,32	26727,48	63,3	997,895	13395,39	-12397,495
**CBX3**	21020,27	20211,49	13973,68	17613,27	29569,01	31432,06	18204,6775	30500,535	-12295,8575
**MT1E**	2,8	434,21	22,52	150,86	24831,84	37,59	152,5975	12434,715	-12282,1175
**MKI67**	29550,64	25730,19	11587,53	20208,55	11204,19	56737,97	21769,2275	33971,08	-12201,8525
**BAHCC1**	877,98	6888,34	807,74	37,7	21358,95	7339	2152,94	14348,975	-12196,035
**IPO5**	38912,96	22779,07	27340,85	17329,18	11933,86	65625,11	26590,515	38779,485	-12188,97
**SYK**	5709,6	10299,32	6167,14	5530,8	17639,59	20482,12	6926,715	19060,855	-12134,14
**SDCBP**	4612,45	5251,1	6275,05	8717,79	27959,3	8594,22	6214,0975	18276,76	-12062,6625
**MED13L**	4387,87	7795,29	5350,51	10272,53	12490,75	25470,06	6951,55	18980,405	-12028,855
**HOMER3**	185,69	289,18	296,78	26,91	24347,83	106,66	199,64	12227,245	-12027,605
**VCAN**	13,6	158,28	80,88	102,96	24219,97	8,4	88,93	12114,185	-12025,255
**ARHGDIB**	28122,03	32542,08	70265,01	25505,73	67965,4	34052,76	39108,7125	51009,08	-11900,3675

**EIF3C**	76740,94	58967,26	27613,35	52735,64	35497,02	96318,5	54014,2975	65907,76	-11893,4625
**CYBA**	16780,46	9797,72	14832,77	3271,65	24399,09	21664,7	11170,65	23031,895	-11861,245
**ALDOA**	183367,3	146769,7	133869,19	144394,4	252182,89	75629,92	152100,148	163906,405	-11806,2575
**SCD**	55949,94	40181,82	22935,02	12585,9	34241,75	55151,36	32913,17	44696,555	-11783,385
**TNRC18**	7177	17122,24	8788,42	13703,75	16391,13	30467,08	11697,8525	23429,105	-11731,2525
**RCSD1**	122,86	1256,01	2,06	367,39	15189,29	9028,44	437,08	12108,865	-11671,785
**HLA-E**	3020,43	22314,35	23690,8	8983,66	46655,36	5550,44	14502,31	26102,9	-11600,59
**ANP32B**	29916,99	28718	22629,22	29612,43	45007,68	33428,46	27719,16	39218,07	-11498,91
**MSN**	57276,99	85344,64	58135,83	78295,76	115917,5	46596,75	69763,305	81257,125	-11493,82
**MBNL1**	10489,46	11065,97	6609,22	11034,17	30579,6	11981,43	9799,705	21280,515	-11480,81
**MATR3**	28666,61	19672,43	18297,89	21172,05	27546,59	39300,69	21952,245	33423,64	-11471,395
**ATP6V1B2**	5381,23	5681,95	4417,1	5066,32	31096,81	2082,08	5136,65	16589,445	-11452,795
**CYP51A1**	6352,22	51,52	2042,7	3055,68	16888,2	11587,19	2875,53	14237,695	-11362,165
**H1-10**	10310,29	19877,05	6907,6	3935,35	22288,85	20830,75	10257,5725	21559,8	-11302,2275
**PTPRC**	17918,63	8503,61	10777,07	15779,1	34451,93	14495,48	13244,6025	24473,705	-11229,1025
**ADGRA2**	26,33	366,78	25,59	14,43	21881,92	708,68	108,2825	11295,3	-11187,0175
**HLA-B**	10514,55	20737,13	121235,5	2436,27	87329,55	12482,3	38730,8625	49905,925	-11175,0625
**TOP2B**	42675,71	39185,12	48796,72	19464,7	35234,38	62126,11	37530,5625	48680,245	-11149,6825
**ZDHHC3**	4694,28	4691,92	3544,68	2951,09	25538,62	4620,5	3970,4925	15079,56	-11109,0675
**AP1G1**	5977,64	5560,93	3797,65	4750,41	5114,06	27027,69	5021,6575	16070,875	-11049,2175
**TPP2**	17319,94	9254,31	16685,89	9124,98	13752,32	34437,3	13096,28	24094,81	-10998,53
**UBC**	56410,18	56237,63	42492,21	38433,57	74819,29	43953,11	48393,3975	59386,2	-10992,8025
**SEPTIN7**	11746,12	11465,2	7388,65	9569,78	17486,44	24558,74	10042,4375	21022,59	-10980,1525
**YWHAB**	22045,95	18374,89	20162,58	31165,5	27029,95	40728,1	22937,23	33879,025	-10941,795
**MT1G**	0	67,88	106,15	107,8	22002,45	0	70,4575	11001,225	-10930,7675
**RPL37A**	71675,85	72013,34	114762,82	60413,74	153449,68	27732,7	79716,4375	90591,19	-10874,7525
**ARL8B**	5004,58	4700,84	3240,38	3641,33	23243,08	6714,69	4146,7825	14978,885	-10832,1025
**INSIG1**	13705,6	5131,74	2508,72	2483,37	19647,97	13906,38	5957,3575	16777,175	-10819,8175
**MYL12A**	14080,12	10358,98	8195,61	12355,62	31727,37	12392,51	11247,5825	22059,94	-10812,3575
**DHFR**	9907,13	6979,62	3373,7	7773,22	8598,66	26968,6	7008,4175	17783,63	-10775,2125
**ESYT2**	5726,39	12705,55	5445,2	8416,49	18970,97	18701,19	8073,4075	18836,08	-10762,6725
**NACA**	57085,17	52778,12	70132,88	52620,87	114941,99	22769,32	58154,26	68855,655	-10701,395
**ARHGAP45**	7158,12	16337	11375,02	2969,74	32867,02	7384,02	9459,97	20125,52	-10665,55

**SLC3A2**	12530,61	12797,49	14251,09	10804,46	21128,1	25285,16	12595,9125	23206,63	-10610,7175
**ZMIZ1**	10463,09	14408,28	12644,69	10928,51	2443,12	42866,31	12111,1425	22654,715	-10543,5725
**DOCK2**	11269,21	91,73	1305,07	11229,99	16321,69	16501,62	5974	16411,655	-10437,655
**RPS13**	44587,91	36898,93	38919,84	34595,66	78556,75	19775,55	38750,585	49166,15	-10415,565
**ANKRD13D**	2850,39	2022,04	2260,52	1825,39	23471,4	1821,66	2239,585	12646,53	-10406,945
**PRKAR1A**	9083,36	15465,89	5202,19	10781,43	19223	21457,28	10133,2175	20340,14	-10206,9225
**RNASE2**	3,54	3,02	0	0	20344,87	2,62	1,64	10173,745	-10172,105
**IQGAP1**	9775,58	13972,35	12233,24	16774,36	16787,01	29904,51	13188,8825	23345,76	-10156,8775
**RPL38**	21627,01	18468,8	38006,34	21468,69	62499,69	7554,33	24892,71	35027,01	-10134,3
**CST3**	5861,63	9271,06	11988,1	6982,83	31140,02	6145,15	8525,905	18642,585	-10116,68
**HMGN2**	47067,92	32288,21	55527,86	25401,21	46720,85	53641,19	40071,3	50181,02	-10109,72
**XAGE1A**	8,8	0	0	0	20201,68	0	2,2	10100,84	-10098,64
**MYB**	17028,73	25002,29	19027,34	34680,03	36715,38	31319,67	23934,5975	34017,525	-10082,9275
**NNT**	7075,38	9352,73	8847,32	7064,03	23023,15	13155,89	8084,865	18089,52	-10004,655
**CALCOCO2**	1280,55	3705,33	2391,64	5291,27	12764,54	13443,71	3167,1975	13104,125	-9936,9275
**STAG2**	9019,01	9079,5	4093,57	8986,72	12796,59	22552,41	7794,7	17674,5	-9879,8
**PARVG**	2124,18	5435,21	3086,93	2234,5	19516,15	6564,59	3220,205	13040,37	-9820,165
**PTPN6**	11099,05	15464,91	10652,56	16404,45	39503,62	6893,51	13405,2425	23198,565	-9793,3225
**P2RY2**	842,99	55,25	500,82	127,75	20096,94	20,66	381,7025	10058,8	-9677,0975
**TMEM121B**	0	8,44	0	0	18881,39	454,09	2,11	9667,74	-9665,63
**EIF4A2**	22637,16	15020,61	16404	17200,24	40227,14	14722,99	17815,5025	27475,065	-9659,5625
**MYLIP**	1319,48	1598,86	2686,91	2637,51	22118,32	1214,55	2060,69	11666,435	-9605,745
**SLC38A1**	108,39	11528,93	1837,92	99,71	76	25915,72	3393,7375	12995,86	-9602,1225
**WDFY1**	1677,13	2507,21	1298,34	1545,09	17721,19	4988,34	1756,9425	11354,765	-9597,8225
**PALM2AKAP2**	8493,87	457,74	2654,56	17677,43	10149,21	23650,32	7320,9	16899,765	-9578,865
**MYBL2**	11997,47	19157,17	9826,95	16520,81	8267,42	39628,52	14375,6	23947,97	-9572,37
**CHD9**	4460,68	5516,1	3608,94	3117,82	5611,78	21720,49	4175,885	13666,135	-9490,25
**DMXL2**	1008,94	885,69	350,94	6,63	15019,68	4976,67	563,05	9998,175	-9435,125
**TM9SF2**	14587,75	8302,76	20545,44	11641,07	6485,54	39804,54	13769,255	23145,04	-9375,785
**TRIP6**	55,63	73,41	44,65	148,18	18771,02	86,3	80,4675	9428,66	-9348,1925
**NEK7**	3975,12	1860,48	1304,7	1948,89	17439,44	5793,58	2272,2975	11616,51	-9344,2125
**MARCHF6**	6635,33	10754,96	14527,9	7694	24916,79	13459,23	9903,0475	19188,01	-9284,9625
**HK2**	11276,83	9719,84	4153,27	10980,68	31158,53	5476,19	9032,655	18317,36	-9284,705

**PTK2B**	4496,78	9845,87	2759,95	10071,8	30403,1	1596,74	6793,6	15999,92	-9206,32
**TLR2**	14,46	10,79	5,34	7,21	18309,24	0	9,45	9154,62	-9145,17
**OAZ1**	54194	43598,4	51104,91	29941,34	77725,43	29769,09	44709,6625	53747,26	-9037,5975
**PTEN**	5632,7	7470,54	5389,27	3401,1	19974,39	9029,71	5473,4025	14502,05	-9028,6475
**CES1**	16,91	89,69	29,67	268,52	18256,63	0,91	101,1975	9128,77	-9027,5725
**UBAC2**	6279,81	5080,67	12432,47	5622,58	9165,85	23478,45	7353,8825	16322,15	-8968,2675
**CGGBP1**	3974,19	8878,84	3436,51	6478,13	8087,51	21194,84	5691,9175	14641,175	-8949,2575
**XPO6**	9272,19	12068,9	8830,6	8825,67	16088,22	21277,17	9749,34	18682,695	-8933,355
**SNTA1**	211,26	6276,92	178,15	177,81	293,58	20952,21	1711,035	10622,895	-8911,86
**RBM39**	20879,38	20527,71	16803,94	32571,15	24212,75	38990,59	22695,545	31601,67	-8906,125
**P4HA1**	10043,98	6098,37	4780,17	5387,35	28340,55	2591,52	6577,4675	15466,035	-8888,5675
**FANCI**	9721,39	10079,38	6116,43	7636,4	4079,18	30422,61	8388,4	17250,895	-8862,495
**ARHGAP30**	4197,63	7105	4972,62	8860,29	18675,41	11545,39	6283,885	15110,4	-8826,515
**ZC3H11A**	17308,26	11311,22	10929,9	15163,74	23115,19	21857,09	13678,28	22486,14	-8807,86
**MNDA**	3,33	0	36,35	0	17623,57	0	9,92	8811,785	-8801,865
**ATP11A**	3921,7	8339,8	4009,14	5360,8	4751,13	23654,88	5407,86	14203,005	-8795,145
**FNBP1**	2493,75	3982,13	7685,47	2469,49	11898,83	14003,3	4157,71	12951,065	-8793,355
**DOCK10**	88,74	7,36	613,81	3,38	12374,52	5540,49	178,3225	8957,505	-8779,1825
**HNRNPK**	37626,71	54039,9	38194,7	46268,82	51422,17	54088,71	44032,5325	52755,44	-8722,9075
**SFMBT2**	271,2	3387	20,19	4,12	15104,21	4153,79	920,6275	9629	-8708,3725
**FUS**	25601,63	35227,73	29755,75	38609,93	40905,54	40883,65	32298,76	40894,595	-8595,835
**CD74**	3959,32	25,61	28,77	9350,94	8799,41	14978,26	3341,16	11888,835	-8547,675
**SUB1**	14822,81	12178,85	28253,76	16706,73	42727,68	10329,43	17990,5375	26528,555	-8538,0175
**LTA4H**	5840,67	6437,05	9060,15	4028,89	25889,14	3812,96	6341,69	14851,05	-8509,36
**ANKRD10**	6632,85	4497,66	14977,21	3999,17	3808,73	28214,13	7526,7225	16011,43	-8484,7075
**SREBF2**	19241,56	21851,2	12623,78	15941,51	20448,29	31324,59	17414,5125	25886,44	-8471,9275
**PANK3**	7857,34	4978,33	2735,25	5625,2	9539,93	17900,46	5299,03	13720,195	-8421,165
**PIK3AP1**	2521,11	5206,77	283,42	6852,02	13451,99	10722,59	3715,83	12087,29	-8371,46
**HSPA1A**	235,24	0	1303,5	14253,21	19900,43	4738,06	3947,9875	12319,245	-8371,2575
**TMEM123**	20664,49	16568,68	11815,85	15743,57	20183,35	28918,74	16198,1475	24551,045	-8352,8975
**ANKRD11**	17160,91	17348,89	13852,87	11433,57	10987,95	35516,02	14949,06	23251,985	-8302,925
**MMP14**	472,31	9148,5	1861,35	427,85	22365,52	158,22	2977,5025	11261,87	-8284,3675
**CCR1**	22,1	127,48	3,67	0	16596,94	30,58	38,3125	8313,76	-8275,4475

ATXN1L	2160,35	3658,58	1688,79	2236,07	1701,88	19717,51	2435,9475	10709,695	-8273,7475
LGALS1	3127,76	26500,39	20226,38	28133,29	40347,49	15144,1	19496,955	27745,795	-8248,84
TMPO	19833,73	20000,32	6895,49	15278,2	11324,7	36085,38	15501,935	23705,04	-8203,105
HOXA10	1504,76	16,68	2365,47	1,69	13228,53	5088,52	972,15	9158,525	-8186,375
TMC8	7522,57	4909,88	12560,91	7570,88	16839,56	15777,91	8141,06	16308,735	-8167,675
AK2	21513,22	22197,02	25700,67	35382,43	52829,38	15870,71	26198,335	34350,045	-8151,71
MS4A3	10998,15	609,57	8504,38	585,53	26545,5	31,77	5174,4075	13288,635	-8114,2275
SF3B3	20948,91	26654,3	15364,02	21260,4	15717,09	42569,3	21056,9075	29143,195	-8086,2875
MTPN	14794,13	12778,32	8577,56	14268,09	20967,59	20363,5	12604,525	20665,545	-8061,02
FTL	99880,04	102584,4	161066,76	155119,2	253836,67	21519,31	129662,618	137677,99	-8015,3725
SEC23B	5634,65	3863,42	5675,73	6949,43	21152,33	5920,48	5530,8075	13536,405	-8005,5975
CPNE1	10299,46	6058,92	10128,54	13975,73	12910,8	23143,95	10115,6625	18027,375	-7911,7125
SLC44A1	4899,85	2047,77	2437,49	4725,92	1246,58	21626,05	3527,7575	11436,315	-7908,5575
NCF1	658,51	132,38	91,71	209,03	16298	57,82	272,9075	8177,91	-7905,0025
FDPS	14503,07	12259,28	10381,3	9981,19	23539,87	15772,57	11781,21	19656,22	-7875,01
ABCC1	10373,96	14693,69	10498,12	10409,96	2652,69	36059,58	11493,9325	19356,135	-7862,2025
ANP32E	13167,33	12290,08	9688,53	12947,91	26096,73	13674,08	12023,4625	19885,405	-7861,9425
MXI1	665,26	1485,75	698,09	2481,24	16606,49	1777,2	1332,585	9191,845	-7859,26
TUBGCP3	10386,45	3759,58	8961,11	4695,3	6029,04	23544,19	6950,61	14786,615	-7836,005
COL2A1	206,39	106,16	3261,29	2,45	0	17420,61	894,0725	8710,305	-7816,2325
EDEM3	5540,4	2463,32	3778,44	3752,64	17565,21	5828,79	3883,7	11697	-7813,3
RPL15	96443,38	105685,1	80463,74	61347,09	131967,28	55560,37	85984,8225	93763,825	-7779,0025
RNASET2	732,45	5201,86	4403,07	10876,11	24944,08	1189	5303,3725	13066,54	-7763,1675
OTULINL	154,69	107,12	395,8	185,68	13188,67	2728,91	210,8225	7958,79	-7747,9675
BTF3	40961,94	36110,97	32718,7	36498,19	66978,05	21627,42	36572,45	44302,735	-7730,285
GNA13	5077,54	4624,24	2340,88	5496,93	12701,91	11527,67	4384,8975	12114,79	-7729,8925
SLC1A4	1501,84	1448,18	1797,19	2685,56	10460,34	8657,36	1858,1925	9558,85	-7700,6575
TUBA1A	890,52	2110,64	14616,19	4800,48	3987,1	22529,16	5604,4575	13258,13	-7653,6725
NREP	9648	11619,11	5463,19	2527,98	8212,63	21664,7	7314,57	14938,665	-7624,095
DHRS9	7965,44	0	5416,97	45,69	21939,09	2,51	3357,025	10970,8	-7613,775
B3GNT2	8900,25	7128,11	3639,38	4063,6	14768,71	12317,88	5932,835	13543,295	-7610,46
PIK3CG	1161,76	1968,54	1571,38	1407,32	15444	2817,23	1527,25	9130,615	-7603,365
NIPBL	7799,38	5964,34	11496,84	7064,32	21597,32	9653,61	8081,22	15625,465	-7544,245

**ETV6**	7617,86	5691,4	14572,63	5477,99	12822,71	18933,7	8339,97	15878,205	-7538,235
**DDX17**	30314,62	34588,47	20065,91	31207,66	26231,88	46834,02	29044,165	36532,95	-7488,785
**HDHD5**	0	4141,24	13,36	0	15455,19	1564	1038,65	8509,595	-7470,945
**CDC42EP3**	3125,72	2005,54	1393	632,06	15674,38	2822,37	1789,08	9248,375	-7459,295
**PRR11**	5569,73	5883,77	5168,4	5565,57	16377,09	9616,65	5546,8675	12996,87	-7450,0025
**MT-ATP8**	21630,04	31802,53	24370,35	21544,81	17226,57	47330,77	24836,9325	32278,67	-7441,7375
**RNH1**	13786,07	12840,58	20106,22	20248,53	45641,94	2725,03	16745,35	24183,485	-7438,135
**HMGCR**	10607,34	5545,98	2949,06	4667,59	8312,63	18355,91	5942,4925	13334,27	-7391,7775
**HIPK3**	2129,29	1355,68	1342,65	1953,89	14191,15	3968,17	1695,3775	9079,66	-7384,2825
**PCM1**	13224,48	13149,6	15153,22	12938,34	32805,23	9107,38	13616,41	20956,305	-7339,895
**CCNI**	19789,12	19740,11	16028,71	23785,05	31400,54	22851,52	19835,7475	27126,03	-7290,2825
**TASOR**	2644,25	3957,18	2070,68	2999,5	5317,27	15062,65	2917,9025	10189,96	-7272,0575
**ACSL1**	1869,56	789,69	573,05	5215,46	17920,8	841,93	2111,94	9381,365	-7269,425
**LRRFIP1**	4162,72	7918,12	2894,25	3984,53	11642,67	12368,48	4739,905	12005,575	-7265,67
**CDK1**	8968,4	5669,33	2007,2	4600,42	8790,13	16347,08	5311,3375	12568,605	-7257,2675
**LYZ**	4346,41	2750,47	11937,71	4711,31	22728,69	3643,99	5936,475	13186,34	-7249,865
**C12orf75**	80,52	133,82	345,57	913,91	9992,63	5233,71	368,455	7613,17	-7244,715
**TRPM2**	31,28	246,07	17,29	1,4	14590,34	17,54	74,01	7303,94	-7229,93
**WNK1**	7610,06	14068,51	5815,89	9201,81	6724,58	26072,45	9174,0675	16398,515	-7224,4475
**CKAP4**	25,99	181,86	251,09	8,82	12885,01	1746,91	116,94	7315,96	-7199,02
**CD37**	1256,75	1194,02	329,39	2792,94	12978,12	4199,99	1393,275	8589,055	-7195,78
**HOXA9**	30,69	0	508,9	1,95	10089,38	4557,01	135,385	7323,195	-7187,81
**QSOX1**	6809,81	4469,07	15477,2	9681,42	27587,77	4976,48	9109,375	16282,125	-7172,75
**HILPDA**	1235,12	1422,34	887,27	848,68	15700,01	789,91	1098,3525	8244,96	-7146,6075
**NUP205**	16632,97	11843,17	10327,36	9565,52	26176,6	12267,09	12092,255	19221,845	-7129,59
**SPIN1**	2292,94	5538,42	2109,38	2496,02	4260,12	16138,39	3109,19	10199,255	-7090,065
**TBC1D5**	4591,39	4369,98	1689,36	2781,84	7627,03	13267,55	3358,1425	10447,29	-7089,1475
**BMI1**	2628,88	2443,74	2303,21	4324,79	1327,18	18695,63	2925,155	10011,405	-7086,25
**KMT2C**	5135,57	4216,69	3421,8	4507,82	12735,74	10074,19	4320,47	11404,965	-7084,495
**RAD23B**	13438,09	13534,08	6644,88	17920,39	16337,87	23599,6	12884,36	19968,735	-7084,375
**HIP1**	460,15	138,29	775,13	1252,17	13413,01	2011,38	656,435	7712,195	-7055,76
**ATG16L2**	3769,83	2567,75	2698,03	6680,49	16309,46	5623,8	3929,025	10966,63	-7037,605
**ANKRD12**	4784,44	3672,14	3747,43	5073,4	16906,49	5804,3	4319,3525	11355,395	-7036,0425

**EIF3H**	16943,89	18736,83	16996,14	14838,77	41308,48	6499,65	16878,9075	23904,065	-7025,1575
**NCOA4**	19532,61	21221,05	8516,06	20297,93	31183,66	17630,13	17391,9125	24406,895	-7014,9825
**TGOLN2**	11598,73	13739,7	11284,53	14504,05	22683,64	16872,8	12781,7525	19778,22	-6996,4675
**ATXN2L**	18653,49	16902,2	17487,01	19486,8	19738,25	30514,93	18132,375	25126,59	-6994,215
**TPX2**	16416,33	15766,13	11208,9	21361,7	11884,41	34428,57	16188,265	23156,49	-6968,225
**RPS15**	51080	55584,96	34887,45	20675,76	72309,94	22734,9	40557,0425	47522,42	-6965,3775
**RUNX2**	4,57	90,53	85,94	62,17	13441,12	529,93	60,8025	6985,525	-6924,7225
**NCAPG2**	6270,24	5046,02	3351,57	4643,35	11900,8	11592,79	4827,795	11746,795	-6919
**CHSY1**	2884,71	4424,37	10239,41	3673,36	15033,37	9300,37	5305,4625	12166,87	-6861,4075
**CNTRL**	5281,78	6922,98	5309,6	4861,08	4747,82	20147,51	5593,86	12447,665	-6853,805
**SMC3**	13735,56	9289,79	4694,45	7319,27	12000,78	19167,89	8759,7675	15584,335	-6824,5675
**MYCN**	294,28	2260,78	14,13	133,41	1,34	14998,98	675,65	7500,16	-6824,51
**ATP8B4**	159,56	216,42	190,53	184,81	13611,74	318,86	187,83	6965,3	-6777,47
**RBL2**	3588,99	4724,94	1897,13	2770,72	6463,86	13580,91	3245,445	10022,385	-6776,94
**LRRC75A**	6987,41	8409,29	13092,2	4259,36	26031,71	3848,52	8187,065	14940,115	-6753,05
**HOXA13**	1,77	0	4,59	0	11429,88	1993,16	1,59	6711,52	-6709,93
**MAZ**	29475,75	43800,71	17155,4	33512,12	35312,33	40075,32	30985,995	37693,825	-6707,83
**UHRF2**	2078,07	3955,02	1977,57	3316,96	13164,75	5884,91	2831,905	9524,83	-6692,925
**DYNC1LI2**	2602,37	3115	3450,33	3336,22	4356,91	15265,69	3125,98	9811,3	-6685,32
**CAPRIN1**	19675,76	13697,51	13036,68	23048,35	22127,75	25971,99	17364,575	24049,87	-6685,295
**HNRNPDL**	41703,84	31083,68	28554,21	21021,12	48833,35	25712,5	30590,7125	37272,925	-6682,2125
**ZNF395**	4129,96	8164,32	3125,17	5291,83	19825,75	3889,4	5177,82	11857,575	-6679,755
**XYLT1**	1141,17	2804,66	483,04	760,74	2046,05	13895,32	1297,4025	7970,685	-6673,2825
**LBR**	16157,65	9612,01	5809,87	10719,15	13166,68	21309,58	10574,67	17238,13	-6663,46
**CHD2**	7025,37	8751,49	8283,02	9119,84	15425,84	14468,11	8294,93	14946,975	-6652,045
**CD93**	154,69	586,13	242,44	25,88	11115,52	2692,21	252,285	6903,865	-6651,58
**ATP13A3**	7732,32	9381,01	2902,93	9723,43	8107,79	20051,12	7434,9225	14079,455	-6644,5325
**IRX5**	162,38	223,34	61,02	181,44	9286,03	4235,04	157,045	6760,535	-6603,49
**SUMO2**	19241,37	15212,33	17421,75	15248,85	29839,58	16925,85	16781,075	23382,715	-6601,64
**MEF2A**	2395,69	2365,31	1499,47	1686,38	1597,83	15481,34	1986,7125	8539,585	-6552,8725
**CCL5**	77,12	185,04	179,75	31,14	13300,67	21,4	118,2625	6661,035	-6542,7725
**CHST11**	3248,79	20002,03	2988,38	5419,6	11661,2	17203,74	7914,7	14432,47	-6517,77
**CSTA**	1,78	4,55	22,16	10,18	13051,79	1,52	9,6675	6526,655	-6516,9875

**ENO2**	2915,35	2376,52	2225,44	2636,82	9746,26	8363,54	2538,5325	9054,9	-6516,3675
**SET**	51823,5	39469,69	25201,1	39508,73	39886,64	51131,85	39000,755	45509,245	-6508,49
**RBM33**	5129,81	6093,89	3428,03	4896,78	14482,71	8170,89	4887,1275	11326,8	-6439,6725
**GNAS**	57850,34	48296,1	72790,63	47652,6	39923,66	86180,74	56647,4175	63052,2	-6404,7825
**CHD6**	1398,54	4419,89	2640,44	4796,76	5719,85	13713,26	3313,9075	9716,555	-6402,6475
**BMP2**	7,96	6424,02	21,12	818,13	16310,59	51,55	1817,8075	8181,07	-6363,2625
**CTNNAL1**	3007,56	1563,66	1866,73	2234,14	10099,15	6954,95	2168,0225	8527,05	-6359,0275
**IGF2BP2**	11,2	3176,97	1605,47	0	8886,74	6223,76	1198,41	7555,25	-6356,84
**C1orf43**	15579,58	13375,51	15809,41	14411,04	32638,09	9631,43	14793,885	21134,76	-6340,875
**IGF2R**	360,58	8977,76	2352,79	5394,76	11508,06	9687,64	4271,4725	10597,85	-6326,3775
**ASPM**	11683,48	6203,53	4109	7485,81	9406,41	17942,59	7370,455	13674,5	-6304,045
**RNF166**	3029,24	3219,43	2482,4	2463,04	8305,9	9842,64	2798,5275	9074,27	-6275,7425
**PPP1CC**	18356,48	15729,76	9052,14	18606,08	25713,11	17680,54	15436,115	21696,825	-6260,71
**CMTM6**	6910,65	4117,23	3179,22	2214,69	4986,4	15739,06	4105,4475	10362,73	-6257,2825
**UHMK1**	8774,21	4907,55	3571,28	10251,19	14733,77	11485,41	6876,0575	13109,59	-6233,5325
**APEX1**	18609,21	23278,5	12265,42	11794,32	31519,22	13846,42	16486,8625	22682,82	-6195,9575
**SMYD3**	2455,73	1751,18	3223,24	1983,52	14644,06	2444,21	2353,4175	8544,135	-6190,7175
**TNFRSF1B**	383,12	562,3	203,09	555,53	13096,08	119,92	426,01	6608	-6181,99
**KIF5B**	13135,37	10932	9821,42	10188,91	14514,76	19826,74	11019,425	17170,75	-6151,325
**CEP20**	3905,63	2585,1	2617,81	4157,22	6644,02	12272,87	3316,44	9458,445	-6142,005
**PPDPF**	2400,11	3665,56	6726,07	2230,91	12958,95	6809,23	3755,6625	9884,09	-6128,4275
**RNASE1**	2187,48	1393,28	2919,87	290,9	15616,27	1,92	1697,8825	7809,095	-6111,2125
**NAE1**	7151,45	5180,29	3385,99	5267,42	4824,35	17889,19	5246,2875	11356,77	-6110,4825
**BTG1**	1189,78	647,98	1831,13	1605,87	12554,53	2298,12	1318,69	7426,325	-6107,635
**LDHB**	91233,81	75596,83	100098,76	63406,27	119800,12	57551,19	82583,9175	88675,655	-6091,7375
**XPOT**	13877,31	11088,92	7056,35	19295,41	14109,03	23725,84	12829,4975	18917,435	-6087,9375
**ANKH**	1536,13	2057,05	1421,59	208,64	8902,02	5842,95	1305,8525	7372,485	-6066,6325
**PHF10**	9522,64	5502,61	3488,33	7934,73	16061,09	9293,89	6612,0775	12677,49	-6065,4125
**ITM2C**	1306,84	36,81	1221,83	51,89	4815,48	8623,6	654,3425	6719,54	-6065,1975
**LRRC58**	8635,22	5005,85	2864,24	3777,8	10716,24	11514,19	5070,7775	11115,215	-6044,4375
**AFF1**	2837,82	4385,76	2243,43	6697,08	10812,17	9265,64	4041,0225	10038,905	-5997,8825
**SKA2**	2755,03	4393,93	5689,33	2787,39	12589,44	7177,3	3906,42	9883,37	-5976,95
**NUSAP1**	8796,29	6828,06	4050,24	6185,66	7357,98	17524,69	6465,0625	12441,335	-5976,2725

**CYBB**	891,01	574,81	326,92	42,76	12818,3	27,96	458,875	6423,13	-5964,255
**TPP1**	7902,18	10016,69	6009	5345	20186,88	6371,15	7318,2175	13279,015	-5960,7975
**OPTN**	12,35	1952,1	31,49	1350,65	11400,37	2189,2	836,6475	6794,785	-5958,1375
**SEPTIN6**	6139,64	12461,79	15006,84	5089,52	1528,61	29724,78	9674,4475	15626,695	-5952,2475
**EIF4G3**	4307,19	4784,37	10138,49	5654,76	16314,21	8018,93	6221,2025	12166,57	-5945,3675
**KTN1**	15419,4	14939,15	11224,22	12113,47	19610,96	19020,05	13424,06	19315,505	-5891,445
**BPTF**	8911,33	6839,93	6945,22	7477,19	12160,7	14675,57	7543,4175	13418,135	-5874,7175
**USP1**	8393,75	7454,39	4053,87	5774,47	6566,36	18010,23	6419,12	12288,295	-5869,175
**AZIN1**	7607,44	7479,03	4281,61	7546,4	17148,97	8023,95	6728,62	12586,46	-5857,84
**MSI2**	2216,32	4539,65	5549,89	4440,73	474,23	19609,44	4186,6475	10041,835	-5855,1875
**MAPRE1**	17229,78	12233,25	11431,24	21075,54	9973,7	32718,05	15492,4525	21345,875	-5853,4225
**ZEB2**	9505,54	7208,7	11476,93	7289,34	24263,18	5175,69	8870,1275	14719,435	-5849,3075
**POMK**	3902,38	1859,67	4104,69	2254,68	16005,76	1711,58	3030,355	8858,67	-5828,315
**GNAQ**	3840,6	10639,66	5744,49	8653,49	3836,1	22184,32	7219,56	13010,21	-5790,65
**FAM43A**	494,12	12,07	439,87	9	11784,89	214,96	238,765	5999,925	-5761,16
**ARHGEF7**	7266,17	4481,75	9621,75	4925,57	4257,56	20408,71	6573,81	12333,135	-5759,325
**IRF5**	826,54	2958,63	1090,79	396,09	10826,34	3328,17	1318,0125	7077,255	-5759,2425
**VIRMA**	8167,38	5376,89	8581,21	5834,35	23161,85	2333,91	6989,9575	12747,88	-5757,9225
**KIF20B**	5358,58	4944,9	2989,44	4501,99	9820,98	10550,96	4448,7275	10185,97	-5737,2425
**UNCX**	0	1,51	0	0	11378,39	88,25	0,3775	5733,32	-5732,9425
**LILRB4**	0	0	0	0	11443,52	1,24	0	5722,38	-5722,38
**LMO4**	2482,3	965,66	7158,62	1435,31	683,5	16776,42	3010,4725	8729,96	-5719,4875
**JUN**	75,42	105,08	721,91	601,75	11278,96	871,67	376,04	6075,315	-5699,275
**RAB37**	2322,69	511,99	15833,04	1747,81	3239,41	18256,08	5103,8825	10747,745	-5643,8625
**EMB**	3398,28	238,86	889,18	733,38	7148,82	6735,67	1314,925	6942,245	-5627,32
**FAM111A**	3302,62	3960,66	2232,65	4578,42	1103,67	17184,42	3518,5875	9144,045	-5625,4575
**DNMT3B**	3823,53	4161,56	5435,12	2341,63	742,47	18389,19	3940,46	9565,83	-5625,37
**FAM98B**	3670,23	2419,85	3193,32	2709,96	2332,71	14860,03	2998,34	8596,37	-5598,03
**SMC1A**	23599,7	16292,94	4586,73	11024,95	5032,06	33913,78	13876,08	19472,92	-5596,84
**RAB27B**	403,7	2751,47	36,76	315,32	363,95	12582,84	876,8125	6473,395	-5596,5825
**STMN3**	2572,85	4969,04	8985,67	824,85	533,09	19313,67	4338,1025	9923,38	-5585,2775
**DPYSL2**	2863,22	2028,94	264,35	207,18	9907,17	3928,63	1340,9225	6917,9	-5576,9775
**FCER1G**	1086,99	1914,9	2563,57	998,35	13161,11	1250,3	1640,9525	7205,705	-5564,7525

**RGS18**	5574,13	211,97	2447,32	1008,31	10027	5713,84	2310,4325	7870,42	-5559,9875
**PDZD8**	29750,81	24794,64	13567,25	18581,76	3462,48	50982,42	21673,615	27222,45	-5548,835
**IDI1**	9212,66	2715,22	5039,57	4687,69	9303,66	12614,16	5413,785	10958,91	-5545,125
**TMEM170B**	1832,41	1090,79	918,32	761,86	9750,58	3641,16	1150,845	6695,87	-5545,025
**CERS6**	6736	3665,69	3079,28	5843,47	6972,26	13766,3	4831,11	10369,28	-5538,17
**TAX1BP1**	3302,48	4646,21	4810,34	4808,04	9743,62	10110,97	4391,7675	9927,295	-5535,5275
**ERO1A**	3184,95	6743,75	2836,39	4120,76	13664,34	5843,57	4221,4625	9753,955	-5532,4925
**ERBIN**	3413,44	5220,7	2117,16	3934,75	6466,51	11940,12	3671,5125	9203,315	-5531,8025
**TBC1D30**	8,3	23,84	69,73	50,11	11083,03	49,06	37,995	5566,045	-5528,05
**LNPEP**	3058,16	2964,38	2333,59	3200,2	8331,94	8487,65	2889,0825	8409,795	-5520,7125
**ROCK1**	7153,02	5683,09	6938,61	5814,38	11856,64	11965,88	6397,275	11911,26	-5513,985
**LSM8**	2813,39	3547,51	4694,68	1876,53	13895,32	3597,2	3233,0275	8746,26	-5513,2325
**PRDX3**	9473,2	11041,38	5159,12	7898,82	18451,49	9352,38	8393,13	13901,935	-5508,805
**NIN**	2771,2	2587,88	581,06	970,52	5410,85	9034,89	1727,665	7222,87	-5495,205
**SERINC1**	3837,19	5247,99	4839,54	8057,5	11497,63	10462,12	5495,555	10979,875	-5484,32
**ALDOC**	7637,94	4329,04	7286,29	2074,82	18611,83	3017,75	5332,0225	10814,79	-5482,7675
**RBPJ**	5881	4947,62	3014,92	3017,19	12658,91	6731,03	4215,1825	9694,97	-5479,7875
**DCP2**	5955,4	6590,19	4460,08	4167,53	8259,88	13277,13	5293,3	10768,505	-5475,205
**SATB2**	42,92	4,02	50,07	29,87	10997,43	0	31,72	5498,715	-5466,995
**RALGAPB**	1666,24	3181,22	2707,5	4808,9	2655,34	14424,76	3090,965	8540,05	-5449,085
**MKX**	0	0	25,79	3,65	10885,81	0	7,36	5442,905	-5435,545
**ARF6**	9308,78	10059,98	5974,58	9018,55	20303,32	7729,72	8590,4725	14016,52	-5426,0475
**SRGAP2**	3165,06	2897,3	878,42	4403,35	10719,67	5796,15	2836,0325	8257,91	-5421,8775
**AKNA**	2670,73	2386,89	3344,61	1632,36	12163,88	3694,59	2508,6475	7929,235	-5420,5875
**ZNF217**	3308,86	3148,67	1735,59	6650,51	10852,46	7399,67	3710,9075	9126,065	-5415,1575
**STMN1**	29352,25	34672,8	46972,4	29327,62	25698,07	55277,8	35081,2675	40487,935	-5406,6675
**TENT5A**	1330,2	295,29	1641,7	3292,92	10542,65	3501,65	1640,0275	7022,15	-5382,1225
**FOS**	56,93	563,92	3272,46	169,91	12532,16	243,27	1015,805	6387,715	-5371,91
**RBM25**	13995,75	14819,32	15034,09	11152,61	27232,74	10979,38	13750,4425	19106,06	-5355,6175
**PRRC2C**	37768,75	21954,36	26090,96	27534,45	38341,06	29006,28	28337,13	33673,67	-5336,54
**TBL1XR1**	6183,28	4300,48	4497,93	4454,73	10199,78	10134,36	4859,105	10167,07	-5307,965
**BRD3**	5599,98	6700,11	2907,31	5755,37	3204,91	17877,22	5240,6925	10541,065	-5300,3725
**FRAT2**	2326,53	3227,85	2680,57	3144,23	13645,75	2638,9	2844,795	8142,325	-5297,53

**MAP3K1**	2343,33	1701,81	1109,33	1608,12	8419,07	5553,06	1690,6475	6986,065	-5295,4175
**GPCPD1**	4025,41	1812,93	2196,52	1274,14	13176,63	2060,77	2327,25	7618,7	-5291,45
**NDUFS2**	7500,45	9734,9	11312,98	7573,5	23711,79	4900,97	9030,4575	14306,38	-5275,9225
**SMN1**	4410,12	3942,72	2620,11	3857,23	14088,95	3855,38	3707,545	8972,165	-5264,62
**TMX4**	2699,21	3331,87	2057,61	4077,12	3516,05	13080,99	3041,4525	8298,52	-5257,0675
**BEX1**	513,57	31,68	2215,9	1,13	11889,67	0	690,57	5944,835	-5254,265
**TASL**	356,23	15,09	91,83	86,65	10376,59	400,2	137,45	5388,395	-5250,945
**TMEM30A**	5901,6	4970,49	5551,72	6830,62	13757,9	8366,54	5813,6075	11062,22	-5248,6125
**USP32**	3596,26	4459,87	5010,3	5849,8	17220,07	2733,95	4729,0575	9977,01	-5247,9525
**TMX1**	6772,42	6078,69	2192,65	5637,05	7195,07	13618,17	5170,2025	10406,62	-5236,4175
**ATP2B4**	11238,74	8770,55	10724,76	13589,84	9278,12	23340,23	11080,9725	16309,175	-5228,2025
**LAMP2**	6712,79	7422,85	5979,92	6408,67	13702,77	9968,58	6631,0575	11835,675	-5204,6175
**SLC2A4RG**	3098,21	5036,02	5348,29	3655,13	13699,95	5276,94	4284,4125	9488,445	-5204,0325
**SLC25A1**	6584,04	4276,31	3422,92	2913,95	13221,81	5779,43	4299,305	9500,62	-5201,315
**CDKN1B**	3535,45	2893,73	1730,08	3140,32	9618,25	6418,39	2824,895	8018,32	-5193,425
**SQLE**	15251,83	15364,79	2693,53	4679,69	16493,91	12879,02	9497,46	14686,465	-5189,005
**SMARCA5**	12881,67	10344,36	6273,96	8161,03	10058,62	19143,42	9415,255	14601,02	-5185,765
**MVD**	3636,19	2137,23	1787,85	1898,23	4135,81	10951,74	2364,875	7543,775	-5178,9
**ARL6IP1**	15453,37	12677,92	9998,77	18313,15	17649,3	20927,33	14110,8025	19288,315	-5177,5125
**SNTB1**	28,09	102,72	187,49	1778,02	9280,6	2087,52	524,08	5684,06	-5159,98
**SRSF6**	11543,59	36073,58	13620,47	17869,11	24874,37	24985,01	19776,6875	24929,69	-5153,0025
**WDR26**	7297,61	7774,5	5469,4	4629,89	16279,92	6577,15	6292,85	11428,535	-5135,685
**IARS1**	13510,74	22376,84	14315,32	25952,8	12273,93	36052,35	19038,925	24163,14	-5124,215
**MFSD10**	6558,38	9612,86	9610,62	5884,79	22280,69	3793,06	7916,6625	13036,875	-5120,2125
**P4HB**	62832,58	55448,31	106115,52	79019,39	140129,87	21780,05	75853,95	80954,96	-5101,01
**OSBPL8**	4304,34	7602,95	2237,46	5707,57	4576,54	15516,07	4963,08	10046,305	-5083,225
**ZNF638**	10957,45	7703,92	14777,07	8601,63	24217,4	6941,66	10510,0175	15579,53	-5069,5125
**VPS13C**	3284,36	4253,14	4040,87	4311,29	5730,11	12326,18	3972,415	9028,145	-5055,73
**GOLM1**	629,37	331,65	609,1	435,82	4731,56	6351,34	501,485	5541,45	-5039,965
**SIRPA**	12,16	2,14	6,64	0	8180,68	1894,04	5,235	5037,36	-5032,125
**UGP2**	3312,26	4202,19	3881,49	4226,5	12245,31	5603,15	3905,61	8924,23	-5018,62
**RPL17**	81552,48	74073,63	78961,94	70787,07	134526,59	28099,13	76343,78	81312,86	-4969,08
**UQCRB**	12016,76	8086,25	16893,84	8874,49	28838,08	3980,19	11467,835	16409,135	-4941,3

**ZNF516**	595,87	4,01	1313,09	218,35	8054	2889,2	532,83	5471,6	-4938,77
**ASXL1**	6315,31	8091,25	7087,95	11113,27	7240,44	18925,4	8151,945	13082,92	-4930,975
**CSF3R**	220,4	112,87	1045,44	106,67	10438,1	157,68	371,345	5297,89	-4926,545
**THBD**	70,72	89,77	214,89	9	9777,68	256,03	96,095	5016,855	-4920,76
**CEP70**	1561,93	2388,16	2854,54	1428,25	335,48	13618	2058,22	6976,74	-4918,52
**POGLUT3**	310,45	2191,58	1769,26	3583,84	2930,66	10831,33	1963,7825	6880,995	-4917,2125
**SUZ12**	8193,19	6222,57	3640,08	5560,81	10899,92	10696,34	5904,1625	10798,13	-4893,9675
**CLTCL1**	15,9	89,98	67,42	158,38	9446,6	472,44	82,92	4959,52	-4876,6
**PPP4R2**	2142,8	6723,64	2404,43	2049,94	7749,99	8650,39	3330,2025	8200,19	-4869,9875
**MT-ND2**	224953,3	157576,4	115856,07	153290,5	152442,92	183113,8	162919,065	167778,35	-4859,285
**CTDSPL2**	3261,85	3540,15	1190,34	3655,09	5334,8	10206,26	2911,8575	7770,53	-4858,6725
**SH3KBP1**	3244,16	2416,54	3825,71	4263,16	14924,07	1648,4	3437,3925	8286,235	-4848,8425
**CHAMP1**	6622,85	4236,12	5951,7	3478,22	2339,83	17499,83	5072,2225	9919,83	-4847,6075
**IGF1R**	4286,69	2707,7	4698,77	2165,85	144,55	16476,3	3464,7525	8310,425	-4845,6725
**NUCB2**	5356,84	10345,13	19648,7	8539,01	13434,53	18198,77	10972,42	15816,65	-4844,23
**EXOC3**	3113,38	6948,56	7939,71	4929,29	17693,32	3414,84	5732,735	10554,08	-4821,345
**NR3C1**	1672,52	1228,81	913,12	1740,19	6082,02	6333,41	1388,66	6207,715	-4819,055
**CCDC14**	8649,48	7236,16	6183,5	5180,11	4597,16	18660,15	6812,3125	11628,655	-4816,3425
**YY1**	10758,02	10660,03	5145	9833,98	10498,51	17332,14	9099,2575	13915,325	-4816,0675
**ZCCHC14**	995,05	1648,41	1220,81	663,74	1363,31	10520,34	1132,0025	5941,825	-4809,8225
**GPBP1**	7486,09	5813,63	6815,25	3739,42	12875,95	8657,53	5963,5975	10766,74	-4803,1425
**ZFP36**	487,65	1151,46	2480,61	2008,03	11012,42	1652,71	1531,9375	6332,565	-4800,6275
**LUC7L2**	10414,82	7224,1	7592,89	6237,06	17589,26	7745,5	7867,2175	12667,38	-4800,1625
**SEPTIN9**	7152,43	20430,07	16525,58	2674,93	19354,18	13633,14	11695,7525	16493,66	-4797,9075
**PAPOLA**	14906,06	12119,2	7850,24	12666,08	16178,73	17171,57	11885,395	16675,15	-4789,755
**PCNX4**	5445,1	5998,47	2711,97	3731,04	4755,12	13727,37	4471,645	9241,245	-4769,6
**CLEC11A**	10731,81	34390,92	3023,72	32828,61	37182,07	12841,3	20243,765	25011,685	-4767,92
**DCTN4**	2928,62	4271,54	2782,16	4467,65	8191,92	8564,43	3612,4925	8378,175	-4765,6825
**IFI16**	1751,26	4546,52	6713,65	687,1	4250,06	12121,17	3424,6325	8185,615	-4760,9825
**UTP6**	5614,73	4411,21	7553,94	5151,54	18056,95	2822,96	5682,855	10439,955	-4757,1
**MYO18A**	1913,81	13179,49	1758,92	1225,64	4282,57	14252,51	4519,465	9267,54	-4748,075
**GLUD1**	11668,02	10518,62	6541,19	8506,51	14160,65	13947,75	9308,585	14054,2	-4745,615
**MGST1**	2323,29	551,44	3783,71	1487,45	13562,47	0	2036,4725	6781,235	-4744,7625

**NXPE3**	527,3	1834,72	1205,13	801,94	8892,64	2780,57	1092,2725	5836,605	-4744,3325
**MS4A7**	0,72	1,24	0	12,9	9307,02	166,54	3,715	4736,78	-4733,065
**MYCBP2**	24927,2	9494,22	32134,44	15610,24	41816,53	8724,2	20541,525	25270,365	-4728,84
**ARPC3**	14193,23	13823,2	18555,23	16606,54	31483,42	9553,46	15794,55	20518,44	-4723,89
**DCAF12**	6008,62	5053,06	3551,17	5865,71	7768,32	11909,2	5119,64	9838,76	-4719,12
**HIPK2**	5561,69	3578,13	3562,34	3711,33	5166,59	12476,8	4103,3725	8821,695	-4718,3225
**RESF1**	3978,09	3841,61	1738,2	2850,6	5897,02	9725,81	3102,125	7811,415	-4709,29
**GIGYF1**	6759,46	10876,27	6492,85	6306,47	15371,87	9261,36	7608,7625	12316,615	-4707,8525
**DENND3**	1642,06	5713,18	1462,7	2515,49	12040,9	3041,22	2833,3575	7541,06	-4707,7025
**FN1**	30,85	43,33	58,13	13,19	9368,15	109,42	36,375	4738,785	-4702,41
**PRKCD**	2977,91	4466,57	2328,33	3340,38	15951,65	6,62	3278,2975	7979,135	-4700,8375
**SLAIN1**	34,1	612,24	2572,23	916,88	11460,8	0	1033,8625	5730,4	-4696,5375
**MTAP**	0	4869,33	2414,77	0	4506,05	8527,12	1821,025	6516,585	-4695,56
**PDS5A**	15174,55	12977,48	5084,41	10392,7	15298,24	15907,23	10907,285	15602,735	-4695,45
**CD47**	9962,75	7986,67	3354,38	14427,81	4319,67	22913,42	8932,9025	13616,545	-4683,6425
**DARS1**	9519	12558,71	5846,34	9832,43	17398,52	10827,6	9439,12	14113,06	-4673,94
**RTN4**	11957,91	10460,06	10956,15	15222,23	18939,07	14675,65	12149,0875	16807,36	-4658,2725
**FUT4**	3052,25	1323,88	1575,84	3765,42	11287,15	2862,6	2429,3475	7074,875	-4645,5275
**AHRR**	11,38	2495,34	110,31	430,23	9343,33	1457,78	761,815	5400,555	-4638,74
**IPO7**	16799,71	13910,35	8368,48	15640,34	16380,21	20239,13	13679,72	18309,67	-4629,95
**SPN**	64126,48	80407,14	22949,49	65827,06	15376,98	110531,5	58327,5425	62954,215	-4626,6725
**TLE5**	11404,01	15423,04	17084,29	5966,07	18441,63	15734,38	12469,3525	17088,005	-4618,6525
**VOPP1**	3532,66	58,06	1111,23	11,27	2377,25	9155,84	1178,305	5766,545	-4588,24
**KIF15**	5410,85	3614,34	3521,12	2350,39	7700,63	8923,36	3724,175	8311,995	-4587,82
**ADNP**	10094,31	9014,24	10341,77	9859,39	13116,44	15710,8	9827,4275	14413,62	-4586,1925
**CBL**	1294,22	3500,84	339,47	1968,58	4900,07	7804,42	1775,7775	6352,245	-4576,4675
**YWHAZ**	21897,14	30380,12	21860,01	29574,39	35883,48	25122,48	25927,915	30502,98	-4575,065
**SLC30A1**	1217,19	998	650,17	1530,47	8921,32	2408,22	1098,9575	5664,77	-4565,8125
**CAT**	7963,67	4152,44	9164,4	10418,81	10533,37	14444,8	7924,83	12489,085	-4564,255
**PCNX1**	1557,61	325,42	2273,31	724,62	8716,66	2840,77	1220,24	5778,715	-4558,475
**CD70**	12,01	3091,96	13,24	37,85	10500,67	181,42	788,765	5341,045	-4552,28
**ORAI3**	826,15	3807,58	3081,61	2085,9	7720,59	6280,42	2450,31	7000,505	-4550,195
**LRPPRC**	28157,75	28491,28	17981,67	23396	48712,49	9393,89	24506,675	29053,19	-4546,515

**KCTD12**	327,94	23,38	727,31	401,75	8183,3	1627,91	370,095	4905,605	-4535,51
**AHNAK**	3665,79	16069,55	11625,26	31315,01	26604,03	13793,84	15668,9025	20198,935	-4530,0325
**MDH1**	8672,09	7266,1	8000,97	9914,34	14990,32	10975,44	8463,375	12982,88	-4519,505
**SESN3**	1138,56	801,3	2439,84	1228,49	11243,78	598,68	1402,0475	5921,23	-4519,1825
**ERLIN1**	3357,33	3462,89	1586,54	3200,97	8855,82	5975,33	2901,9325	7415,575	-4513,6425
**HAL**	28,77	5,08	56,21	77,28	9060,63	33,42	41,835	4547,025	-4505,19
**CMTM3**	1338,52	4646,79	2553,88	1358,04	3598,42	10347,03	2474,3075	6972,725	-4498,4175
**DGKD**	3013,41	6990,72	1982,06	3070,95	10429,2	6082,73	3764,285	8255,965	-4491,68
**EIF4EBP2**	8718,31	11545,29	5904,79	5829,31	8951,13	16015,18	7999,425	12483,155	-4483,73
**INSR**	130,38	2819,13	1063,81	867,69	4606,8	6784,91	1220,2525	5695,855	-4475,6025
**FADS1**	20524,52	11202,2	8773,45	12105,38	11489,77	23694,62	13151,3875	17592,195	-4440,8075
**RAP2A**	3156,89	7355,41	4261,63	2163,82	2893,85	14431,27	4234,4375	8662,56	-4428,1225
**TXN**	10675,73	8432,53	8950,54	11650,61	15564,13	13129,96	9927,3525	14347,045	-4419,6925
**ZBTB33**	3077,17	3061,07	2425,78	4432,41	8582,15	6754,05	3249,1075	7668,1	-4418,9925
**IL6R**	767,49	73,94	1456,6	808,26	10306,65	75,1	776,5725	5190,875	-4414,3025
**UNC93B1**	1598,9	1736,92	2241,91	2600,52	12634,33	281,19	2044,5625	6457,76	-4413,1975
**HSD17B11**	5312,3	4141,9	2729,08	5939,9	10753,68	7132,42	4530,795	8943,05	-4412,255
**NCK2**	1038,87	3204	548,07	192,24	8088,69	3214,49	1245,795	5651,59	-4405,795
**MOB3A**	3232,06	8072,27	1357,37	3002,72	10860,17	5766,7	3916,105	8313,435	-4397,33
**EIF3M**	24120,36	9696,92	19030,07	29624,69	29670,87	20356,2	20618,01	25013,535	-4395,525
**SMCHD1**	6767,97	6141,34	1848,65	7012,31	8011,47	11660,27	5442,5675	9835,87	-4393,3025
**SMG1**	8360,89	14086,79	3853,62	9735,89	6881,25	19910,18	9009,2975	13395,715	-4386,4175
**GAA**	962,79	568,93	1230,17	284,31	8480,52	1797,7	761,55	5139,11	-4377,56
**TOR1AIP1**	5028,15	2418,51	1914,63	4039,16	10297,14	5125,32	3350,1125	7711,23	-4361,1175
**CSGALNACT2**	1229,56	497,87	689,66	1790,43	7710,85	3112,51	1051,88	5411,68	-4359,8
**PHACTR3**	3,01	0	20,18	2,75	8270,26	454,83	6,485	4362,545	-4356,06
**HMGB3**	4937,2	6346,13	5350,41	2671,55	9368,62	8974,53	4826,3225	9171,575	-4345,2525
**CHCHD7**	1696,79	1973,18	2493,45	452,53	9775,2	2211,12	1653,9875	5993,16	-4339,1725
**HDAC5**	1262,33	4269,85	1580,65	1592,52	4291,26	8734,36	2176,3375	6512,81	-4336,4725
**ZNF106**	4066,89	8409,14	2722,82	5352,09	11438,07	7501,58	5137,735	9469,825	-4332,09
**C5orf15**	1501,82	1437,03	562,01	1027,44	5934,9	4977,22	1132,075	5456,06	-4323,985
**CAPZA1**	20102,59	12183,46	19110,22	25046,89	28837,11	18029,31	19110,79	23433,21	-4322,42
**HOXA11**	57,01	1,79	604,12	2,68	7334,68	1621,69	166,4	4478,185	-4311,785

**WAC**	9377,93	10128,26	9049,79	11557,1	13758,71	14919,63	10028,27	14339,17	-4310,9
**IMPDH2**	19623,02	58525,67	19002,73	20140,99	56245,53	11009,87	29323,1025	33627,7	-4304,5975
**ANXA2**	3075,09	4897,5	16831,31	20040,05	28770,66	2258,68	11210,9875	15514,67	-4303,6825
**RICTOR**	2018,99	2366,48	2711,48	3272,48	8957,87	4829,73	2592,3575	6893,8	-4301,4425
**CLN3**	3176,01	3199,27	2250,47	3918,27	3586,32	11280,27	3136,005	7433,295	-4297,29
**CUL4A**	14030,21	8734,01	22643,78	10231,41	8420,23	27988,22	13909,8525	18204,225	-4294,3725
**TRIM56**	5312,84	7832,16	8444,09	5968,66	11378,26	10983,76	6889,4375	11181,01	-4291,5725
**UBE2J1**	4044,91	6371,97	2866,07	6324,46	10939,37	7417,77	4901,8525	9178,57	-4276,7175
**NEMP1**	3894,9	3684,86	1735,84	5581,33	3894,28	12094,58	3724,2325	7994,43	-4270,1975
**CPXM1**	2665,08	2419,95	8428,33	354,48	442,63	15030,4	3466,96	7736,515	-4269,555
**PRKAR2B**	2556,36	12333,58	2595,17	4631,94	4122,81	15471,67	5529,2625	9797,24	-4267,9775
**IARS2**	15988,73	8376,27	7315,33	6418,99	19758,61	7824,09	9524,83	13791,35	-4266,52
**CCNB1IP1**	4902,57	4579,75	3749,48	3630,5	11772,33	5183,06	4215,575	8477,695	-4262,12
**TOP1MT**	474,21	10400,48	942,78	6700,34	17272,08	486,9	4629,4525	8879,49	-4250,0375
**STARD4**	3814,51	1654,38	856,76	1140,82	5307,77	6888,77	1866,6175	6098,27	-4231,6525
**HCLS1**	10023,38	304,13	20926,47	11971,46	23360,6	6683,99	10806,36	15022,295	-4215,935
**TIAL1**	6908	5745,33	4522,46	4489	11953,04	7309,77	5416,1975	9631,405	-4215,2075
**SREK1**	8804,62	6790,41	4844,73	3600,84	11211,03	9230,58	6010,15	10220,805	-4210,655
**FANCA**	6195,01	4056,03	1790,37	2205,49	5076,3	10460,99	3561,725	7768,645	-4206,92
**ATL3**	2865,51	2508,25	1577,12	2407,99	8174,93	4915,68	2339,7175	6545,305	-4205,5875
**NUFIP2**	8075,12	8516,19	5182,38	9162,78	10444,9	13433,32	7734,1175	11939,11	-4204,9925
**BMP8B**	784,06	33,95	908,22	598,69	9550,94	17,48	581,23	4784,21	-4202,98
**PRPF8**	23773,34	38603,58	32589,87	29237,82	49882,08	20614,16	31051,1525	35248,12	-4196,9675
**KDM3A**	7969,46	5353,97	3936,6	6846,83	13567,87	6867,08	6026,715	10217,475	-4190,76
**CHCHD3**	8676,44	5210,65	4559,58	7301,76	14320,33	6925,56	6437,1075	10622,945	-4185,8375
**NLRP3**	337,11	50,7	34,8	47,1	8124,87	475,09	117,4275	4299,98	-4182,5525
**PHGDH**	3280,58	1,44	8038,63	784,09	2299,27	12115,82	3026,185	7207,545	-4181,36
**RNF114**	4273,58	6212,36	4774,25	7284,22	11994,55	7638,73	5636,1025	9816,64	-4180,5375
**FCGR2B**	40,71	8,35	248,5	930,02	8950,8	5,8	306,895	4478,3	-4171,405
**MRPL33**	1202,28	1299,54	2418,92	638,45	10623	494,08	1389,7975	5558,54	-4168,7425
**CBWD2**	2273,13	2537,78	1807,21	2024,67	9088,52	3559,89	2160,6975	6324,205	-4163,5075
**RHEX**	60,48	28,67	55,95	1030,98	484,91	8427,23	294,02	4456,07	-4162,05
**PIK3CD**	564,58	750,47	932,34	4048,34	8795,32	2670,76	1573,9325	5733,04	-4159,1075

**SAMHD1**	445,44	736,59	1036,3	1144,04	9922,11	62,4	840,5925	4992,255	-4151,6625
**PJA2**	7153,82	8293,78	3556,1	7942,48	8507,58	13263,58	6736,545	10885,58	-4149,035
**PHF2**	1749,41	3589,72	1297,66	2213,02	2547,03	10169,25	2212,4525	6358,14	-4145,6875
**STK4**	5088,02	4337,26	3179,28	5011,15	5759,93	11332,01	4403,9275	8545,97	-4142,0425
**MT-ND3**	26119,58	24223,6	33647,21	18839,47	34116,19	25569,42	25707,465	29842,805	-4135,34
**MFSD14B**	2842,39	5531,27	3659,38	6484,4	5499,98	11992,86	4629,36	8746,42	-4117,06
**APAF1**	1840,77	1772,78	1786,33	2910,11	7451,98	4930,4	2077,4975	6191,19	-4113,6925
**ALDH9A1**	5379,67	3309,32	2933,11	3745,16	12344,95	3561,65	3841,815	7953,3	-4111,485
**WSB1**	7756,23	11484,11	6289,31	5714,71	14951,54	8891,08	7811,09	11921,31	-4110,22
**CLSTN2**	0	0	0	0	8202,27	11,36	0	4106,815	-4106,815
**BAZ2A**	7390,93	10366,19	5307,15	7843,81	8965,18	14686,57	7727,02	11825,875	-4098,855
**BTBD2**	3565,83	9291,21	8214,2	3430,04	12800,1	7642,44	6125,32	10221,27	-4095,95
**KMT2E**	5409,48	4737,21	4699,87	3689,34	11413,87	6040,94	4633,975	8727,405	-4093,43
**EEF1B2**	45398,69	51898,31	39239,24	34015,29	74631,16	18828,69	42637,8825	46729,925	-4092,0425
**SLC2A5**	6,5	16,77	3,31	0	8162,54	29,97	6,645	4096,255	-4089,61
**ANLN**	4456,91	2834,72	1717,97	2879,56	6522,61	7595,29	2972,29	7058,95	-4086,66
**THOC2**	13430,49	10719,9	25347,55	11015,52	28818,3	9607,19	15128,365	19212,745	-4084,38
**PHIP**	7970,5	5070,72	8295,18	9397,79	12593,37	10910,39	7683,5475	11751,88	-4068,3325
**FTO**	2063,06	2132,48	915,63	1560,91	1617,72	9841,35	1668,02	5729,535	-4061,515
**RASSF4**	23,53	241,96	68,58	19,14	8184,23	108,27	88,3025	4146,25	-4057,9475
**PAN2**	2075,48	3412,84	2880,73	2906,18	5057,64	8690,31	2818,8075	6873,975	-4055,1675
**PPP1R16B**	398,77	3330,79	968,23	983,9	519,97	10389,11	1420,4225	5454,54	-4034,1175
**ST3GAL3**	1583,82	1483,6	3296,83	1856,46	9890	2286,34	2055,1775	6088,17	-4032,9925
**AKAP9**	5841,59	6824,24	8932,1	5480,11	15388,38	6202,96	6769,51	10795,67	-4026,16
**GTF3C4**	3964,88	5310,21	2394,49	3938,28	3056	12786,28	3901,965	7921,14	-4019,175
**GRSF1**	11360,43	9551,89	6914,12	9307,33	20042,69	6537,73	9283,4425	13290,21	-4006,7675
**TIMP2**	5,49	4,26	1,9	0	4966,99	3052,02	2,9125	4009,505	-4006,5925
**RAD50**	5488,48	4441,41	2074,02	4286,12	10582,89	5574,71	4072,5075	8078,8	-4006,2925
**TMED10**	8752,98	8325,3	9013,72	10796,55	12351,27	14096,44	9222,1375	13223,855	-4001,7175
**RPL5**	112323,4	109765,7	139051,23	110524,6	169766,95	74068,66	117916,228	121917,805	-4001,5775
**PCNP**	8543,66	11174,77	4868,82	8264,02	11230,92	13196,2	8212,8175	12213,56	-4000,7425
**ONECUT2**	80,44	15,09	207,54	276,84	8289,89	0	144,9775	4144,945	-3999,9675
**RAPGEF1**	1879,25	3039,25	196,68	2241,76	3590,9	8084,93	1839,235	5837,915	-3998,68

**SLC25A36**	5872,02	6913,32	3424,02	2915,57	7861,18	9681,19	4781,2325	8771,185	-3989,9525
**PPP2R5C**	5904,12	5958,25	5516,37	5093,69	9388,69	9806,96	5618,1075	9597,825	-3979,7175
**CKS1B**	11246,88	5752,03	7818,08	9327,34	16944,22	8084,73	8536,0825	12514,475	-3978,3925
**IL1RN**	1,81	1,55	5,65	69,43	7991,54	1,79	19,61	3996,665	-3977,055
**CDH2**	4,02	3,03	34,47	0	7,27	7957,62	10,38	3982,445	-3972,065
**TAOK1**	3594,48	3650,53	2494,94	3207,95	5435,37	8963,33	3236,975	7199,35	-3962,375
**ACSL3**	6306,57	3296,63	3612,38	5123,15	4364,13	12720,85	4584,6825	8542,49	-3957,8075
**SERPINB10**	1,5	1,28	0	0	7909,1	4,44	0,695	3956,77	-3956,075
**SPI1**	6463,67	2232,35	7176,64	10883,04	19454,33	1831,65	6688,925	10642,99	-3954,065
**VSIR**	6389,13	6074,76	5862,55	6301,95	15642,12	4579,64	6157,0975	10110,88	-3953,7825
**PTPRE**	311,68	2424,49	27,45	1900,46	8450,27	1757,42	1166,02	5103,845	-3937,825
**HMMR**	5990,72	4461,47	1527,92	3462,18	5109,57	10485,37	3860,5725	7797,47	-3936,8975
**BRI3**	1548,85	1135,42	1996,79	1200,54	9488,67	1324,83	1470,4	5406,75	-3936,35
**MMS19**	7001,11	7845,03	8739,95	6587,63	18188,4	4768,42	7543,43	11478,41	-3934,98
**LCP1**	25282,72	33649,79	26745,03	21931,54	53520,54	8141,97	26902,27	30831,255	-3928,985
**KLHL9**	0	3239,92	1748,48	0	1905,13	8443,62	1247,1	5174,375	-3927,275
**NFATC4**	167,15	2203,03	1651,48	7,54	184,3	9670,5	1007,3	4927,4	-3920,1
**EXOC5**	2857,12	4532,54	931,8	3895,64	2468,43	11438,24	3054,275	6953,335	-3899,06
**SDC2**	0	32,39	0	4,66	7808,45	0	9,2625	3904,225	-3894,9625
**UHRF1BP1L**	1257,67	1170,88	1273,65	2026,37	8896,32	1756,12	1432,1425	5326,22	-3894,0775
**ACTN1**	16394,39	21983,04	12990,36	16446,56	22596,08	19068,24	16953,5875	20832,16	-3878,5725
**PGM1**	418,26	1160,44	2758,55	5,63	6710,95	3213,06	1085,72	4962,005	-3876,285
**MSMO1**	3645,87	666,61	471,01	1470,95	5339,29	5521,17	1563,61	5430,23	-3866,62
**FOXP4**	2605,56	2825,29	3387,3	1455	7125,09	5730,92	2568,2875	6428,005	-3859,7175
**GNB2**	16222,91	13530,35	19056,35	14471,04	28510,8	10842,57	15820,1625	19676,685	-3856,5225
**APOBR**	3621,4	4913,22	4690,86	6058,3	12920,03	4424,31	4820,945	8672,17	-3851,225
**TMEM38B**	1018,56	862,41	530,2	1628,98	3180,4	6535,77	1010,0375	4858,085	-3848,0475
**ITPRID2**	8972,25	5665,58	6714,06	6443,94	8220,85	13370,19	6948,9575	10795,52	-3846,5625
**SHOC2**	2533,63	2350,25	1470,99	2971,1	7055,55	5291,83	2331,4925	6173,69	-3842,1975
**FCGR1A**	3,09	1,32	82,62	7,86	7730,47	0	23,7225	3865,235	-3841,5125
**NFATC2IP**	5486	6015,17	3266,4	6061,59	6044,23	12037,76	5207,29	9040,995	-3833,705
**PPP3CA**	6389,39	4258,08	4055,04	8068,95	7982,3	11065,17	5692,865	9523,735	-3830,87
**NCS1**	47,09	1794,92	142,86	56,76	8662	0	510,4075	4331	-3820,5925

**FOXN3**	2449,85	6955,44	2578,59	2046,97	6128,81	8521,74	3507,7125	7325,275	-3817,5625
**RACGAP1**	4151,76	5287,24	2273,46	5445,01	6035,41	10177,63	4289,3675	8106,52	-3817,1525
**ARHGAP19**	5029,51	4385,44	2507,24	2182,88	8415,76	6265	3526,2675	7340,38	-3814,1125
**TMED8**	3201,63	3648,02	3469,41	1734,16	5689,19	7950,79	3013,305	6819,99	-3806,685
**KIF2A**	7286,2	8788,73	3372,91	4086,84	5418,75	13953,97	5883,67	9686,36	-3802,69
**IPO9**	18798,37	9381,83	9145,58	10544,47	21811,63	9716,37	11967,5625	15764	-3796,4375
**DNM1**	397,92	1130,86	969,61	112,36	350,93	8545,23	652,6875	4448,08	-3795,3925
**PAK2**	9791	15406,17	7072,95	11098,62	11098,6	18173,84	10842,185	14636,22	-3794,035
**ARHGAP5**	825,35	2241,12	912,81	2,21	44,21	9532,94	995,3725	4788,575	-3793,2025
**ATG13**	3907,09	5955,42	8972,62	5726,28	13626,21	6233,51	6140,3525	9929,86	-3789,5075
**HENMT1**	2726,45	1,09	7301,08	62,7	2169,38	10454,09	2522,83	6311,735	-3788,905
**CDK5RAP3**	4969,69	8336,78	8813,69	8068,76	11447,63	11220,59	7547,23	11334,11	-3786,88
**SYPL1**	7627,84	4589,44	6143,11	5692,57	10900,4	8699,58	6013,24	9799,99	-3786,75
**PTPN12**	4812,42	3792,04	2354,89	3153,49	9532,88	5089,34	3528,21	7311,11	-3782,9
**TUBA8**	8,5	37,12	135,16	19,81	2874,5	4787,87	50,1475	3831,185	-3781,0375
**SPARC**	2543,27	1060,44	3639,54	522,53	6,03	11435,08	1941,445	5720,555	-3779,11
**NUMB**	2687,13	5279,22	2097,73	2037,46	6837,67	6746,51	3025,385	6792,09	-3766,705
**BSG**	27301,54	30018,86	44791,62	8125,91	41691,18	20957,85	27559,4825	31324,515	-3765,0325
**TM9SF3**	9248,86	10626,17	4575,93	9345,5	7588,94	16838,11	8449,115	12213,525	-3764,41
**HEBP2**	864,1	1295,99	1393,1	3493,86	9908,82	1140,8	1761,7625	5524,81	-3763,0475
**ZNF292**	7515,23	3282,9	6852,64	8787,66	12508,48	8234,7	6609,6075	10371,59	-3761,9825
**PAN3**	3332,34	3807,44	2034,45	2722,42	9735,18	3735,48	2974,1625	6735,33	-3761,1675
**AP1S2**	5176,51	3106,12	1851,65	4684,55	10894,24	4034,23	3704,7075	7464,235	-3759,5275
**PTBP1**	39904,11	53582,78	22657,85	28541,87	49270,32	30558,79	36171,6525	39914,555	-3742,9025
**ABCA2**	1893,03	6575,9	3600,81	3002,8	10250,66	4763,31	3768,135	7506,985	-3738,85
**DCK**	6261,8	2209,37	2554,27	5762,26	4154,68	11716,54	4196,925	7935,61	-3738,685
**NUF2**	3931,89	2082,88	1908,33	3421,85	7749,5	5400,26	2836,2375	6574,88	-3738,6425
**AVL9**	2585,84	3527,8	3118,24	2968,07	6461,29	7097,85	3049,9875	6779,57	-3729,5825
**ARPC5**	15087,51	10785,49	6350,85	17134,18	13229,07	18897,39	12339,5075	16063,23	-3723,7225
**BNIP3**	13508,61	17580,88	9769,86	7392,97	31340,35	231,16	12063,08	15785,755	-3722,675
**DAPK1**	3272,61	10859,89	6629,83	10359,66	4722,26	18276,86	7780,4975	11499,56	-3719,0625
**FEM1B**	3321,92	3692,66	1661,57	3500,32	7181,15	6335,19	3044,1175	6758,17	-3714,0525
**FAM168A**	2836,73	3099,85	2141	2006,33	5013,09	7453,93	2520,9775	6233,51	-3712,5325

**TUG1**	9776,39	11425,34	6993	7571,35	6905,08	18391,07	8941,52	12648,075	-3706,555
**CPD**	3319,01	3044,38	2669,81	2311,88	5425,77	7656,39	2836,27	6541,08	-3704,81
**CLSTN1**	6997,45	3150,1	6719,37	8397,01	10044,56	9982,08	6315,9825	10013,32	-3697,3375
**CDC7**	4795,97	2314,64	1814,77	3428,63	1996,1	11570,94	3088,5025	6783,52	-3695,0175
**DNAJC13**	3651,27	3180,4	4225,8	3383,44	10809,26	3789,5	3610,2275	7299,38	-3689,1525
**KDM5B**	10671,84	13678,68	10027,39	6995,92	14709,42	13322,05	10343,4575	14015,735	-3672,2775
**AC098850.3**	304,08	598,95	343,45	629,07	3736,19	4543,81	468,8875	4140	-3671,1125
**AP3S1**	2785,49	2614,02	3324,47	2405,97	8497,16	4409,12	2782,4875	6453,14	-3670,6525
**GPT2**	1124,09	1448,04	3072,75	5452,74	4146,95	8741,74	2774,405	6444,345	-3669,94
**FLT3**	0	0	3,78	0	6626,27	710,88	0,945	3668,575	-3667,63
**EVI2A**	53,34	459,07	41,71	0	7435,32	170,55	138,53	3802,935	-3664,405
**RASA1**	2718,14	7348,45	1669,85	1799,16	8698,83	5392,92	3383,9	7045,875	-3661,975
**SRGAP2C**	1318,65	679,42	614,1	1489,9	7898,69	1476,08	1025,5175	4687,385	-3661,8675
**TAF1D**	13466,46	4932,31	5512,01	8746,95	20092,98	3557,55	8164,4325	11825,265	-3660,8325
**TCF12**	4370,97	5217,74	4690,51	3859,75	4704,03	11686,01	4534,7425	8195,02	-3660,2775
**ZNF469**	316,59	62,51	102,05	434,42	297,44	7479,01	228,8925	3888,225	-3659,3325
**NBR1**	2702,44	5855,8	2267,44	3181,14	4883,9	9437,74	3501,705	7160,82	-3659,115
**NF1**	2727,19	4216,96	1813,98	2316,68	5790,38	7061,14	2768,7025	6425,76	-3657,0575
**RALB**	3581,53	3099,11	3586,55	7078,66	8432,37	7550,47	4336,4625	7991,42	-3654,9575
**PAFAH1B1**	3903,75	6621,51	6737,39	7244,85	14464	5081,13	6126,875	9772,565	-3645,69
**RC3H2**	3633,95	3395,63	2144,92	3594	3823,75	9851,75	3192,125	6837,75	-3645,625
**UBE2Q1**	5955,11	4449,89	6125,19	5410,68	13266,35	4988,58	5485,2175	9127,465	-3642,2475
**EVI2B**	231,7	810,8	128,51	1,39	5250,25	2616,25	293,1	3933,25	-3640,15
**EXOC4**	4400,83	5437,54	5681,99	4153,43	8942,62	8173,97	4918,4475	8558,295	-3639,8475
**FNIP1**	2042,28	1838,74	1139,71	1725,39	5043,96	5589,74	1686,53	5316,85	-3630,32
**INPP4B**	114,84	665,38	373,32	48,38	6044,1	1816,96	300,48	3930,53	-3630,05
**PARP8**	468,8	132,57	698,13	342,01	6229	1841,28	410,3775	4035,14	-3624,7625
**TOPBP1**	9559,19	8431,13	4568,96	6930,44	7287,02	14706,33	7372,43	10996,675	-3624,245
**LRRC8C**	817,32	1147,97	740,34	1046,13	7222,31	1895,94	937,94	4559,125	-3621,185
**RAB34**	8,52	1,65	17,95	3,76	7246,57	8,12	7,97	3627,345	-3619,375
**ATP6V1G1**	5414,76	4261,58	4161,55	3783,13	6997,74	9048,13	4405,255	8022,935	-3617,68
**RNASE6**	729,25	107,12	417,84	81,03	7866,23	29,71	333,81	3947,97	-3614,16
**USP13**	863,67	354,31	145,12	39,82	5394,86	2531,64	350,73	3963,25	-3612,52

**CTSC**	22932,07	10429,76	10009,68	12019,64	22434,51	12468,94	13847,7875	17451,725	-3603,9375
**RPL22L1**	2874,46	3844,11	1210,08	1071,22	10892,88	813,15	2249,9675	5853,015	-3603,0475
**ZFP91**	6352,88	7407,69	4681,59	6014,99	10250,12	9178,5	6114,2875	9714,31	-3600,0225
**NMNAT3**	1398,41	133,27	2196,47	54,82	4047,59	5029,51	945,7425	4538,55	-3592,8075
**GOLM2**	2422,51	2203,29	1757,29	4345,01	6404,61	6142,35	2682,025	6273,48	-3591,455
**HTT**	4242,35	6791,69	3584,66	3418,27	3453,86	12739,53	4509,2425	8096,695	-3587,4525
**CPNE8**	1411,34	510,84	790,84	858,36	8950,58	0	892,845	4475,29	-3582,445
**ITPR1**	5586,27	1798,87	2882,96	4731,94	9735	4918,43	3750,01	7326,715	-3576,705
**TCEA1**	12639,54	9706,18	6530,49	10555,19	19395,23	7470,57	9857,85	13432,9	-3575,05
**HLTF**	8908,37	5453,54	6888,59	8953,2	3284,36	18963,81	7550,925	11124,085	-3573,16
**MAPK14**	7186,61	8491,08	7910,56	6165,26	6571,63	15450,98	7438,3775	11011,305	-3572,9275
**PTTG1IP**	11602,38	15585,21	28018,02	11697,87	27339,02	13256,49	16725,87	20297,755	-3571,885
**PRELID3B**	4643,06	4333,47	3360,88	6248,65	6548,52	9888,18	4646,515	8218,35	-3571,835
**CDHR1**	0	0	2,63	0	7135,07	0	0,6575	3567,535	-3566,8775
**PPIG**	14605,35	8476,1	14546,43	9923,93	21602,4	9299,96	11887,9525	15451,18	-3563,2275
**ARHGAP32**	3,84	0	0	0	1684,55	5434,21	0,96	3559,38	-3558,42
**RMI1**	1144,27	1421,62	419,08	1837,88	1502,49	8024,99	1205,7125	4763,74	-3558,0275
**EXOSC7**	3960,94	2815,98	4158,15	1866,95	12093,83	1376,25	3200,505	6735,04	-3534,535
**CREBZF**	9270,74	8142,2	4892,26	10548,56	11397,01	12095,34	8213,44	11746,175	-3532,735
**ADA2**	28,07	108,09	0	0	2539,38	4569,46	34,04	3554,42	-3520,38
**NCR3LG1**	226,99	1215,78	421,17	1564,1	895,38	7849,2	857,01	4372,29	-3515,28
**ZFHX3**	243,35	672,24	128,96	88,33	88,71	7507,28	283,22	3797,995	-3514,775
**CYBC1**	3598,74	3373,53	10022,23	4190,7	15014,72	2595,74	5296,3	8805,23	-3508,93
**CHCHD10**	1039,31	996,27	883,91	1829,32	9000,48	388,65	1187,2025	4694,565	-3507,3625
**RASSF2**	2094,65	1415,62	3044,04	1025,34	6779,41	4021,18	1894,9125	5400,295	-3505,3825
**TXNL4B**	729,78	1186,5	682,02	1206,33	644,51	8268,35	951,1575	4456,43	-3505,2725
**SPG11**	4385,36	4706,44	4951,44	4150,96	8803,57	7304,01	4548,55	8053,79	-3505,24
**ZNF24**	6747,17	5444,35	2873,41	5545,56	6250,41	11053,72	5152,6225	8652,065	-3499,4425
**CBX1**	5833,08	5731,39	4518	5245,76	8594,12	9067,95	5332,0575	8831,035	-3498,9775
**RBM6**	8172,88	6733,98	12910,71	5588,48	13787,92	9894,01	8351,5125	11840,965	-3489,4525
**DNAJC10**	5109,23	3716,8	1718,59	3596,55	2578,34	11468,58	3535,2925	7023,46	-3488,1675
**SERINC3**	7444,6	10659,69	12140,53	12209,26	8801,7	19393,09	10613,52	14097,395	-3483,875
**ZMYM2**	3044,74	6149,09	2255,75	2757,65	8086,73	5982,32	3551,8075	7034,525	-3482,7175

**PUM2**	9597,56	9182,09	5567,31	4447,43	11692,14	9655,48	7198,5975	10673,81	-3475,2125
**SLC38A2**	12143,28	15579,03	9597,1	13560,2	14525,41	17861,95	12719,9025	16193,68	-3473,7775
**STMP1**	2365,67	3974,7	4133,71	3197,4	7527,61	6239,09	3417,87	6883,35	-3465,48
**CBWD5**	1196,37	1489,88	1433,69	1557,87	7386,74	2362,9	1419,4525	4874,82	-3455,3675
**MCL1**	9135,98	19206,08	14711,93	13130,86	25518,07	9482,45	14046,2125	17500,26	-3454,0475
**RMND5A**	8082,76	7036,55	4402,42	6056,63	5775,01	13915,42	6394,59	9845,215	-3450,625
**GOLPH3**	5683,69	6364,91	8090,87	6998,68	12584,78	7882,71	6784,5375	10233,745	-3449,2075
**MAPKAPK2**	11669,44	5762,93	8851,19	13321,36	21421,96	5274,25	9901,23	13348,105	-3446,875
**DENND1A**	2232,15	2788,46	2042,11	2962,52	10448,18	1457,95	2506,31	5953,065	-3446,755
**SLC39A9**	5675,82	4451,6	3164,19	3951,95	6519,58	8992,01	4310,89	7755,795	-3444,905
**TNRC6C**	601,57	1184,2	988,4	809,46	2357,53	6308,86	895,9075	4333,195	-3437,2875
**SPART**	3612,09	5020,18	1	5647,99	13998,14	11,55	3570,315	7004,845	-3434,53
**KCTD3**	5081,91	2686,34	1364,75	1550,07	5916,68	6270,93	2670,7675	6093,805	-3423,0375
**CENPH**	3267,81	2879,15	1910,2	1385,63	5519,1	6037,59	2360,6975	5778,345	-3417,6475
**ARNT**	2359,13	4731,55	2055,64	3196,46	9023,85	3982,44	3085,695	6503,145	-3417,45
**AKR1B1**	5277,75	4032,64	4706,26	8,03	6539,59	7295,18	3506,17	6917,385	-3411,215
**KLF13**	3968,32	9125,3	4632,61	7170,37	6201,78	13058,22	6224,15	9630	-3405,85
**UCK2**	12634,42	6054,27	4370,66	5677,49	17915,97	3259,05	7184,21	10587,51	-3403,3
**CD68**	1595,02	2076,1	8957,31	15988,28	21017,87	90,2	7154,1775	10554,035	-3399,8575
**SV2A**	13,53	5413,63	10,55	36,61	965,39	8569,33	1368,58	4767,36	-3398,78
**RHOU**	86,63	1,51	16,53	903,66	4522,09	2778,71	252,0825	3650,4	-3398,3175
**YY1AP1**	6042,46	6572,12	7080,28	7918,35	14815,7	5787,33	6903,3025	10301,515	-3398,2125
**GAS7**	0	19,23	17,44	8,1	5831,73	981,45	11,1925	3406,59	-3395,3975
**SPTLC1**	2090,22	3967,9	3226,96	5849,71	5393,08	8945,47	3783,6975	7169,275	-3385,5775
**STX16**	5058,01	6870,95	5822,87	4859,52	5430,61	12643,63	5652,8375	9037,12	-3384,2825
**SORL1**	63,26	121,47	26,29	27,18	2366,24	4509,8	59,55	3438,02	-3378,47
**PPA1**	11416,93	12691,47	4985,27	6405,72	19804,36	4667,95	8874,8475	12236,155	-3361,3075
**ZNF438**	371,47	369,57	554,08	344,69	6968,46	564,05	409,9525	3766,255	-3356,3025
**ADGRL1**	431,78	899,56	1865,91	1381,67	548,01	8442,5	1144,73	4495,255	-3350,525
**PPP3R1**	11318,64	8629,7	6390,8	12260,92	9567,39	16433,12	9650,015	13000,255	-3350,24
**BLM**	4810,78	2585,95	3960,04	4200,1	4397,17	10070,28	3889,2175	7233,725	-3344,5075
**SLC39A6**	1325,7	1419,79	765,3	1200,39	5040,02	4002,64	1177,795	4521,33	-3343,535
**AMZ2**	2220,25	2722,99	2205,71	1362,15	7031,68	3908,78	2127,775	5470,23	-3342,455

**ITFG1**	2837,27	2163,31	1174,26	4906,09	4424	7800,59	2770,2325	6112,295	-3342,0625
**RAB7A**	15391,26	13578,54	13136,64	10963,56	17332,44	15885,25	13267,5	16608,845	-3341,345
**ARPP19**	7278,62	7485,29	3985,59	7743,06	5232,07	14696,82	6623,14	9964,445	-3341,305
**UBE2E1**	7013,85	6896,76	4210,44	5101,19	8511,74	9777,35	5805,56	9144,545	-3338,985
**ACAT2**	8514,12	3626,9	3582,36	7037,93	6297,14	11744	5690,3275	9020,57	-3330,2425
**ZC3H18**	5847,07	6715,47	4469,87	4750,24	4902,1	12648,2	5445,6625	8775,15	-3329,4875
**PTPN2**	3376,2	2710,19	1460,98	3757,65	9985,12	2325,76	2826,255	6155,44	-3329,185
**DBI**	2618,53	2650,15	4800,33	1980,84	10152,32	2529,06	3012,4625	6340,69	-3328,2275
**SFXN1**	4754,16	3468,31	607,2	3561,32	5026,5	7822,74	3097,7475	6424,62	-3326,8725
**HACD4**	0	19,61	460,08	0	1281,83	5611,6	119,9225	3446,715	-3326,7925
**OSBPL5**	807,14	6474,35	3907,12	2049,9	12389,35	878,98	3309,6275	6634,165	-3324,5375
**RHEB**	5312,63	3195,14	3910,68	4153,84	6727,36	8185,97	4143,0725	7456,665	-3313,5925
**MAPK6**	5633,81	2864,13	3569,8	4645,4	7495,76	7480,56	4178,285	7488,16	-3309,875
**ZFP1**	801,53	758,5	328,44	580,2	615,3	7234,94	617,1675	3925,12	-3307,9525
**KIF11**	8691,63	7584,88	2978,12	5479,87	5156,12	13823,88	6183,625	9490	-3306,375
**MAP1LC3B**	3833,07	4415,54	3409,19	3242,73	3870,8	10186,23	3725,1325	7028,515	-3303,3825
**KNL1**	4427,72	2881,1	1821,33	3063,11	3045,09	9652,24	3048,315	6348,665	-3300,35
**TLK1**	3164,46	4239,21	2526,88	3608,38	2417,73	10930,33	3384,7325	6674,03	-3289,2975
**IDH2**	12632,23	27518,85	20772,82	9692,22	10016,67	31855,88	17654,03	20936,275	-3282,245
**COX7A2**	8464,44	6559,73	12305,59	7935,13	16321,75	7862,64	8816,2225	12092,195	-3275,9725
**ZC3HAV1**	8008,97	7566,11	9781,87	6118,07	13627,93	8661,11	7868,755	11144,52	-3275,765
**FBXL5**	1554,02	1980,42	1607,87	2077,71	7046,7	3113,19	1805,005	5079,945	-3274,94
**ITCH**	3080,84	3226,26	1927,55	3869,27	3627,67	8968,11	3025,98	6297,89	-3271,91
**SMC5**	1830,64	3414,18	1339,83	3727,16	2607,02	9088,49	2577,9525	5847,755	-3269,8025
**ACADM**	5540,2	8424,99	4616,12	4396,81	8285,66	9733,84	5744,53	9009,75	-3265,22
**TSC22D3**	53,7	192,59	100,84	58,46	2400,54	4327,88	101,3975	3364,21	-3262,8125
**PLK4**	4649,34	3008,63	1754,56	3320,87	6174,43	6702,08	3183,35	6438,255	-3254,905
**FEZ2**	1116,11	1287,93	1923,32	877,95	8490,79	611,07	1301,3275	4550,93	-3249,6025
**SPAG9**	2874,64	3200,08	1875,83	4206,5	4494,82	8073,39	3039,2625	6284,105	-3244,8425
**ZHX3**	476,87	1747,96	954,93	1813,47	4310,54	4675,03	1248,3075	4492,785	-3244,4775
**PSTPIP1**	6	7,52	1,45	5,06	6493,07	3,1	5,0075	3248,085	-3243,0775
**ZFAND5**	4122,24	7371,04	5148,7	6845,69	10406,82	7811,95	5871,9175	9109,385	-3237,4675
**CENPE**	3923,9	3426,33	2525,36	3683,67	5283,89	7970,11	3389,815	6627	-3237,185

**HLA-C**	5016,37	27482,49	74062,33	5600,59	48890,84	13663,5	28040,445	31277,17	-3236,725
**GTF2H2C**	4207,99	2145,65	842,75	2770,5	6099,73	5352,47	2491,7225	5726,1	-3234,3775
**IKZF1**	5773,21	7667,67	3789,23	8112,24	8655,41	10480,55	6335,5875	9567,98	-3232,3925
**ABR**	368,33	1945,98	25,75	45,73	5117,78	2538,65	596,4475	3828,215	-3231,7675
**DIDO1**	5284,88	7094,08	4883,25	8587,8	3987,04	15399,92	6462,5025	9693,48	-3230,9775
**SRGAP2B**	801,2	457,69	308,85	1903,93	6552,4	1643,42	867,9175	4097,91	-3229,9925
**MX2**	0	8,55	150,91	0	3081,42	3453,57	39,865	3267,495	-3227,63
**C4orf3**	5493,72	5558,78	5410,33	3727,55	11220,91	5314,54	5047,595	8267,725	-3220,13
**MAP1LC3A**	112,49	0	1,78	152,05	6547,29	0,85	66,58	3274,07	-3207,49
**KNTC1**	11180,09	6419,66	4869,78	5486,56	9277,45	11114,5	6989,0225	10195,975	-3206,9525
**STAU1**	7050,87	6908,76	6582,32	11377,14	5371,08	16997,67	7979,7725	11184,375	-3204,6025
**ING1**	2163,71	1457,65	2346,84	1234,55	1789,63	8213,99	1800,6875	5001,81	-3201,1225
**VPS35**	9805,39	7716,96	6670,2	24962,61	9109,89	21868,96	12288,79	15489,425	-3200,635
**PRKAA1**	2724,65	2499,63	4280,5	2903,51	8653,77	3934,68	3102,0725	6294,225	-3192,1525
**EFHD2**	3675,92	6705,71	6343,78	6334,78	10350,44	7559,23	5765,0475	8954,835	-3189,7875
**GLIPR1**	71,2	29,01	177,13	211,74	6219,43	400,57	122,27	3310	-3187,73
**MRPL44**	2444,98	2887,64	2182,84	2858,39	9934,86	1625,29	2593,4625	5780,075	-3186,6125
**ACAP2**	2425,44	2650,74	1137,4	3237	5427,71	5670,47	2362,645	5549,09	-3186,445
**MID1IP1**	10419,59	7827,93	3231,88	3551,68	12360,41	6525,59	6257,77	9443	-3185,23
**PYCARD**	3135,65	2299,2	5156,05	3285,16	11457,04	1850,17	3469,015	6653,605	-3184,59
**ZNF131**	3778,59	2520,75	5951,85	2507,94	9515,62	4207,16	3689,7825	6861,39	-3171,6075
**LEO1**	4445,72	3140,75	3705	3231,64	8246,5	5354,97	3630,7775	6800,735	-3169,9575
**ATP8B2**	3281,9	1982,64	3402,39	2640,04	2272,88	9718,55	2826,7425	5995,715	-3168,9725
**ECT2**	2323,68	1794,9	1344,37	2989,2	3116,54	7447,19	2113,0375	5281,865	-3168,8275
**WASF1**	777,37	6583,67	1639,18	3094,54	3012,61	9366,83	3023,69	6189,72	-3166,03
**CHMP1A**	4688,8	8107,88	5558,74	4820,45	4303,64	13609,95	5793,9675	8956,795	-3162,8275
**PDE4D**	7,54	6,69	882,67	96,97	5423,35	1396,08	248,4675	3409,715	-3161,2475
**HS2ST1**	5044,22	4761,6	3564,47	6528,13	3345,18	12911,33	4974,605	8128,255	-3153,65
**BROX**	2869,75	2014,69	1279,97	1833,96	5421	4882,55	1999,5925	5151,775	-3152,1825
**RECQL**	2833,1	3607,98	2206,62	3771,41	2477,87	10030,36	3104,7775	6254,115	-3149,3375
**ADAP1**	478,98	407,29	42,62	119,7	6286,27	535,42	262,1475	3410,845	-3148,6975
**YME1L1**	9857,47	11221,97	9381,05	12131,37	12887,44	14704,68	10647,965	13796,06	-3148,095
**AIMP1**	6809,87	4702,68	4115,56	5766,67	11989,74	5002,58	5348,695	8496,16	-3147,465

**MT1F**	0	85,13	87,12	217,28	6489,26	0	97,3825	3244,63	-3147,2475
**C5orf22**	1551,67	1071,72	1708,73	1696,75	5049,98	4245,85	1507,2175	4647,915	-3140,6975
**PTGER4**	7,07	1424,96	75,3	85,53	6038,78	1038,09	398,215	3538,435	-3140,22
**ZKSCAN1**	3983,59	4695,26	3695,87	3549,44	8529,05	5710,73	3981,04	7119,89	-3138,85
**HIF1A**	3630,63	4608,73	2541,3	3152,54	5335,62	7907,54	3483,3	6621,58	-3138,28
**SRCAP**	5159,82	10071,96	2845,83	7257,35	2310,44	16633,2	6333,74	9471,82	-3138,08
**MARCHF7**	5340,46	6126,51	2960,73	7013,19	7859,88	9127,29	5360,2225	8493,585	-3133,3625
**MAGEF1**	895,43	1755,37	728,23	1413,44	2391,12	6264,34	1198,1175	4327,73	-3129,6125
**IREB2**	8377,08	6135,84	6013,87	6195,29	7286,07	12320,41	6680,52	9803,24	-3122,72
**ANO6**	4659,34	3365,91	2097,1	4827,73	6833,48	6877,34	3737,52	6855,41	-3117,89
**FRY**	1545,64	616,61	770,09	1064,63	7230,6	995,88	999,2425	4113,24	-3113,9975
**MTDH**	16628,94	14062,32	9626,08	13124,3	25316,3	7632,16	13360,41	16474,23	-3113,82
**COPS6**	8282,49	4872,12	6016,81	5432,03	13364,08	5163,96	6150,8625	9264,02	-3113,1575
**PCMTD2**	1901,57	2773,18	2038,48	3043,36	1870,61	9229,07	2439,1475	5549,84	-3110,6925
**RAB24**	3115,11	3641,91	4500,8	4823,18	12370,2	1878,49	4020,25	7124,345	-3104,095
**SPATA6**	0	0	9,46	0	5290,79	922,08	2,365	3106,435	-3104,07
**NCAPD2**	18206,19	18836,06	9556,85	12389,42	14447,8	21253,23	14747,13	17850,515	-3103,385
**AGT**	165,76	53,6	104,89	20,6	6309,54	64,76	86,2125	3187,15	-3100,9375
**CDK19**	1385,38	1596,71	2197,09	4673,57	2757,73	8363,54	2463,1875	5560,635	-3097,4475
**PRDX2**	11528,53	8230	8008,82	4102,67	12879,34	9246,31	7967,505	11062,825	-3095,32
**HIPK1**	6697,11	6282,6	5826,37	9716,65	8123,27	12327,16	7130,6825	10225,215	-3094,5325
**TADA3**	6054,42	8985,04	8837,89	6189,31	9900,35	11321,5	7516,665	10610,925	-3094,26
**LARP7**	5443,41	4494,47	6151,64	4271,07	10211,23	6150,49	5090,1475	8180,86	-3090,7125
**E2F7**	123,22	1081,88	36,06	7,65	48,31	6748,16	312,2025	3398,235	-3086,0325
**MAD1L1**	1295,88	4046,59	3508,72	8118,72	11903,09	2741,12	4242,4775	7322,105	-3079,6275
**KDM5A**	1950,2	6451,39	4417,83	3317,78	7579,87	6646,61	4034,3	7113,24	-3078,94
**AAK1**	709,03	836,04	1562,63	2226,77	4600,51	4214,85	1333,6175	4407,68	-3074,0625
**ARID4A**	2056,19	2588,9	2366,67	2489,26	7709,47	3182,66	2375,255	5446,065	-3070,81
**TICRR**	4610,85	4447,47	1895,76	3474,09	2563,22	10788,57	3607,0425	6675,895	-3068,8525
**ERCC5**	7519,76	6278	14560,97	5367,13	7759,37	15221,35	8431,465	11490,36	-3058,895
**PIKFYVE**	4265,74	4106,51	2922,02	4989,96	5813,4	8443,24	4071,0575	7128,32	-3057,2625
**PPP1CB**	9996,47	7980,06	5167,43	5933,29	11762,16	8858,88	7269,3125	10310,52	-3041,2075
**AZI2**	1658,4	2230,2	1389,38	1673,94	6435,19	3114,08	1737,98	4774,635	-3036,655

**TBC1D1**	3491,47	3362,83	2161,01	3090,72	5874,23	6241,18	3026,5075	6057,705	-3031,1975
**IAH1**	3	12,87	264,28	839,9	5602,32	1006,15	280,0125	3304,235	-3024,2225
**PALD1**	16,79	38,47	65,2	2,25	4846,38	1252,17	30,6775	3049,275	-3018,5975
**DOC2A**	81,58	40,23	117,08	97,46	638,21	5551,7	84,0875	3094,955	-3010,8675
**GSK3B**	2803,84	2839,18	1493,61	1914,14	3046,46	7495,33	2262,6925	5270,895	-3008,2025
**TCIRG1**	7093,79	2930,5	1184,94	8099,28	14965,98	684,91	4827,1275	7825,445	-2998,3175
**GID8**	6143,39	6242,98	6444,76	7426,18	6018,91	13094,91	6564,3275	9556,91	-2992,5825
**UBR5**	15178,77	11353,13	14160,2	12135,79	26234,7	6161,43	13206,9725	16198,065	-2991,0925
**MBTD1**	2885,85	4254,56	3597,37	3325,78	4060,25	8943,61	3515,89	6501,93	-2986,04
**CAMK1D**	1525,06	2079,25	225,75	1724,19	3487,77	5260,72	1388,5625	4374,245	-2985,6825
**LY75**	186,51	0	661,19	782,12	3116,5	3668,25	407,455	3392,375	-2984,92
**FAM102B**	1783,89	3426,21	1374,82	3580,96	4519,66	6531,84	2541,47	5525,75	-2984,28
**ZFP90**	495,6	932,02	1850,97	605,94	1140,29	6761,65	971,1325	3950,97	-2979,8375
**TOMM7**	2425,87	4935,6	7398,06	3683,15	13474,98	1705,89	4610,67	7590,435	-2979,765
**GNG10**	1157,08	943,69	861,38	1042,07	3886,14	4072,83	1001,055	3979,485	-2978,43
**TMEM65**	1646,78	6639,76	1113	1659,89	6726,22	4753,52	2764,8575	5739,87	-2975,0125
**VEZF1**	2780,51	2233,38	2219,19	2725,35	3955,26	6973,96	2489,6075	5464,61	-2975,0025
**BDP1**	4998,78	3136,93	2301,16	4741,89	6990,64	6547,97	3794,69	6769,305	-2974,615
**NTNG2**	0	28,27	22,06	6,49	64,22	5911,19	14,205	2987,705	-2973,5
**BOD1L1**	7943,7	6570,62	5104,04	6827,31	8965,19	10203,57	6611,4175	9584,38	-2972,9625
**MTREX**	10118,28	8685,61	5056,13	5939,92	8543,8	12292,85	7449,985	10418,325	-2968,34
**PLXNB2**	6639,35	23169,55	1259,5	6994,84	10428,53	14538,04	9515,81	12483,285	-2967,475
**PHF21A**	2323,36	2451,49	2755,49	3420,32	7937,11	3470,83	2737,665	5703,97	-2966,305
**PM20D2**	2950,6	385,47	290,19	36,01	3077,66	4685,37	915,5675	3881,515	-2965,9475
**SACM1L**	4202,13	4095,3	2505,28	2418,35	4790,95	7744,08	3305,265	6267,515	-2962,25
**ADGRE5**	1923,08	5399,32	2046,59	3225,97	11912,07	293,1	3148,74	6102,585	-2953,845
**POGZ**	4783,23	6097,37	3638,67	5253,08	6172,61	9612,68	4943,0875	7892,645	-2949,5575
**RYBP**	2894,02	5386,04	5042,49	5884,45	4488,66	11013,5	4801,75	7751,08	-2949,33
**PI4KA**	13610,84	14503,34	12426,7	8323,93	18772,71	11557,28	12216,2025	15164,995	-2948,7925
**FAM72A**	3384,44	1054,68	942,45	2730,12	6465,61	3485,06	2027,9225	4975,335	-2947,4125
**ARHGEF40**	590,74	41,28	66,46	278,28	1962,36	4410,35	244,19	3186,355	-2942,165
**WDHD1**	5223	3626,27	1270,1	2630,7	2657,69	9601,24	3187,5175	6129,465	-2941,9475
**OTUD4**	2910,37	3825,67	2074,29	2702,96	5910,49	5729,06	2878,3225	5819,775	-2941,4525

**AGPAT2**	3407,57	2437,59	1673,41	2469,84	6899,12	3962,22	2497,1025	5430,67	-2933,5675
**PSMA2**	12016,88	8042,37	9868,38	8462,66	16739,2	8314,87	9597,5725	12527,035	-2929,4625
**PAIP2**	8083,68	7046,87	6400,86	6706,73	11101,64	8875,03	7059,535	9988,335	-2928,8
**GNB4**	4254,18	3097,9	3098,76	4031,57	4580,53	8513,46	3620,6025	6546,995	-2926,3925
**CNST**	3216,32	3453,84	1438,95	3830,49	7579,95	4239,4	2984,9	5909,675	-2924,775
**CD38**	1429,41	1192,83	6047,94	102,98	2556,72	7677,38	2193,29	5117,05	-2923,76
**EIF2A**	8067,27	8347,88	4180,18	5927,58	14234,08	4872,45	6630,7275	9553,265	-2922,5375
**EIF3CL**	571,25	298,38	1536,41	287,76	6466,74	721,81	673,45	3594,275	-2920,825
**DOT1L**	9680,83	12235,45	4316,82	4471,6	10227,61	10950,61	7676,175	10589,11	-2912,935
**CEP350**	5106,02	3668,79	2083,9	5118,56	6998,25	6814,17	3994,3175	6906,21	-2911,8925
**DZANK1**	153,95	111,17	171,26	60,01	5772,78	297,11	124,0975	3034,945	-2910,8475
**BNIP2**	4646,16	5146,71	6645,81	6786,11	10442,96	6986,5	5806,1975	8714,73	-2908,5325
**ME2**	5817,09	4920,03	2487,44	5798,25	10722,5	4593,28	4755,7025	7657,89	-2902,1875
**DUSP7**	3784,93	4318,63	2305,58	2380,82	9727,85	2451,73	3197,49	6089,79	-2892,3
**B4GALT6**	527,99	289,32	69,8	371,65	0	6406,12	314,69	3203,06	-2888,37
**KIAA1143**	3466,82	2911,78	2283,86	2135,91	3841,88	7332,14	2699,5925	5587,01	-2887,4175
**HIRIP3**	4088,66	3836,71	2371,65	3131,29	4117,11	8361,22	3357,0775	6239,165	-2882,0875
**C2CD5**	2459,44	3105,25	2521,07	1565,94	5933,6	4652,36	2412,925	5292,98	-2880,055
**RPLP2**	58136,23	54413,73	77512,34	48074,59	103896,68	20928,51	59534,2225	62412,595	-2878,3725
**HNRNPU**	85885,49	62685,14	51340,82	66268,78	83747,53	55087,33	66545,0575	69417,43	-2872,3725
**PFKFB3**	3033,74	1857,86	3941,7	2328,37	8608,41	2714,43	2790,4175	5661,42	-2871,0025
**PLAUR**	904,53	1427,76	1455,82	1380,49	7848,17	477,99	1292,15	4163,08	-2870,93
**NDUFA5**	4277	2935,78	3151,01	3703,24	5262,02	7507,45	3516,7575	6384,735	-2867,9775
**MAD2L1**	9698,83	5718,62	3228,24	5571,38	9163,13	8680,12	6054,2675	8921,625	-2867,3575
**STRBP**	3259,23	3382,7	1367,02	4174,17	999,26	10802,28	3045,78	5900,77	-2854,99
**TBC1D14**	4840,58	6027,24	2779,72	5645,89	8382,26	6972,72	4823,3575	7677,49	-2854,1325
**RGS19**	4333,93	1297,29	4699,82	5912,68	8087,79	5740,83	4060,93	6914,31	-2853,38
**CEBPB**	2583,76	1327,65	3213,2	3983,74	8198,65	3056,59	2777,0875	5627,62	-2850,5325
**ERCC6L2**	1005,56	1406,21	509,05	996,91	973,65	6681,52	979,4325	3827,585	-2848,1525
**ANKHD1**	8103,4	8004,69	7907,64	7858,96	12159,08	9459,65	7968,6725	10809,365	-2840,6925
**DBNDD2**	240,85	987,48	299,43	397,51	1194,05	5448,59	481,3175	3321,32	-2840,0025
**HNMT**	122,37	0	175,92	67,73	5860,34	2,28	91,505	2931,31	-2839,805
**FOXK1**	3731,87	5296,18	3626,17	4833,34	7276,5	7141,53	4371,89	7209,015	-2837,125

**POLE3**	9887,81	9099,65	6136,38	7984,99	8164,31	14063,58	8277,2075	11113,945	-2836,7375
**ARHGAP17**	3173,26	2385,31	3714,9	2846,6	8793,8	2938,77	3030,0175	5866,285	-2836,2675
**UBE2K**	6266,45	6680,57	3388,36	7274,38	8504,12	8969,93	5902,44	8737,025	-2834,585
**VAPB**	2203,83	3300,99	3288,43	2682,37	3188,85	8202,95	2868,905	5695,9	-2826,995
**CNOT8**	7542,53	6401,88	4847,61	7379,58	10785,17	7952,43	6542,9	9368,8	-2825,9
**TMEM167A**	6938,05	5229,89	5896,53	7347,4	12599,69	5755,78	6352,9675	9177,735	-2824,7675
**ATP5PD**	6682,5	5299,78	9295,66	6190,57	14007,65	5363,89	6867,1275	9685,77	-2818,6425
**AURKA**	5384,68	4035,97	3714,58	5101,97	4468,36	10274,91	4559,3	7371,635	-2812,335
**DESI2**	3656,63	2159,92	945,85	1991,49	3916,62	6080,31	2188,4725	4998,465	-2809,9925
**CUL4B**	4138,46	2915,97	4068,37	3456,86	6660,8	6242,39	3644,915	6451,595	-2806,68
**RASGRP2**	6093,26	4899,79	21863,29	8599,82	15606,41	10734,75	10364,04	13170,58	-2806,54
**GBA**	2412,52	3418,92	3132,3	2923,79	4998,08	6558,62	2971,8825	5778,35	-2806,4675
**NAIP**	2657,62	1089,41	1810,83	1654,3	6654,57	2561	1803,04	4607,785	-2804,745
**SLC37A2**	58,4	20,08	25,36	76,57	5359,02	323,99	45,1025	2841,505	-2796,4025
**FANCD2**	6187,79	5053,85	3111,42	2564,7	4971,26	9077,71	4229,44	7024,485	-2795,045
**PSMB10**	2886,02	4315,53	4966,78	3617,61	11888,56	1592,32	3946,485	6740,44	-2793,955
**TMEM87A**	3028,98	2534,26	3174,32	2432,56	8071,57	3098,44	2792,53	5585,005	-2792,475
**DEF6**	2984,74	4716,85	7245,18	4955,58	5726,25	9807,96	4975,5875	7767,105	-2791,5175
**BRAF**	1617,37	1909,08	1251,28	1644,07	5615,91	3176,17	1605,45	4396,04	-2790,59
**AKR7A2**	2965,12	5754,5	3930,8	2354,21	7224,12	5858,04	3751,1575	6541,08	-2789,9225
**NUP50**	8238,2	9049,74	4175,29	8479,86	8070,05	12480,12	7485,7725	10275,085	-2789,3125
**RNF40**	9532,75	10544,94	11068,7	8817,54	15387,86	10167,37	9990,9825	12777,615	-2786,6325
**ZNF618**	32,46	1183,35	894,17	2379,81	2231,14	5581,07	1122,4475	3906,105	-2783,6575
**XRN1**	1694,6	2085,06	1170,05	1412,25	4296,6	4446,02	1590,49	4371,31	-2780,82
**PRIM1**	3725,87	3372,8	1086,57	2460,98	3149,99	7733,5	2661,555	5441,745	-2780,19
**STAG1**	4603,13	3037,39	1014,82	1627,85	1632,38	9068,63	2570,7975	5350,505	-2779,7075
**UBR7**	4775,38	5053,89	1992,61	4749,43	3584,37	10253	4142,8275	6918,685	-2775,8575
**CLK2**	5618,19	3862,83	4671,68	5111,45	10615,36	4565,9	4816,0375	7590,63	-2774,5925
**NXT2**	1072,87	1246,02	806,93	1649,3	4584,85	3344,27	1193,78	3964,56	-2770,78
**DLGAP5**	3605,93	5109,84	2550,48	3674,67	3210,78	9800,35	3735,23	6505,565	-2770,335
**MIS18BP1**	4038,78	4913,34	2382,12	4156,16	7009,86	6274,24	3872,6	6642,05	-2769,45
**AKT3**	80,08	20,46	20,9	13,78	5559,49	29,63	33,805	2794,56	-2760,755
**SEPTIN5**	181,11	1634,78	0	81,17	4121,13	2343,72	474,265	3232,425	-2758,16

**CNOT1**	21457,76	24938,4	10370,17	16901,25	10472,02	31871,42	18416,895	21171,72	-2754,825
**LMBR1**	2082,78	3142,87	1602,03	2009,61	3131,12	6789,04	2209,3225	4960,08	-2750,7575
**TLE4**	89,94	989,17	5,14	248,03	3123,36	3040,11	333,07	3081,735	-2748,665
**NFATC2**	717,51	3413,93	591,09	2762,16	0	9237,94	1871,1725	4618,97	-2747,7975
**PDCD6IP**	5745,85	13507,7	8602,16	7479,65	12644,87	10513,37	8833,84	11579,12	-2745,28
**FAM120A**	5116,16	17317,68	4448,48	16881,91	12745,83	14622,16	10941,0575	13683,995	-2742,9375
**GBE1**	4323,69	10600,55	5118,67	2476,17	9581,81	7158,7	5629,77	8370,255	-2740,485
**LHX2**	0	0	1,18	0	5481,15	0	0,295	2740,575	-2740,28
**LSM14B**	4273,71	5888,15	5298,16	5397,88	5236,76	10671,48	5214,475	7954,12	-2739,645
**FEV**	22,1	16,6	226,82	5360,04	0	8291,58	1406,39	4145,79	-2739,4
**RAB1A**	7127,25	5150,69	6050,78	4944,58	11628,31	5481,43	5818,325	8554,87	-2736,545
